# Age at Iatrogenic Menopause and Cardiovascular Morbidity and Mortality: A Systematic Review and Meta-Analysis

**DOI:** 10.1001/jamanetworkopen.2026.37545

**Published:** 2026-10-02

**Authors:** Eleni Armeni, Ipek Betul Ozcivit Erkan, Punith Kempegowda

**Affiliations:** 1 Department of Applied Health Sciences, School of Health Sciences, College of Medicine and Health, University of Birmingham, Birmingham, United Kingdom; 2 Royal Free Hospital NHS Foundation Trust, London, United Kingdom; 3 Cerrahpasa Faculty of Medicine, Institute of Graduate Studies, Istanbul University–Cerrahpasa, Istanbul, Turkey; 4 Department of Endocrinology, Queen Elizabeth Hospital, University Hospitals Birmingham, Birmingham, United Kingdom

## Abstract

**Importance:**

Iatrogenic menopause is common, but its long-term cardiovascular consequences remain uncertain, particularly by age at menopause.

**Objective:**

To quantify associations between iatrogenic menopause and cardiovascular morbidity and mortality and evaluate whether age at iatrogenic menopause is a modifier.

**Data Sources:**

In this systematic review and meta-analysis, Scopus, MEDLINE, PubMed, Embase, Cochrane Central Register of Controlled Trials (CENTRAL), CINAHL, and PsycINFO were searched from January 2004 to March 2026.

**Study Selection:**

Observational studies and randomized clinical trials reporting cardiovascular morbidity or mortality among women with iatrogenic menopause before natural menopause age compared with naturally menopausal or age-matched controls were included.

**Data Extraction and Synthesis:**

Data were extracted in duplicate by 2 independent reviewers, with consensus resolution. The study followed the Preferred Reporting Items for Systematic Reviews and Meta-Analyses reporting guideline.

**Main Outcomes and Measures:**

Primary outcomes were cardiovascular morbidity, cardiovascular and all-cause mortality, and a composite of cardiovascular morbidity and cardiovascular mortality, quantified as random-effects pooled hazard ratios (PHRs) with 95% CIs, irrespective of heterogeneity and with age at menopause as a moderator. Meta-regression estimates were clustered by source cohort.

**Results:**

Of 14 409 records, 36 studies were included with 2 617 942 women (mean [SD] age, 55.52 [12.41] years for the iatrogenic menopause cohort and 45.58 [16.02] years for comparators); median follow-up was 16.0 years (range, 4.8-36.0 years). Iatrogenic menopause vs comparators was associated with higher risk of all-cause mortality (PHR, 1.08; 95% CI, 1.01-1.16), cardiovascular events (PHR, 1.25; 95% CI, 1.12-1.39), coronary heart disease (PHR, 1.48; 95% CI, 1.07-2.03), and stroke (PHR, 1.22; 95% CI, 1.15-1.28). Mortality excesses were confined to iatrogenic menopause before age 45 years (cardiovascular mortality: PHR, 1.20 [95% CI, 1.04-1.37]; all-cause mortality: PHR, 1.15 [95% CI, 1.06-1.25]), with null estimates at or after age 45 years (cardiovascular mortality: PHR, 0.95 [95% CI, 0.81-1.11]; all-cause mortality: PHR, 0.99 [95% CI, 0.89-1.09]). In a post hoc meta-regression on the composite outcome, each increasing 5-year increment in age at iatrogenic menopause was associated with lower risk (HR, 0.89; 95% CI, 0.83-0.97) based on 37 estimates from 11 independent populations.

**Conclusions and Relevance:**

In this study, iatrogenic menopause was associated with higher cardiovascular morbidity, most consistently stroke, and with cardiovascular and all-cause mortality excesses before age 45 years at menopause. Older age at iatrogenic menopause was associated with progressively lower risk of cardiovascular morbidity and mortality, supporting a period of elevated risk before age 45 years rather than a single threshold age.

## Key Points

**Question:**

Is iatrogenic menopause associated with cardiovascular (CV) morbidity and mortality, and does age at menopause modify this risk?

**Findings:**

In this systematic review and meta-analysis of 36 studies and 2 617 942 women, iatrogenic menopause was associated with higher risk of composite CV events, coronary heart disease, stroke, and all-cause mortality. Excess mortality was concentrated among women younger than 45 years at iatrogenic menopause.

**Meaning:**

The findings suggest elevated risk of CV mortality below age 45 years at iatrogenic menopause rather than at a threshold age.

## Teaser

This systematic review and meta-analysis examines whether iatrogenic menopause, compared with natural menopause, is associated with increased cardiovascular morbidity and mortality and whether associations differ by age at iatrogenic menopause.

## Introduction

Surgical and other forms of iatrogenic menopause lead to abrupt loss of ovarian hormones, which is associated with adverse long-term health outcomes.^1,2,3^ Women undergoing bilateral salpingo-oophorectomy (BSO), whether for benign indications or as risk-reducing surgery in *BRCA1/2* carriers, experience earlier and more sudden menopause than those with natural menopause.^4,5^ Several studies have reported increased risk of cardiovascular disease (CVD), coronary heart disease, stroke, metabolic syndrome, and all-cause mortality following this abrupt hormonal decline.^6,7,8^ However, some cohorts showed no excess long-term cardiovascular risk after hysterectomy with or without oophorectomy.^9^ Evidence for chemotherapy- or radiotherapy-induced menopause is similarly heterogenous.^10,11,12^

Important uncertainties remain. Studies have used heterogenous comparators, mixing age-matched controls and naturally menopausal women,^6^ and the role of menopausal hormone therapy (MHT) has rarely been evaluated. Prior studies focused on BSO at hysterectomy or ovarian conservation, with limited evaluation of cardiovascular risk, morbidity, or cause-specific and all-cause mortality.^13,14,15,16,17,18,19,20^ Inconsistent age cutoffs and limited consideration of baseline cardiovascular risk complicate interpretation,^7,16,18^ leaving uncertainty about differences by menopause type, age, and MHT use.

Existing evidence suggests comparable cardiometabolic risk in the context of premature ovarian insufficiency (POI) and early menopause^21^ but without cohort-specific analysis of available evidence. Large prospective cohort studies showed higher CVD risk in women experiencing menopause before age 45 years, partially attenuated by healthy lifestyles.^21,22^ Gradual ovarian senescence may mitigate long-term vascular harm compared with abrupt hormonal deprivation.^22^

Whether iatrogenic menopause increases cardiovascular risk and whether that risk varies with age at menopause are clinically important given the high prevalence of hysterectomy, with up to half of procedures including BSO,^23,24,25,26,27^ and the increasing number of women living with treatment-induced menopause due to improved cancer survival.^10,11,12^ As CVD is the leading cause of death in postmenopausal women,^28^ clarifying the cardiovascular consequences of iatrogenic menopause is essential for surgical decisions, cancer care, long-term follow-up, and MHT guidance.

This systematic review and meta-analysis evaluated cardiovascular event and mortality outcomes among women with iatrogenic menopause (surgical or medically induced) compared with naturally menopausal women or age-matched controls. We further assessed age at iatrogenic menopause, type of iatrogenic menopause, and MHT use as modifiers, using meta-regression to examine age at iatrogenic menopause on a continuous scale.

## Methods

### Search Strategy and Selection Criteria

This systematic review and meta-analysis was reported in accordance with the Preferred Reporting Items for Systematic Reviews and Meta-Analyses (PRISMA) 2020 statement, including eligibility criteria, search strategy, study selection, data collection, risk-of-bias assessment, effect measures, synthesis methods, certainty assessment, and study selection flow (Figure 1). The protocol was prospectively registered in PROSPERO. Scopus, MEDLINE, PubMed, Embase, the Cochrane Central Register of Controlled Trials (CENTRAL), CINAHL, and PsycINFO were searched from January 2004 to March 2026 for observational studies and randomized clinical trials (RCTs) reporting cardiovascular morbidity or mortality in women with iatrogenic menopause before natural menopause age vs naturally menopausal women or age-matched controls.

**Figure 1.**
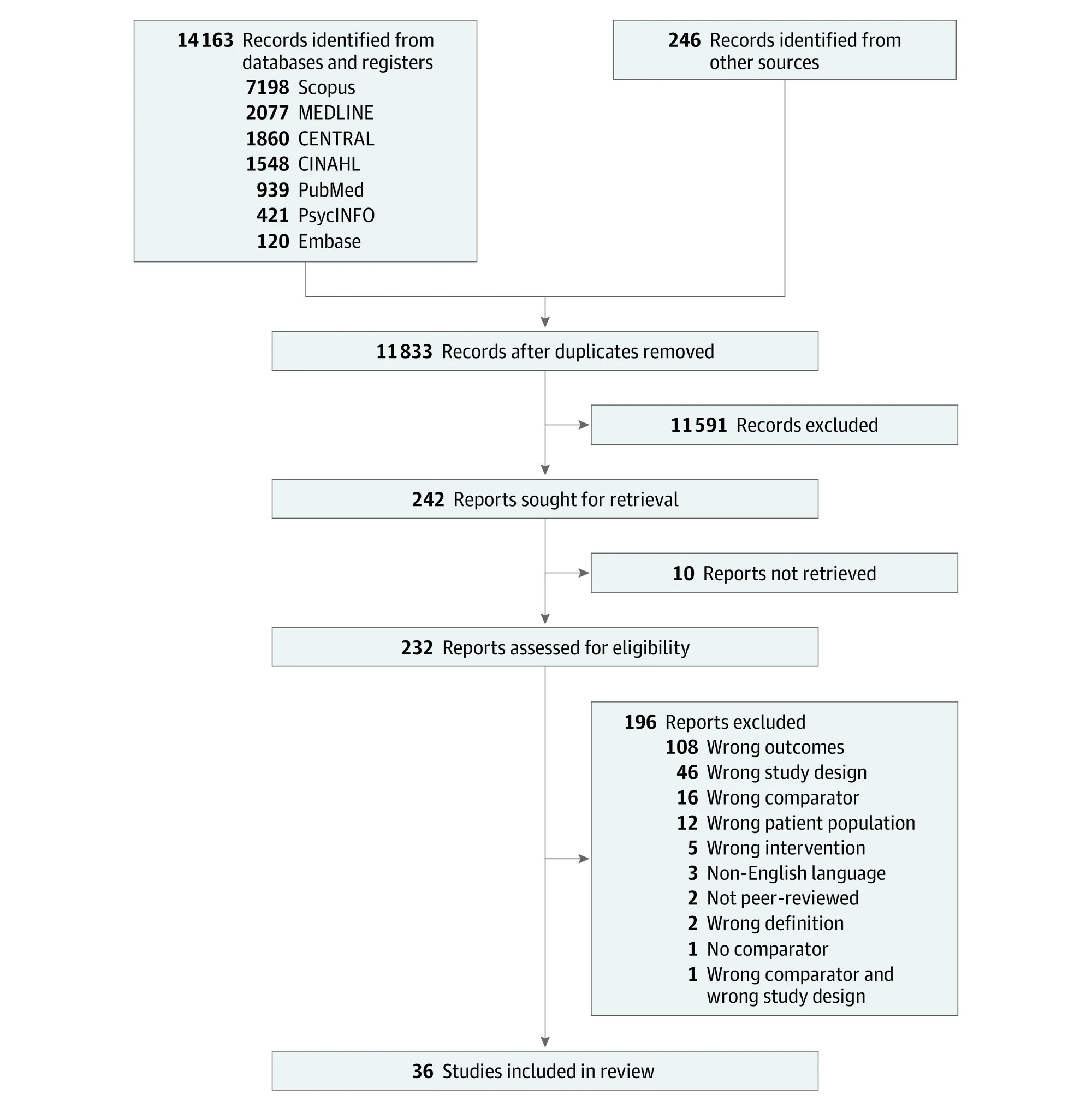
PRISMA Flow Diagram of Study Identification, Screening, Eligibility Assessment, and Inclusion CENTRAL indicates Cochrane Central Register of Controlled Trials.

### Data Synthesis

Two reviewers (E.A., I.B.O.E.) independently extracted data using a standardized form, with consensus resolution (eMethods and eAppendix in Supplement 1). Race and ethnicity data were extracted as reported by the primary studies to describe the generalizability of the included populations; the investigators of those studies determined the classification method and the categories used, and it was not possible to ascertain whether categories were investigator-defined or self-reported. Risk of bias was assessed independently using Cochrane RoB 2.0 for RCTs^29^ and the Newcastle-Ottawa Scale for observational studies and was categorized as low, moderate, or high (eMethods and eTables 1-3 in Supplement 1).

### Statistical Analysis

All eligible estimates appear as originally reported (eTables 4-8 in Supplement 1) and were compared narratively by baseline cardiovascular risk, surgical indication, design, and adjustment strategy. Pooling and meta-regression were restricted to HRs, combined on the natural-log scale and back-transformed for presentation; thus, fewer than the total number of included studies contributed to each estimate. Studies reporting other measures were retained and synthesized narratively. When 2 or more studies reported comparable exposures and outcomes, HRs were pooled by generic inverse variance using random-effects models with restricted maximum-likelihood estimation. Random-effects estimates are primary for every outcome irrespective of heterogeneity; fixed-effect estimates are given for comparison only (eTable 9 in Supplement 1). Heterogeneity was quantified by τ^2^, Cochran *Q*, and *I*^2^ and was examined by leave-1-out omission of each cohort (eTables 16-18 in Supplement 1) and by stratification on comparator type, which included natural menopause, age-matched cohorts, hysterectomy only, or no surgery (eFigures 1-5 in Supplement 1). Comparator handling, within-study combination, and referent-purity restrictions are detailed in the eMethods in Supplement 1.

Age at iatrogenic menopause was examined at the clinically prespecified cutoff of 45 years by subgroup meta-analysis with a formal cohort-level contrast across the cutoff and as a continuous moderator by meta-regression. Meta-regression used age at iatrogenic menopause as the sole covariate and was fitted separately for all-cause mortality, for cardiovascular mortality, and post hoc, for the composite of cardiovascular morbidity and cardiovascular mortality, in which 1 end point was used per source population so that no population contributed twice. Each meta-regression used a 2-stage approach: a slope was estimated within each contributing cohort, and these within-cohort slopes were pooled by random effects with the Knapp-Hartung adjustment so that each source population contributed a single value. Models treating age bands as independent observations were also fitted but were anticonservative because bands from the same cohort were correlated; thus, they are reported only for comparison. Linear models were retained. The direction of the gradient was corroborated by 2 distribution-free analyses: a precision-weighted Kendall rank correlation of the age-band estimates within populations and an exact sign-flip permutation test on the population-specific slopes. The gradient within the range below age 45 years was estimated post hoc among cohorts reporting more than 1 band below that age; this was estimable for all-cause mortality but not for cardiovascular mortality, for which only 1 cohort qualified. MHT was examined as a prespecified exploratory moderator at cohort level by prevalence and within cohorts by MHT stratum (eTables 22 and 23 in Supplement 1). Model specifications assigned age-band midpoints; sensitivity analyses and full results are given in the eMethods and eResults in Supplement 1, with estimates given in eTables 19 to 21 in Supplement 1.

Data were analyzed using R, version 4.6.1 (R Project for Statistical Computing). Statistical significance was defined by a 2-sided α level of 0.05.

## Results

From 14 409 records identified, 36 studies (2 617 942 women) were included: 12 (33.3%) were retrospective cohort studies,^9,16,20,30,31,32,33,34,35,36,37,38^ 11 (30.6%) were prospective cohort studies,^6,7,15,17,18,19,39,40,41,42,43^ 10 (27.8%) were cross-sectional studies,^8,14,44,45,46,47,48,49,50,51^ 2 (5.6%) were RCTs,^52,53^ and 1 (2.8%) was a case-control study^54^ (Figure 1). The included studies were mainly multicenter or registry based or included large prospective cohorts. Nineteen studies (52.8%) evaluated BSO with or without hysterectomy^6,8,14,15,16,17,19,20,32,40,41,42,44,45,47,48,49,53,54^ (1 additional study [2.8%] also included chemotherapy-induced menopause^46^). Twelve studies (33.3%) focused on BSO^7,9,18,31,35,36,37,38,39,43,50,52^ and 2 (5.6%) on *BRCA1/2*-related risk-reducing oophorectomy.^30,33^ Two studies (5.6%) focused on cancer treatment–induced POI.^34,51^ Comparator groups varied widely (natural menopause, age-matched cohorts, hysterectomy only, or no surgery) (eTable 11 in Supplement 1). Race and ethnicity were reported by 18 studies (50.0%)^6,7,9,14,16,17,18,19,20,31,35,36,38,39,40,43,52,53^; categories across these studies included American Indian, Asian or Pacific Islander, Black or African American, Chinese (1 study^20^), Hispanic, White, and multiracial (eTable 11 in Supplement 1).

Age was higher among women with iatrogenic menopause than among comparator groups (mean [SD], 55.52 [12.41] years vs 45.58 [16.02] years), as was body mass index (BMI; calculated as weight in kilograms divided by height in meters squared) (mean [SD], 28.5 [8.9] vs 24.1 [4.2]). MHT use varied substantially across studies, and follow-up ranged from 4.8 to 36.0 years (median, 16.0 years); 12 studies (33.3%) did not report the duration of follow-up^6,8,42,43,44,45,46,47,49,50,52,54^ (eTable 12 in Supplement 1).

### Risk of Bias in the Studies

Overall study quality was moderate to high (eTables 1-3 in Supplement 1). Median quality score was 9 (range, 7-9) for cohort and case-control studies^7,9,15,16,17,18,19,20,30,31,32,33,34,35,36,37,38,39,40,41,42,43,54^ and 7 (range, 5-9) for cross-sectional studies.^8,14,44,45,46,47,48,49,50,51^ RCTs showed a low overall risk of bias.^52,53^ Adjustment for confounders varied, with 15 studies (41.7%) showing low robustness (≤4 variables)^8,16,18,37,41,44,45,46,47,48,49,50,51,53,54^; 11 (30.6%), moderate robustness (5-6 variables)^6,7,14,17,20,30,33,34,38,39,42^; and 10 (27.8%), high robustness (≥7 variables).^9,15,19,31,32,35,36,40,43,52^ Most commonly, adjustments were for age, BMI, smoking status, blood pressure, diabetes, lipid levels, MHT use, and socioeconomic factors. The study by Zhu et al^6^ was not assessed as a separate study in the risk-of-bias evaluation because it reported a pooled analysis of individual studies; risk of bias was instead assessed at the level of the contributing studies.

### Qualitative Findings

Qualitative findings are presented in eTables 4 through 8 and the eResults in Supplement 1. Excess risk of cardiovascular morbidity and mortality after hysterectomy with BSO was supported by subclinical vascular measures and large-cohort incidence data, particularly before age 50 years and in *BRCA* carriers, whereas no consistent association was found for chemotherapy- and treatment-induced POI. Stroke and metabolic syndrome findings were mixed, congestive heart failure risk was inversely associated with age at menopause, and both mortality outcomes (cardiovascular and all-cause) were generally null overall but were elevated with younger age at surgery. MHT findings were heterogenous and largely null except at the youngest ages (eTable 13 in Supplement 1). Baseline cardiovascular risk stratification of participants across all included studies and a qualitative summary of cardiovascular and mortality outcomes following surgical menopause, stratified by baseline cardiovascular risk, are given in eTables 14 and 15 in Supplement 1.

### Quantitative Findings

#### Cardiovascular Morbidity

Composite cardiovascular events were reported by 7 cohort studies (19.4%).^6,19,20,33,34,40,41^ Under the random-effects model, iatrogenic menopause was associated with a higher risk of these events (pooled HR, 1.25; 95% CI, 1.12-1.39; *I*^2^ = 86%) (Figure 2A). The estimate was robust across leave-1-out analyses, and the heterogeneity was attributable to the study by Farland et al,^40^ the omission of which reduced *I*^2^ to 0% (eTable 16 in Supplement 1). Comparator type did not account for heterogeneity, and heterogeneity persisted within the no-surgery referent group (eFigure 1 in Supplement 1).

**Figure 2.**
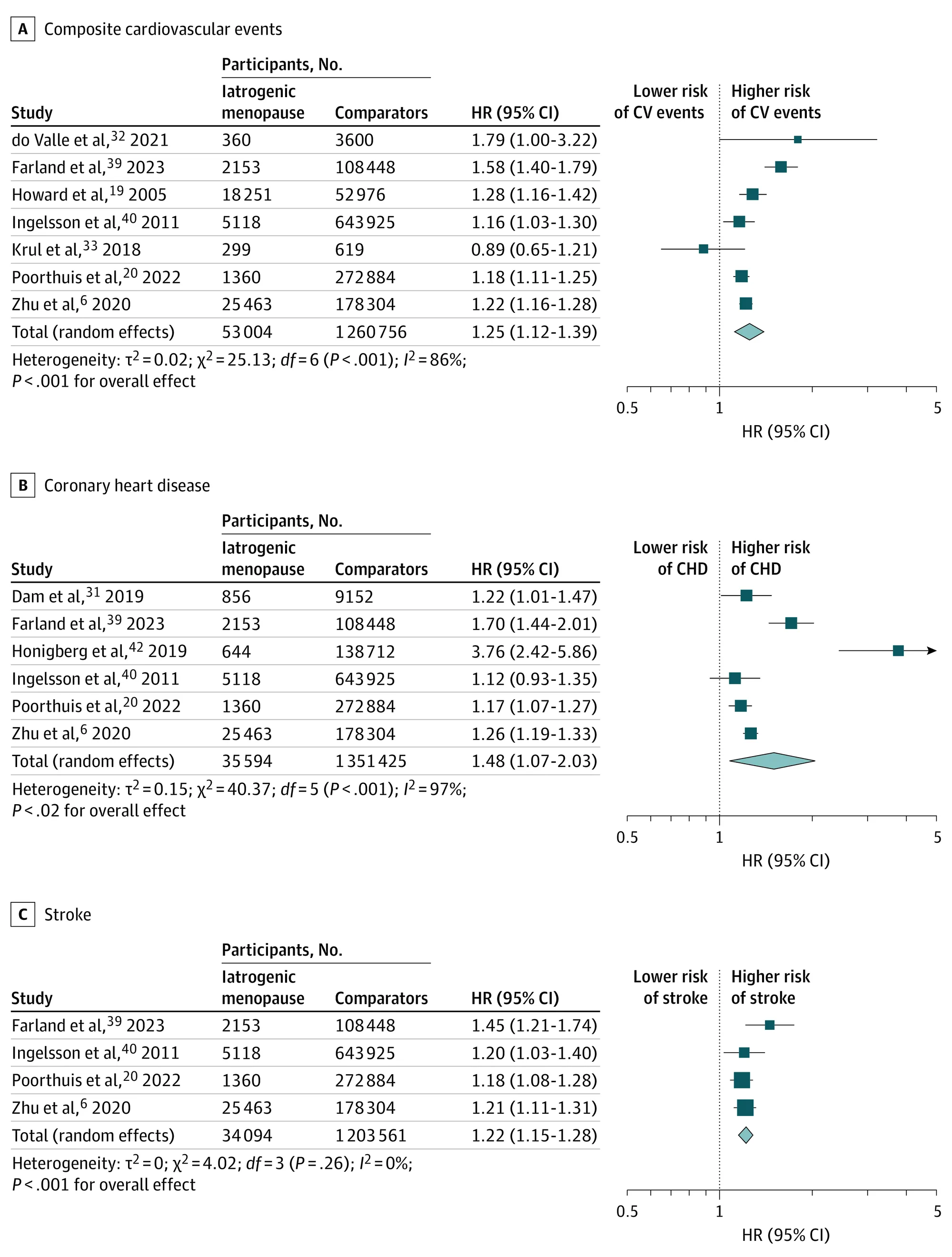
Forest Plots of Hazard Ratios (HRs) for Cardiovascular Morbidity Among Women With Iatrogenic Menopause vs Comparator Groups B, Honigberg et al^43^ contributed the estimate for menopause before age 40 years.

Coronary heart disease was reported by 6 cohort studies (16.7%).^6,20,32,40,41,43^ The pooled HR was 1.48 (95% CI, 1.07-2.03; *I*^2^ = 97%) (Figure 2B) and decreased to 1.45 (95% CI, 0.97-2.15) with omission of the study by Farland et al.^40^ Omission of the study by Honigberg et al^43^ reduced *I*^2^ to 85% (eTable 16 in Supplement 1). The test for subgroup differences by comparator type was significant (χ^2^ = 23.28; *df* = 2; *P* < .001), but the study by Honigberg et al^43^ formed its own subgroup and was distinguished from the other cohorts by restricting exposure to menopause before age 40 years rather than by its referent population (eFigure 2 in Supplement 1).

Stroke was reported by 4 studies (11.1%).^6,20,40,41^ The pooled HR was 1.22 (95% CI, 1.15-1.28), with no detectable heterogeneity (Figure 2C). The pooled estimate was robust in leave-1-out analysis (eTable 16 in Supplement 1). The stratification by comparator type showed no subgroup difference (eFigure 3 in Supplement 1).

#### Cardiovascular Mortality

Cardiovascular mortality was reported by independent cohorts in 6 studies (16.7%) contributing 7 estimates.^7,15,16,31,35,36,39^ Two of these estimates, from Rivera et al^35^ and Rocca et al,^36^ were separate reports of the same Olmsted County, Minnesota, population (eTable 17 in Supplement 1). Under the random-effects model, no overall excess cardiovascular mortality was found (pooled HR, 1.03; 95% CI, 0.94-1.13) (Figure 3A). Leave-1-out analysis did not change the results (eTable 16 in Supplement 1).

**Figure 3.**
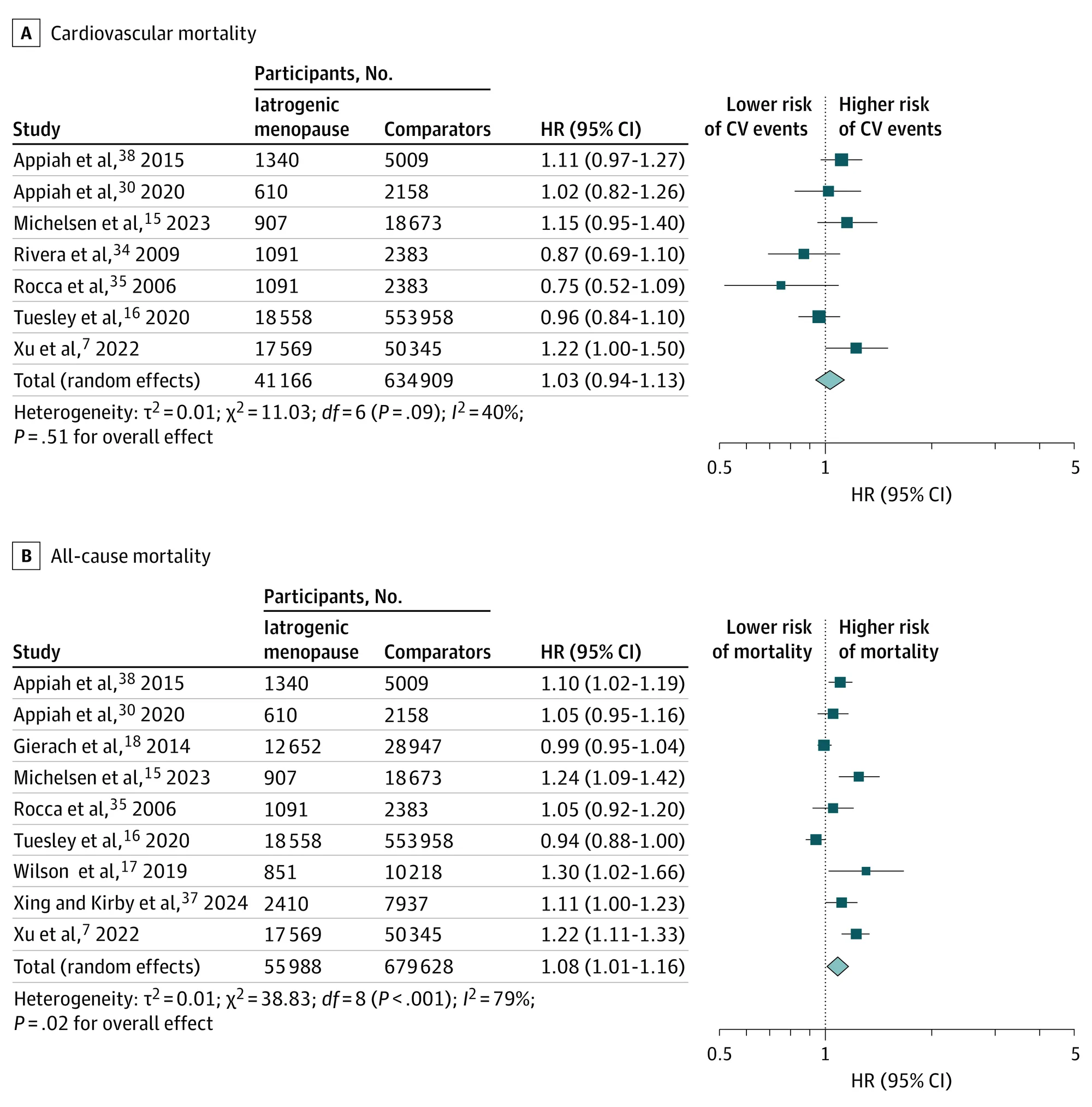
Forest Plots of Hazard Ratios (HRs) for Cardiovascular and All-Cause Mortality Among Women With Iatrogenic Menopause vs Comparator Groups A, Rivera et al^7^ and Rocca et al^6^ are separate reports of the same Olmsted County, Minnesota, population; thus, the 1091 women with iatrogenic menopause and 2383 comparators are included twice in the column totals. The deduplicated counts are 40 075 women with iatrogenic menopause and 632 526 comparators (eTable 9 in Supplement 1). Fixed-effects and leave-1-out analyses are given in eTables 9 and 16 in Supplement 1. A sensitivity analysis retaining 1 report at a time is given in eTable 17 in Supplement 1. B, No source population is duplicated; the Olmsted County cohort was contributed by Rocca et al^6^ alone.

#### All-Cause Mortality

Cohorts in 9 studies (25.0%)^7,15,16,17,18,31,36,38,39^ contributed to the pooled analysis for all-cause mortality. Random-effects estimates indicated a modest excess in risk (pooled HR, 1.08; 95% CI, 1.01-1.16; *I*^2^ = 79%) (Figure 3B). This estimate was sensitive to the omission of individual cohorts: across the 9 leave-1-out analyses, the pooled HRs ranged from 1.07 (95% CI, 1.00-1.14) to 1.11 (95% CI, 1.04-1.18), but the estimate was no longer significant after omission of either Xu et al^7^ or Michelsen et al^15^ (eTable 16 in Supplement 1).

#### Age at Iatrogenic Menopause

When cardiovascular mortality was analyzed by age at iatrogenic menopause vs women without oophorectomy (Figure 4A), the pooled HR was 1.20 (95% CI, 1.04-1.37; *I*^2^ = 0%) below age 45 years and 0.95 (95% CI, 0.81-1.11; *I*^2^ = 61%) at or above age 45 years. Both subgroup estimates were stable after omission of individual age bands (eTables 18 and 19 in Supplement 1).

**Figure 4.**
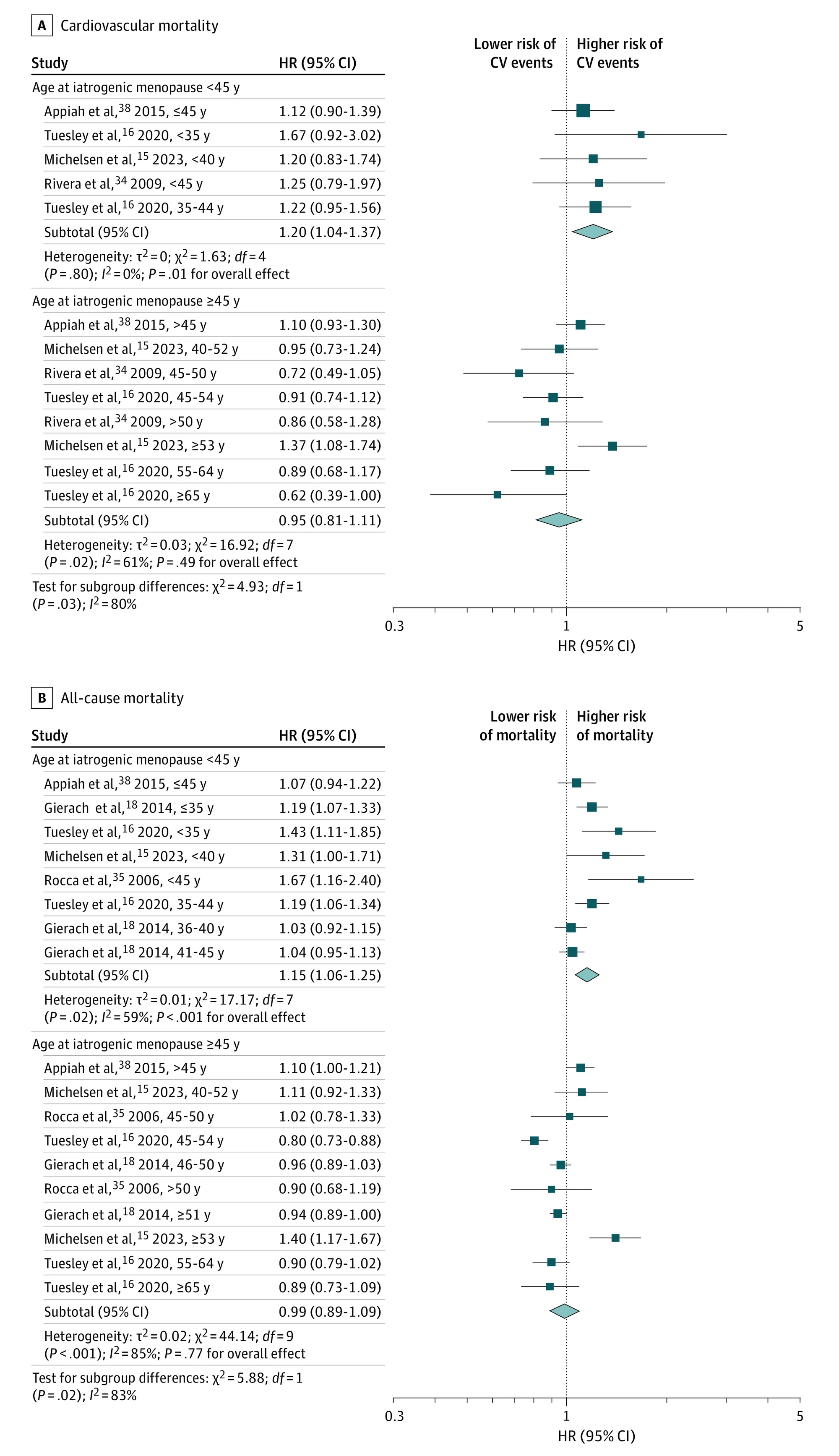
Forest Plots of Hazard Ratios (HRs) for Cardiovascular and All-Cause Mortality Stratified by Age at Iatrogenic Menopause The subgroup estimates are shown for description; the formal age contrast, with cohorts as the unit of inference, is a ratio of HRs of 0.77 (95% CI, 0.58-1.04) for cardiovascular mortality and 0.82 (95% CI, 0.68-0.98) for all-cause mortality (eTable 20 in Supplement 1). No overall total is shown for the random-effects model because the 2 subgroups share source cohorts. Both panels are restricted to cohorts whose referent group is defined by the absence of surgery. The single band spanning the cutoff (Michelsen et al^8^; age, 40-52 years) was assigned to the older group on the basis of its midpoint. The test for subgroup differences treats age bands as independent and is anticonservative; the formal age contrast, with cohorts as the unit of inference, is reported in eTable 20 in Supplement 1.

When all-cause mortality was analyzed by age at iatrogenic menopause vs women without oophorectomy, the pooled HR was 1.15 (95% CI, 1.06-1.25; *I*^2^ = 59%) among women in whom menopause occurred before age 45 years and 0.99 (95% CI, 0.89-1.09; *I*^2^ = 85%) among women in whom menopause occurred at or after age 45 years (Figure 4B). Both subgroup estimates were robust to leave-1-out omission of any single age band (eTable 18 in Supplement 1).

#### Meta-Regression of Age at Iatrogenic Menopause vs End Points

Age at iatrogenic menopause was examined as a continuous moderator in random-effects meta-regression. With cohorts as the unit of inference, the pooled HR per increasing 5-year age increment for all-cause mortality was 0.94 (95% CI, 0.86-1.02) using 25 estimates from 7 studies (19.4%)^7,15,16,18,36,38,39^ and for cardiovascular mortality was 0.92 (95% CI, 0.79-1.08) using 18 estimates from 5 studies (13.4%).^7,15,16,35,39^ There was no agreement on the gradient among studies, and between-cohort heterogeneity in the fitted slope was significant for both outcomes (*Q* = 18.3; *df* = 6; *P* = .006 for all-cause mortality and *Q* = 11.0; *df* = 4; *P* = .03 for cardiovascular mortality). The point estimate was therefore stable, while the inference was not (eTable 20 in Supplement 1).

Further exploratory post hoc analysis evaluated the association of age at iatrogenic menopause with the pooled end point of cardiovascular morbidity and mortality (Figure 5 and eTable 21 in Supplement 1). This was plausible because each outcome-specific model was based on 3 to 7 cohorts and no cohort contributed age bands to both a morbidity and a mortality end point. When taking 1 end point per source population so that no population contributed twice and the 2 reports of the Olmsted County cohort^35,36^ were combined, the pooled HR per increasing 5-year age increment was 0.89 (95% CI, 0.83-0.97) using 37 estimates from 11 independent populations (30.6%).^6,7,15,16,18,20,35,36,38,39,40,41^ Unlike the outcome-specific models, this estimate was stable after omission of any single population (HR range, 0.88 [95% CI, 0.81-0.95; *P* = .005] to 0.91 [95% CI, 0.85-0.99; *P* = .03]) and with every pairing of a morbidity with a mortality end point (HR range, 0.87 [95% CI, 0.81-0.94; *P* = .003] to 0.90 [95% CI, 0.83-0.98; *P* = .02]). Two distribution-free analyses confirmed the direction of the gradient (Kendall rank correlation, −0.78 across 6 populations with ≥3 age bands [*P* < .001]; sign-flip permutation test on 11 population slopes [*P* = .007]). Populations had agreement on direction but not magnitude (*Q* = 138; *df* = 10; *P* < .001), indicating that risk was consistently lower with later menopause without these data quantifying that difference for an individual woman (Figure 5).

**Figure 5.**
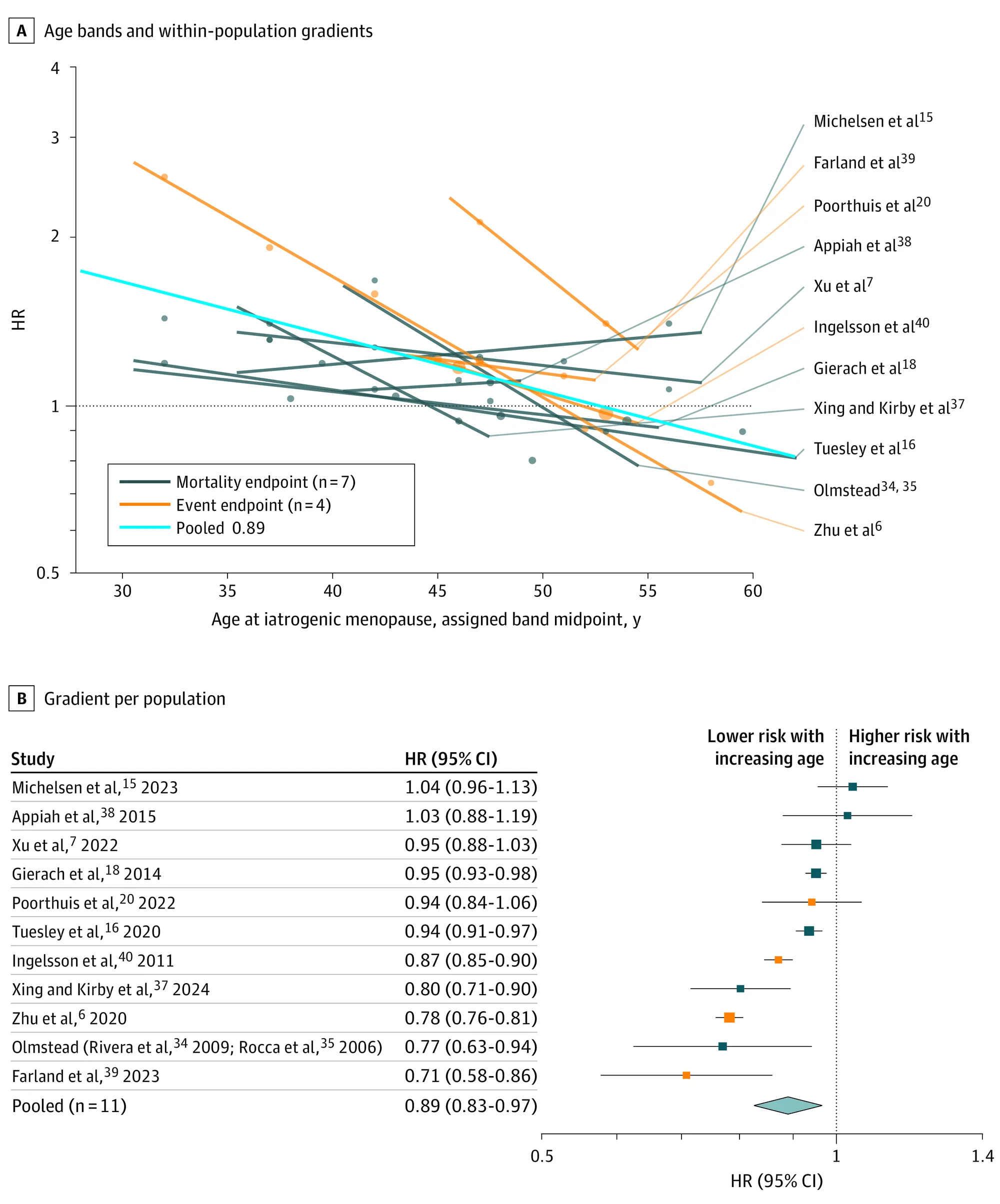
Line Graph of Age at Iatrogenic Menopause Gradient Pooled Across Cardiovascular End Points and Forest Plot of the Composite of Cardiovascular Morbidity and Mortality A, Each disjoint age-band estimate from the contributing cohorts is plotted against the midpoint of its age band on a logarithmic hazard ratio (HR) scale. Dots are proportional to the inverse-variance weight; colored lines are within-population gradients and the black line is the population-averaged gradient. Shaded bands indicate cohort-clustered 95% CIs. Bands spanning the 45-year threshold are included at their midpoints; thus, no assignment rule is required.

#### Analysis by Comparator Type

Stratification by comparator type was undertaken post hoc and is reported as exploratory; forest plots, heterogeneity statistics, and referent definitions are given in eFigures 1 through 5, the eResults, and eTable 11 in Supplement 1. For all-cause mortality, the pooled estimate of risk was higher compared with a natural-menopause referent (HR, 1.16; 95% CI, 1.06-1.27) but not compared with a referent of no oophorectomy or pelvic surgery (HR, 1.06; 95% CI, 0.99-1.14), with no difference between strata (eFigure 5 in Supplement 1). For the remaining outcomes, at least 1 referent group was formed by a single cohort and the comparisons were underpowered; none showed a difference by comparator.

#### Exploratory Analysis for MHT

MHT was examined as a prespecified exploratory moderator of all-cause mortality. Of the 9 contributing studies,^7,15,16,17,18,31,36,38,39^ prevalence of MHT use by exposure group was reported by 6,^15,17,18,31,38,39^ and neither the between-group difference in MHT prevalence nor the prevalence in the exposed group alone supported a robust association. Too few cardiovascular mortality estimates reported prevalence of MHT use to fit a model, and within-cohort estimates by MHT stratum, available for 3 cohorts,^15,17,18^ did not reach significance. Because MHT prescribing is frequently determined by age at the procedure, these analyses could not separate MHT use from the age gradient (eTables 22 and 23 in Supplement 1).

#### Level of Evidence

All studies contributing to cardiovascular morbidity, cardiovascular mortality, and all-cause mortality were nonrandomized, so every outcome began at low certainty (eTable 10 in Supplement 1). Certainty was moderate for stroke (pooled HR, 1.22; 95% CI, 1.15-1.28), for which no between-study heterogeneity was detected; low for composite cardiovascular events; and very low for coronary heart disease and both mortality outcomes. Ratings were raised when plausible residual confounding would be expected to attenuate rather than generate the association.

## Discussion

In this study, iatrogenic menopause was associated with higher risk of cardiovascular morbidity, most notably from stroke (PHR, 1.22; 95% CI, 1.15-1.28), but not with overall excess of cardiovascular mortality and only with a modest increase for all-cause mortality; the excess mortality was concentrated below age 45 years for both the mortality outcomes. Risk was lower the later iatrogenic menopause occurred, but that gradient was present only for the composite outcome of morbidity and mortality (PHR, 0.89 [95% CI, 0.83-0.97] per 5-year age increase), not in either outcome alone; there was substantial disagreement in its magnitude between populations, but the direction was reliable.

Despite the observed associations, their interpretation requires caution given the substantial heterogeneity across studies. The most consistent morbidity association was found for stroke (PHR, 1.22; 95% CI, 1.15-1.28), supporting long-term cardiovascular surveillance after iatrogenic menopause. For all-cause mortality, heterogeneity was substantial and the association was sensitive to the omission of individual cohorts, whereas the estimate for cardiovascular mortality remained stable. Earlier syntheses differed mainly for methodologic rather than statistical reasons: comparator groups varied in whether hysterectomy with ovarian conservation was included, and there was broad age categorization and limited confounder adjustment.^6^ Clinical differences among comparator groups and rates of MHT use likely contributed to heterogeneity, although no significant differences between comparator strata were identified.^6,55,56^

Our findings showed a direction of risk in line with evidence retrieved from previous meta-analyses.^13,57^ Specifically, the 1.22-times higher risk for stroke observed in our study aligns with the risk reported by Hassan et al^13^ and Attachaipanich et al^57^ despite differences in comparator definitions and pooling methods. To our knowledge, this is the first study to report a direction of age-related gradient in excess cardiovascular morbidity and mortality after iatrogenic menopause. A similar age gradient between cardiovascular risk or dementia has previously been described among women experiencing spontaneous premature menopause.^58,59^

The concentration of excess risk before age 45 years is biologically plausible: iatrogenic menopause at a younger age abruptly removes ovarian steroid support for vascular, metabolic, and inflammatory function^60,61,62^ before physiologic adaptation to declining ovarian function has occurred, whereas surgery closer to the expected age of natural menopause affects an already partially adapted physiology. This pattern may plausibly reflect the abrupt reduction in estrogen and related ovarian steroids involved in regulating vascular tone, lipid and glucose homeostasis, mitochondrial energetics, endothelial function, and inflammatory pathways.^60,61,62^ Current clinical guidance primarily emphasizes the oncologic and gynecologic indications for oophorectomy while offering limited quantitative guidance on long-term cardiometabolic risk.^63,64,65^ If confirmed by future longitudinal studies, this study’s findings could potentially support more individualized surgical counseling by providing age-specific, risk-stratified estimates.

### Limitations

Limitations of this study include residual confounding, as minimally adjusted estimates were prioritized to avoid overadjustment for mediators; heterogeneity in definitions, comparator groups, and baseline risk; reliance on study-level rather than individual participant data; and limited precision in subgroup and meta-regression analyses, which is governed by the number of contributing cohorts rather than the number of events. Small-study effects could not be assessed formally. Generalizability is also restricted: the evidence was derived predominantly from high-income cohorts, and race and ethnicity were reported by only 18 of the 36 included studies, which described populations that were predominantly White or of European ancestry, with a single Chinese cohort.

## Conclusions

In this systematic review and meta-analysis, cardiovascular and mortality risks after iatrogenic menopause were associated with age at iatrogenic menopause. Across the composite of cardiovascular and mortality end points, older age at surgery was associated with progressively lower risk and stroke was the most consistently increased individual outcome. These findings could be used to inform counseling and long-term follow-up rather than to set an age criterion for surgery. The available data on MHT after iatrogenic menopause were heterogenous and sparsely reported. This review therefore cannot support any conclusion about MHT as a risk-mitigation strategy after iatrogenic menopause, and its role remains a priority evidence gap. Further studies should refine individualized risk prediction.

## Notes

Supplement 1.
eMethods

eResults
**eTable 1.** Newcastle-Ottawa Scale Quality Assessment for Cohort and Case-Control Studies **eTable 2.** Newcastle-Ottawa Scale Quality Assessment for Cross-Sectional Studies **eTable 3.** Cochrane Risk of Bias 2.0 Assessment for Randomized Clinical Trials **eTable 4.** Studies Reporting on Absolute, Relative, and Incident Risk for Cardiovascular Disease and Coronary Heart Disease **eTable 5.** Studies Reporting on Absolute, Relative, and Incident Risk for Stroke and Metabolic Syndrome Morbidity **eTable 6.** Studies Reporting on Absolute, Relative, and Incident Risk for Congestive Heart Failure **eTable 7.** Studies Reporting on Absolute, Relative, and Incident Risk for Cardiovascular Disease Mortality **eTable 8.** Studies Reporting on Absolute, Relative, and Incident Risk for All-Cause Mortality **eTable 9.** Fixed-Effect Sensitivity Analysis of the Pooled Estimates Shown in Figure 2 and Figure 3 **eTable 10.** GRADE Certainty of Evidence for Each Pooled Outcome **eTable 11.** Descriptive Characteristics of the Included Studies **eTable 12.** Main Anthropometric Characteristics of Participants and Menopause-Related Characteristics in the Included Studies **eTable 13.** Studies Reporting on the Role of Menopausal Hormone Therapy on the Absolute, Relative, and Incident Risk for Cardiovascular Disease, Coronary Heart Disease, and Stroke Morbidity, and for Cardiovascular and All-Cause Mortality **eTable 14.** Cardiovascular Risk Stratification of Participants at Baseline for All Studies **eTable 15.** Qualitative Summary of Cardiovascular and Mortality Outcomes After Surgical Menopause, Stratified by Baseline Cardiovascular Risk **eTable 16.** Leave-One-Out Sensitivity Analysis of the Pooled Estimates, by Outcome **eTable 17.** Sensitivity Analysis for the Overlapping Reports of the Olmsted County Cohort, Cardiovascular Mortality **eTable 18.** Leave-One-Out Sensitivity Analysis of the Age-Stratified Mortality Analyses **eTable 19.** Age Bands as Reported by Each Contributing Cohort and the Age Interval Across Which Each Contrast Is Measured **eTable 20.** Age at Iatrogenic Menopause as a Moderator of Mortality and Cardiovascular Outcomes, by Analysis Set and Unit of Inference **eTable 21.** Age at Iatrogenic Menopause as a Continuous Moderator, Pooled Across Cardiovascular Morbidity and Mortality End Points, With Independent Source Populations as the Unit of Inference **eTable 22.** Within-Cohort Modification of the Association by Menopausal Hormone Therapy Stratum **eTable 23.** Exploratory Cohort-Level Meta-Regression of All-Cause Mortality on Menopausal Hormone Therapy Prevalence **eFigure 1.** Composite Cardiovascular Events, Stratified by Comparator Type **eFigure 2.** Coronary Heart Disease, Stratified by Comparator Type **eFigure 3.** Stroke, Stratified by Comparator Type **eFigure 4.** Cardiovascular Mortality, Stratified by Comparator Type **eFigure 5.** All-Cause Mortality, Stratified by Comparator Type
eReferences

eAppendix

Supplement 2.
Data Sharing Statement
