# Supplementary material for: Age at Iatrogenic Menopause and Cardiovascular Morbidity and Mortality: A Systematic Review and Meta-Analysis
Source: JAMA Netw Open. 2026 Oct 2;9(10):e2637545. doi: 10.1001/jamanetworkopen.2026.37545 (PMC13633536; doi:10.1001/jamanetworkopen.2026.37545)
Supplement: Supplementary file 1 [file jamanetwopen-e2637545-s001.pdf]

Supplementary Online Content

Armeni E, Ozcivit Erkan IB, Kempegowda P. Age at iatrogenic menopause and cardiovascular morbidity and mortality: a systematic review and meta-analysis. *JAMA Netw Open*. 2026;9(10):e2637545. doi:10.1001/jamanetworkopen.2026.37545

eMethods
eResults
eTable 1. Newcastle-Ottawa Scale Quality Assessment for Cohort and Case-Control Studies
eTable 2. Newcastle-Ottawa Scale Quality Assessment for Cross-Sectional Studies
eTable 3. Cochrane Risk of Bias 2.0 Assessment for Randomized Clinical Trials
eTable 4. Studies Reporting on Absolute, Relative, and Incident Risk for Cardiovascular Disease and Coronary Heart Disease
eTable 5. Studies Reporting on Absolute, Relative, and Incident Risk for Stroke and Metabolic Syndrome Morbidity
eTable 6. Studies Reporting on Absolute, Relative, and Incident Risk for Congestive Heart Failure
eTable 7. Studies Reporting on Absolute, Relative, and Incident Risk for Cardiovascular Disease Mortality
eTable 8. Studies Reporting on Absolute, Relative, and Incident Risk for All-Cause Mortality
eTable 9. Fixed-Effect Sensitivity Analysis of the Pooled Estimates Shown in Figure 2 and Figure 3
eTable 10. GRADE Certainty of Evidence for Each Pooled Outcome
eTable 11. Descriptive Characteristics of the Included Studies
eTable 12. Main Anthropometric Characteristics of Participants and Menopause-Related Characteristics in the Included Studies
eTable 13. Studies Reporting on the Role of Menopausal Hormone Therapy on the Absolute, Relative, and Incident Risk for Cardiovascular Disease, Coronary Heart Disease, and Stroke Morbidity, and for Cardiovascular and All-Cause Mortality
eTable 14. Cardiovascular Risk Stratification of Participants at Baseline for All Studies
eTable 15. Qualitative Summary of Cardiovascular and Mortality Outcomes After Surgical Menopause, Stratified by Baseline Cardiovascular Risk
eTable 16. Leave-One-Out Sensitivity Analysis of the Pooled Estimates, by Outcome
eTable 17. Sensitivity Analysis for the Overlapping Reports of the Olmsted County Cohort, Cardiovascular Mortality
eTable 18. Leave-One-Out Sensitivity Analysis of the Age-Stratified Mortality Analyses
eTable 19. Age Bands as Reported by Each Contributing Cohort and the Age Interval Across Which Each Contrast Is Measured
eTable 20. Age at Iatrogenic Menopause as a Moderator of Mortality and Cardiovascular Outcomes, by Analysis Set and Unit of Inference
eTable 21. Age at Iatrogenic Menopause as a Continuous Moderator, Pooled Across Cardiovascular Morbidity and Mortality End Points, With Independent Source Populations as the Unit of Inference

© 2026 Armeni E. *JAMA Network Open*. This is an open access article distributed under the terms of the CC-BY-NC-ND License, which does not permit alteration or commercial use, including those for text and data mining, AI training, and similar technologies.

eTable 22. Within-Cohort Modification of the Association by Menopausal Hormone Therapy Stratum
eTable 23. Exploratory Cohort-Level Meta-Regression of All-Cause Mortality on Menopausal Hormone Therapy Prevalence
eFigure 1. Composite Cardiovascular Events, Stratified by Comparator Type
eFigure 2. Coronary Heart Disease, Stratified by Comparator Type
eFigure 3. Stroke, Stratified by Comparator Type
eFigure 4. Cardiovascular Mortality, Stratified by Comparator Type
eFigure 5. All-Cause Mortality, Stratified by Comparator Type
eReferences
eAppendix

This supplementary material has been provided by the authors to give readers additional information about their work.

© 2026 Armeni E. *JAMA Network Open*. This is an open access article distributed under the terms of the CC-BY-NC-ND License, which does not permit alteration or commercial use, including those for text and data mining, AI training, and similar technologies.

eMethods

Scope of this supplement

This supplement expands the 3 elements of the Methods that the main article defers to it: the operational thresholds applied in the risk-of-bias assessment, the handling of comparators together with within-study combination and the referent-purity restrictions, and the full list of sensitivity analyses. It also sets out in full the models used to examine age at iatrogenic menopause as a moderator, including the derivation of the cohort-level ratio of hazard ratios that is the formal age contrast reported in the main article. The sections below follow the order in which the main Methods raises each point. No numerical result is presented here except where derivation is being documented; the results of every analysis described in this supplement are reported in the eResults, in **eTables 1-23** and in **eFigures 1-5**.

Search strategy and selection criteria

The protocol for this systematic review and meta-analysis was prospectively registered in PROSPERO (registration number CRD420251033146, registered in 2025), and the review was conducted and reported in accordance with the PRISMA 2020 statement. The eligibility criteria, the definition of iatrogenic menopause, the admissible comparator groups, the eligible outcomes and the exclusion criteria are presented in full, together with the database-specific search strategies and the list of reports not retrieved and reports excluded at full-text assessment, with reasons, in the **eAppendix** at the end of this Supplement. In practice, nearly all included studies evaluated surgical menopause; only 3 included medically induced menopause.

All databases were searched in duplicate on 15 March 2026; the date range and the categories of search terms are given in the Methods of the main article. The 2004 start date excludes publications predating the early termination of the Women's Health Initiative hormone therapy trials, after which prescribing practice and the reporting of cardiovascular outcomes in this population changed substantially. Reference lists of eligible studies and of relevant reviews were screened to identify additional records. All records were screened at the title and abstract stage and at full text in Covidence by 2 reviewers working independently.

Data extraction

The variables extracted and the rule of selecting the minimally adjusted model, where a study reported more than 1 analytical model, are stated in the Methods of the main article. Two points of application are recorded here. First, the minimally adjusted rule was applied outcome by outcome, so that where a study reported several models for 1 outcome and a single model for another, the minimally adjusted estimate was taken for the first and the only available estimate for the second; the adjustment set may therefore differ between outcomes within the same cohort. This arises once in the present review, for Tuesley et al,[25] 2020, which publishes an age-adjusted and a fully adjusted model for all-cause mortality but only the fully adjusted model for cardiovascular mortality; the age-adjusted estimates were therefore taken for all-cause mortality and the fully adjusted estimates for cardiovascular mortality. The model from which each cohort's estimates were drawn is recorded in the data extraction form. Second, for studies reporting age at menopause in categories, band midpoints were assigned for quantitative analyses; the midpoint assigned to every band is listed in **eTable 19**. Where the required quantity could not be reliably derived, the study was excluded from the specific analysis concerned but retained in the review. The rules used to combine estimates within a study, and the treatment of mutually exclusive, cumulative and nested age categories, are set out below.

Risk of bias

Risk of bias was evaluated independently by the 2 reviewers using design-specific instruments: the Cochrane RoB 2.0 tool for randomized trials and the Newcastle–Ottawa Scale (NOS) for cohort, case–control and cross-sectional studies. Each study was rated across the selection, comparability and outcome or exposure domains. The main article reports these ratings as low, moderate or high; the thresholds behind those 3 categories are as follows. A study was rated at low risk of bias if all domains were low risk under RoB 2.0, or if it scored 7 to 9 stars overall on the NOS with at least 1 star in selection, 1 to 2 stars in comparability and 2 to 3 stars in outcome or exposure. A study was rated at moderate risk if at least 1 domain raised some concerns under RoB 2.0 but no domain was at high risk, or if it scored 4 to 6 stars on the NOS. A study was rated at high risk if at least 1 domain was at high risk under RoB 2.0, or if several domains raised concerns sufficient to lower confidence substantially, or if it scored 0 to 3 stars on the NOS. Domain-level judgements for every study are given in **eTables 1-3**.

Effect measures and pooling

All eligible effect estimates are presented in the measure originally reported, including hazard ratios (HRs), risk ratios, odds ratios, incidence or mortality rate ratios and, where available, event counts and group sizes (**eTables**

© 2026 Armeni E. *JAMA Network Open*. This is an open access article distributed under the terms of the CC-BY-NC-ND License, which does not permit alteration or commercial use, including those for text and data mining, AI training, and similar technologies.

4-8). The stratification of that narrative synthesis by baseline cardiovascular risk profile, surgical indication, design and adjustment strategy is described in the main article.

Quantitative pooling and meta-regression were restricted to HRs, because these represent time-to-event outcomes and are not interchangeable with ratio measures derived from cumulative incidence or prevalence. Studies reporting only other effect measures, and cross-sectional studies in which the temporality of the outcome was unclear, were tabulated but contributed to the narrative synthesis alone. This restriction, rather than any judgement of study quality, accounts for the difference between the 36 studies included in the review and the smaller number of cohorts contributing to each pooled estimate.

Reported HRs and their 95% CIs were transformed to log HRs, with standard errors calculated as the difference between the natural logarithms of the upper and lower confidence limits divided by 3.92, pooled by generic inverse variance and exponentiated for presentation. Where at least 2 studies reported comparable exposures and outcomes, random-effects models were fitted with restricted maximum-likelihood (REML) estimation of the between-study variance and Wald 95% CIs. Random-effects estimates remained primary for every outcome irrespective of the observed heterogeneity, and fixed-effect inverse-variance estimates were calculated for comparison only (**eTable 9**). Heterogeneity was interpreted from the between-study variance, the direction and dispersion of the contributing estimates and Cochran Q, rather than from I^2 alone, because I^2 is a proportion rather than a magnitude and is unstable when few estimates contribute.

Within-study combination of subgroup estimates

Where a study reported eligible estimates only for mutually exclusive age bands or other compatible subgroups, and no overall estimate for the exposure of interest, a single study-level estimate was required before that cohort could enter a pooled analysis. In these cases the subgroup estimates were combined within the study by fixed-effect inverse-variance meta-analysis on the log scale, so that each cohort contributed 1 estimate to each primary pooled analysis. Fixed-effect combination was used because the subgroups of a single study share a population, a design and an analytical model. It was applied only where the subgroup estimates were mutually compatible, that is, mutually exclusive in the women they contain and referenced to the same comparator group. Three studies were entered in this way: Ingelsson et al,[1] 2010, Xing et al,[2] 2024 and Xu et al,[3] 2022.

Where age bands shared a comparator group, that comparator was counted once in the comparator total. The resulting standard error is conservative, because the positive correlation induced between bands by the shared referent was not explicitly modelled. Mutually incompatible estimates were not combined; such studies were entered by age band or omitted from the pooled analysis.

Cumulative and nested age categories were not combined, because they contain overlapping participants. Where a study reported successive inclusive thresholds, so that each group contained all women below the specified cutoff, only 1 threshold from that study was admitted to any analysis, and cumulative series contributed neither to pooled analyses nor to meta-regression.

Subgroups defined by a factorial covariate against a shared referent required additional handling. In such studies the published cell for the exposed group within the covariate-positive stratum is a joint estimate of both exposures against the doubly unexposed referent, so that naive combination of the printed cells would overstate the association with iatrogenic menopause. In Appiah et al,[4] 2020 the cell jointly representing iatrogenic menopause and family history of premature myocardial infarction was therefore divided by the corresponding family-history-only cell before within-study combination, isolating the association with iatrogenic menopause. This produced an HR of 1.02 (95% CI, 0.82-1.26) for cardiovascular mortality, based on 610 exposed and 2158 comparator women, which is the value entered in **Figure 3A**; the corresponding all-cause value is 1.05 (95% CI, 0.95-1.16). Appiah et al,[5] 2015 reported factorial contrasts for oophorectomy and diabetes against a common reference group; because only the subgroup without diabetes provided an unconfounded contrast for oophorectomy, that subgroup alone was included and labeled accordingly.

Rocca et al,[6] 2006 and Rivera et al,[7] 2009 report substantially overlapping participants from the same Olmsted County population, so that this cohort is counted twice in the primary cardiovascular mortality analysis. Their joint inclusion is identified in the legend to **Figure 3** and examined in sensitivity analysis (**eTable 17**).

Comparator handling and referent-purity restrictions

Comparator definition was addressed in 3 stages. Primary analyses admitted all eligible comparator groups. Estimates were then stratified according to whether the referent comprised women with natural menopause or women who had not undergone oophorectomy or pelvic surgery, because these 2 referent definitions do not estimate the same quantity: the first contrasts 2 routes to the same endocrine state, whereas the second contrasts the endocrine state itself against its absence (**eFigures 1-5**). Third, for the age-stratified mortality analyses, a

© 2026 Armeni E. *JAMA Network Open*. This is an open access article distributed under the terms of the CC-BY-NC-ND License, which does not permit alteration or commercial use, including those for text and data mining, AI training, and similar technologies.

graded referent-purity restriction was applied, described in the next paragraph. Stratification by comparator type and the graded restriction were both post hoc and are reported as exploratory. Several contributing cohorts describe their comparators as age-matched, meaning matched on attained age at study entry or at analysis; individual referents fulfilled elements of the ideal comparison, unoperated women premenopausal at the exposed women's age at surgery, but no included estimate fulfilled all of them. The 2 cohorts that compare each age band with a referent group matched to that band are identified in the eResults, under "Age matching and exposure definition".

The graded referent-purity restriction proceeds in 4 steps of increasing stringency, each removing cohorts whose comparator group could contain women whose ovarian function had also ceased or been altered. The first step is the primary analysis set, comprising all contributing cohorts. The second retains only cohorts whose referent is defined by the absence of surgery, and so excludes the cohorts using a natural-menopause referent. The third additionally excludes Appiah et al,[5] 2015, whose comparator group is defined as the absence of bilateral salpingo-oophorectomy and therefore includes women who underwent hysterectomy with ovarian conservation. The fourth and strictest retains only cohorts in which hysterectomy alone is handled as a separate exposure group. Because each step removes a cohort, and inference rests on the number of cohorts rather than the number of women, the restricted analyses are less precise by construction; they are reported to show the direction in which comparator impurity moves the estimate, not to provide a more reliable estimate. Extended analysis sets, formed by adding the natural-menopause-referent cohorts back one at a time, are reported alongside them (**eTable 20**).

Age at iatrogenic menopause as a moderator

Mortality outcomes were stratified at the conventional clinical cutoff of 45 years (**Figures 4A and 4B**). This threshold was prespecified on clinical grounds and was not derived from the data. Both panels of Figure 4 are restricted to cohorts whose referent group is defined by the absence of surgery, whereas the cohort-level contrasts and the continuous gradients shown in Figure 5 are derived from the full analysis set. A single band within that restricted set spans the cutoff (Michelsen et al,[8] 2023; 40-52 years); it is assigned to the older group by its band midpoint throughout, in the meta-regression and in Figures 4A and 4B alike. The alternative configuration, in which a band spanning the cutoff is excluded, is reported in **eTable 20**, together with the effect of the corresponding assignment for the open ≥40-year band of Xing et al,[2] 2024 in the extended analysis sets.

The subgroup diamonds shown in Figures 4A and 4B pool age bands across cohorts, and the accompanying test for subgroup differences treats those bands as independent. Because several bands originate from the same cohort and often share a comparator group, that test is anti-conservative, and the stratified figures are therefore interpreted descriptively. The formal contrast across 45 years is instead estimated with cohorts as the unit of inference, as follows. Within each cohort the band-specific estimates were combined by fixed-effect inverse-variance meta-analysis into 1 estimate for bands with midpoints below 45 years and 1 estimate for bands at or above 45 years. The cohort-specific contrast is the ratio of the second to the first, obtained on the log scale as the difference between the 2 combined log HRs, with variance equal to the sum of their variances. Cohorts contributing bands on only 1 side of the cutoff yield no contrast and do not enter this analysis. The cohort-specific contrasts were then pooled by random-effects meta-analysis with REML estimation of the between-cohort variance and the Knapp-Hartung adjustment, which is appropriate to the small number of contributing cohorts. The resulting ratio of hazard ratios is the quantity reported as the formal age contrast in the main article: a value below 1 indicates that the association with mortality is weaker when iatrogenic menopause occurs at or after 45 years. The same contrast computed with age bands treated as independent is reported alongside it for comparison only (**eTable 20**).

Age at iatrogenic menopause was additionally modelled as a continuous moderator by meta-regression of log HRs on the midpoint of each reported band. Closed bands took their arithmetic midpoint and open-ended bands a midpoint 3 years beyond the reported cutoff, with 2 exceptions that are identified in eTable 19; the assigned values were varied by 3 years in either direction as a sensitivity analysis. The midpoint assigned to every band is listed in eTable 19. Bands spanning the 45-year cutoff enter these models at their midpoints, so the continuous specification requires no assignment rule. Clustering was defined by cohort rather than by publication, so that overlapping publications from the same underlying cohort formed a single cluster. Because several cohorts contributed more than 1 age band, inference was based on a 2-stage model: a slope was estimated within each cohort by fixed-effect methods, and the cohort-specific slopes were then pooled by a random-effects model with the Knapp-Hartung adjustment. Cohorts, rather than individual age bands, were therefore the unit of inference throughout, and models treating age bands as independent are reported only for comparison. Between-cohort heterogeneity in the fitted slopes is a different quantity from heterogeneity in the cohort-level contrast across 45 years, and both are reported (**eTable 20**).

The gradient was further decomposed into its within-cohort and between-cohort components by entering each cohort's mean age at menopause alongside the deviation of each band from that mean, which separates variation in age at surgery among women within a cohort from variation in mean age at surgery among cohorts. The

© 2026 Armeni E. *JAMA Network Open*. This is an open access article distributed under the terms of the CC-BY-NC-ND License, which does not permit alteration or commercial use, including those for text and data mining, AI training, and similar technologies.

between-cohort term is estimated from as many observations as there are cohorts and does not support formal inference; it is reported to show which of the 2 sources any gradient arises from (**eTable 20**).

Nonlinearity was examined using a quadratic term, linear splines with knots at 45 and 50 years, and restricted cubic splines with 3 knots, compared with the linear model by likelihood-ratio test and by the Akaike and Bayesian information criteria. No nonlinear specification was supported for either mortality outcome, and a quadratic term was likewise not supported for any of the 3 cardiovascular event outcomes, so the linear model was retained as the sole continuous specification for every outcome (eTable 20). The age bands reported by each cohort, the midpoint assigned to each band and the age interval across which each cohort-level contrast is measured are recorded in eTable 19; that interval varies substantially across cohorts and was itself examined as a moderator of the contrast. Analyses restricted to ages younger than 45 years, and analyses restricted to cohorts comparing each age band with a referent group matched to that band, were post hoc and are considered exploratory. The role of the age at iatrogenic menopause as a continuous moderator, pooled across cardiovascular morbidity and mortality endpoints, with independent source populations as the unit of inference is presented in **eTable 21**.

Menopausal hormone therapy as an exploratory moderator

Menopausal hormone therapy was examined as a prespecified but exploratory moderator. Because hormone therapy prevalence was reported by exposure group in only 6 of the 9 cohorts contributing to all-cause mortality, and by age band in only 1 of those 6, hormone therapy could be entered only as a cohort-level covariate; it cannot be separated from age at surgery, which is itself a strong determinant of prescribing. Two specifications were fitted, both by random-effects meta-regression of the log HR with the between-study variance estimated by REML: the difference in hormone therapy prevalence between the exposed and comparator arms, and the prevalence in the exposed arm alone. A third specification, restricting the difference-in-prevalence model to cohorts with a no-oophorectomy or no-pelvic-surgery referent, is reported as a sensitivity analysis.

Inference for these models rests on the Knapp-Hartung adjustment, which is appropriate to the small number of contributing cohorts. Wald intervals are reported alongside the Knapp-Hartung intervals in **eTable 23**, because the Knapp-Hartung intervals are those corresponding to the reported *P* values; both are presented so that the comparison between the 2 methods is visible rather than implicit. Robustness was assessed by leave-one-out omission of each cohort in turn and by an exact permutation test over all permutations of the moderator (**eTable 23**). For cardiovascular mortality the same information was available for only 3 of the 7 contributing estimates, leaving 1 residual degree of freedom, so no model was fitted. Within-cohort modification by hormone therapy stratum is reported separately for those cohorts that report both strata (**eTable 22**).

Sensitivity analyses

Four sensitivity analyses were applied to the pooled outcomes, each conducted for every pooled estimate irrespective of the observed heterogeneity. First, each pooled estimate was refitted under a fixed-effect inverse-variance model and compared with the random-effects estimate, which remained primary throughout (**eTable 9**). Second, leave-one-out analyses omitted each contributing cohort in turn and re-pooled the remainder (**eTable 16**); for the age-stratified mortality analyses this was extended to omission of each individual age band (**eTable 18**). Third, the comparator stratification and the graded referent-purity restriction described above were applied to the pooled estimates (**eFigures 1-5** and **eTable 20**). Fourth, because 2 publications report cardiovascular mortality from the same Olmsted County population, that analysis was repeated retaining each report alone and excluding both (**eTable 17**). Two further sensitivity analyses are internal to the meta-regression and are described above: the alternative assignment of age bands spanning the 45-year cutoff, and variation of the midpoints assigned to open-ended bands.

Deviations from the registered protocol

The protocol registered in PROSPERO (CRD420251033146) did not designate a primary outcome. All-cause mortality is presented first because it is the outcome with the largest number of contributing cohorts and the only one for which age at iatrogenic menopause could be modelled across the full range of reported ages. This ordering is a matter of presentation and not a prespecified hypothesis.

The dichotomous contrast at 45 years was prespecified on clinical grounds and was not derived from the data. The following analyses were not prespecified, were undertaken after the data had been extracted, and are reported throughout as exploratory: the stratification of every pooled outcome by comparator type (**eFigures 1-5**); the graded referent-purity restriction applied to the age-stratified mortality analyses (**eTable 20**); the modelling of age at iatrogenic menopause as a continuous moderator pooled across cardiovascular morbidity and mortality endpoints, with independent source populations as the unit of inference (**eTable 21**); the analyses restricted to ages below 45 years; and the analyses restricted to cohorts comparing each age band with a referent group matched to that band.

© 2026 Armeni E. *JAMA Network Open*. This is an open access article distributed under the terms of the CC-BY-NC-ND License, which does not permit alteration or commercial use, including those for text and data mining, AI training, and similar technologies.

Menopausal hormone therapy was prespecified as a moderator but is reported as exploratory rather than as a planned subgroup analysis, because prevalence by exposure group was reported by only 6 of the 9 cohorts contributing to all-cause mortality and by only 3 of the 7 estimates contributing to cardiovascular mortality, so the models are fitted on a non-random subset of the evidence and hormone therapy cannot be separated from age at surgery (**eTables 22 and 23**).

Two elements of the analysis plan could not be executed as registered. Publication bias and small-study effects were to be assessed but were not, because fewer than 10 studies contributed to each synthesis, which precludes reliable evaluation by funnel plot or statistical test. Formal comparison of subgroups defined by baseline cardiovascular risk was not undertaken; those outcomes are summarised qualitatively instead (**eTables 14 and 15**).

No adjustment was made for multiplicity across outcomes or across the exploratory analyses listed above, and the results should be read accordingly.

Software, certainty of evidence and publication bias

Pooled and subgroup analyses were performed in Cochrane Review Manager Web (The Cochrane Collaboration). Meta-regression, cohort-clustered analyses, 2-stage cohort-slope models, the permutation test and the tests of nonlinearity were performed in R version 4.6.1 (R Foundation for Statistical Computing, Vienna, Austria) using the metafor package (version 4.8-0).

Publication bias and small-study effects were not formally assessed, because the small number of studies contributing to each synthesis precluded reliable evaluation by funnel plot or statistical test. Certainty of evidence was assessed for each pooled outcome using the GRADE approach, considering risk of bias, inconsistency, indirectness, imprecision and publication bias, and allowing rating up where all plausible residual confounding would be expected to reduce rather than to create the observed association (**eTable 10**). Certainty was rated against the random-effects estimate that is primary for each outcome. Inconsistency was judged on the between-study variance and the dispersion of the contributing estimates, and imprecision on whether the pooled interval excluded the null and on the stability of that conclusion in leave-one-out analysis (**eTable 16**). No rating up was applied where no effect was demonstrated.

© 2026 Armeni E. *JAMA Network Open*. This is an open access article distributed under the terms of the CC-BY-NC-ND License, which does not permit alteration or commercial use, including those for text and data mining, AI training, and similar technologies.

eResults

Patient characteristics

Study characteristics. The design, setting, source population, sample size, cause of iatrogenic menopause and comparator definition of each included study are summarised in eTable 11, and the corresponding anthropometric and menopause-related characteristics in eTable 12. In the sample size column of eTable 11, N is the total analytic sample as reported by the source study, whereas the case and comparator figures are the specific arms that enter this review. The two do not always sum. Published totals are generally counted before losses to follow-up and before exclusions for missing data on exposure, outcome or covariates, and several studies report additional groups that were not extracted here; the counts contributing to each pooled estimate are those given in eTable 9.

Loss to follow-up. Of the 36 included studies, 9 (25.0%) were cross-sectional or case–control in design and therefore not susceptible to attrition, and this was recorded as not applicable; 2 further cross-sectional studies (5.6%; Indhavivadhana et al,[28] 2011; Jamali et al,[29] 2024) were recorded as not reported in the risk-of-bias tables, although the same design consideration applies to them. Fifteen studies (41.7%) did not report participant retention or attrition. The remaining 10 studies (27.8%) described or quantified loss to follow-up, and in all instances attrition was low. Several cohorts reported minimal or negligible loss without quantification (Appiah et al,[5] 2015; Michelsen et al,[8] 2023; Rivera et al,[7] 2009), while others provided explicit figures: fewer than 5 participants lost in total (Olesen et al,[14] 2022), 4 women lost from the comparator group only (Abildgaard et al,[16] 2018), 32 of 3,474 participants (Rocca et al,[6] 2006), annual attrition below 2.8% (Thong et al,[24] 2022), and 3,184 of 282,722 women (1.1%) lost to follow-up alongside 18,434 deaths (6.4%) over the observation period in a large population-based study (Poorthuis et al,[9] 2022). The highest attrition was observed in the multi-phase prospective study by Gierach et al,[10] 2014, with losses of 15.6%, 10.6% and 3.5% between successive study phases. Registry- and population-based cohorts generally achieved virtually complete follow-up through linkage to national health and mortality registers, including Wilson et al,[13] 2019, in which death data were complete for all participants through linkage to the National Death Index. Overall, reported attrition was well below the conventional 20% threshold of concern in every study that quantified it, and incomplete reporting of follow-up was taken into account in the risk-of-bias assessment.

Missing data. Of the 36 included studies, 17 (47.2%) did not report whether missing values were present or how they were handled, and 1 cross-sectional study (2.8%; Dorum et al,[27] 2008) recorded this as not applicable. The remaining 18 studies (50.0%) described their approach. Eleven excluded participants with incomplete exposure, outcome or covariate information, including Olesen et al,[14] 2022, in which 3,393 participants with missing covariate data were excluded and those excluded had more missing lifestyle information, and Ou et al,[32] 2023, in which 132 participants were excluded. Six used multiple imputation (Dam et al,[18] 2019; Honigberg et al,[12] 2019; Wilson et al,[13] 2019; Xing et al,[2] 2024; Xu et al,[3] 2022; Zhu et al,[15] 2020), and in 4 of these imputation was applied in sensitivity analyses alongside complete-case primary analyses; where reported, results after imputation were consistent with the primary findings. Four of the 11 studies that excluded participants also used imputation. One study stated explicitly that missing data were not imputed (Appiah et al,[4] 2020), and 4 quantified the extent of missing data without describing a specific method (Do Valle et al,[19] 2021; Indhavivadhana et al,[28] 2011; Jamali et al,[29] 2024; Tuesley et al,[25] 2020). Because no study provided participant-level data, the extent of missing data could not be quantified uniformly across studies; incomplete reporting was taken into account in the risk-of-bias assessment.

Age at iatrogenic menopause

Contrast across 45 years. In the primary analysis set, which comprises all contributing cohorts, the pooled estimate for age bands at or above 45 years divided by the pooled estimate for bands below 45 years was 0.82 (95% CI, 0.68-0.98; *P* = .04) for all-cause mortality, from 25 age bands in 7 cohorts, and 0.77 (95% CI, 0.58-1.04; *P* = .07) for cardiovascular mortality, from 18 bands in 5 cohorts (eTable 20). Bands spanning the cutoff are assigned to the older group by band midpoint, as in Figures 4A, 4B and 5. The corresponding estimates under each comparator restriction and under the alternative rule of excluding the band that spans the cutoff are tabulated in eTable 20 and are not repeated here. Treating age bands as independent observations, as the subgroup-difference tests shown on Figures 4A and 4B do, returned similar point estimates with substantially smaller *P* values (0.84, *P* = .003, and 0.74, *P* = .004, in the primary set). Because several cohorts contribute more than 1 band, those tests are anti-conservative and are reported for comparison only.

© 2026 Armeni E. *JAMA Network Open*. This is an open access article distributed under the terms of the CC-BY-NC-ND License, which does not permit alteration or commercial use, including those for text and data mining, AI training, and similar technologies.

Referent purity. Restricting the referent progressively reduces the number of contributing cohorts and does not move the contrast in a consistent direction. The 2 intermediate restrictions move the point estimate further from the null than the primary analysis, which is the direction predicted if a referent group containing women with hysterectomy alone biases the contrast towards the null, but the strictest restriction returns to 0.82 and no restricted analysis reached conventional significance, because each restriction removes a cohort. The tier definitions and the estimate at each tier are given in eTable 20.

Cohorts using a natural-menopause referent. Two cohorts compare women with iatrogenic menopause with women who underwent natural menopause rather than with women who had not undergone surgery, and both contribute to the primary analysis. The all-cause contrast reaches conventional significance only when both are included, and that result depends on the treatment of the open 40-year and older band reported by Xing et al,[2] 2024, which spans the cutoff: assigned to the older group by its midpoint the cohort contributes a contrast, whereas excluded it contributes only a band below 45 years and therefore no contrast at all, and the analysis reverts to the 6-cohort estimate of 0.85 (95% CI, 0.69-1.04; *P* = .09). Both cohorts are excluded from the comparator-restricted sensitivity ladder on referent grounds, but neither is excluded from the primary analysis (eTable 20).

Stability of the contrast. Across the 10 estimable mortality specifications reported in eTable 20 the cohort-clustered ratio of hazard ratios ranged from 0.74 to 0.85, while the corresponding *P* values ranged from .04 to .29. The magnitude and direction of the contrast are therefore stable across analysis sets, comparator restrictions and band-assignment rules; it is the precision of the contrast, and not its size, that these data cannot support.

Age as a continuous moderator. Modelled continuously across the complete set of disjoint age bands, the hazard ratio changed by a factor of 0.94 per 5-year increment in age at iatrogenic menopause for all-cause mortality (95% CI, 0.86-1.02; *P* = .12; 25 estimates from 7 cohorts) and 0.92 per 5 years for cardiovascular mortality (95% CI, 0.79-1.08; *P* = .22; 18 estimates from 5 cohorts) with cohorts as the unit of inference (Figure 5 and eTable 20). Cohorts did not agree on the gradient: between-cohort heterogeneity in the fitted cohort-specific slopes was significant for both outcomes (Q = 18.3; df = 6; *P* = .006 for all-cause mortality and Q = 11.0; df = 4; *P* = .03 for cardiovascular mortality). This is heterogeneity in the slope and is a different quantity from the heterogeneity in the cohort-level contrast across 45 years, which is reported in eTable 20. Treating age bands as independent gave 0.95 per 5 years (95% CI, 0.92-0.98; *P* = .002) and 0.91 per 5 years (95% CI, 0.85-0.97; *P* = .003). The 2 routes differ in inference rather than in estimation, the point estimates being almost identical and the intervals differing by a factor of approximately 3 in width.

Within-cohort and between-cohort components. Entering each cohort's mean age at menopause alongside the deviation of each band from that mean separates the 2 sources of the gradient (eTable 20). For both mortality outcomes the within-cohort component carried the gradient while the between-cohort component was null. Whatever gradient is present is therefore expressed within cohorts and is not generated by differences between cohorts in mean age at surgery. Both components are estimated treating age bands as independent, and the between-cohort term is estimated from as many observations as there are cohorts, so neither supports formal inference.

Age interval across which each contrast is measured. The interval between the mean age of the group below 45 years and the mean age of the group at or above 45 years varies just over threefold across cohorts, from 5.5 to 16.7 years, so a cohort with a narrow interval measures a smaller contrast for the same underlying gradient (eTable 19). That does not explain the disagreement between cohorts: the unweighted correlation between the age interval and the log ratio of hazard ratios is −0.09 for all-cause mortality and −0.10 for cardiovascular mortality, weighted regression of the contrast on the interval gives *P* = .52 and *P* = .61 respectively, and substantial heterogeneity remains after adjustment for all-cause mortality (residual Q = 23.67; df = 5; *P* < .001) though not for cardiovascular mortality (Q = 5.72; df = 3; *P* = .13). The per-year equivalents in eTable 19 are contrast-based and are not identical to the per-cohort slopes fitted by the continuous model in Figure 5, agreeing in sign for all but 1 cohort on each outcome, Michelsen et al,[8] 2023 in both cases, but differing in magnitude by up to twofold.

Cardiovascular event outcomes. No contrast across 45 years could be constructed for the event outcomes, because the contributing cohorts reported age bands divided at 48, 49 or 50 years and no band boundary at 45 years was available in more than 1 cohort; age was therefore modelled continuously for these outcomes, and the fitted gradients are given in eTable 20. Composite cardiovascular events is the only outcome in the review for which the age gradient approaches conventional significance once cohorts are the unit of inference. Between-cohort disagreement was more marked than for the mortality outcomes, Poorthuis et al,[9] 2022 showing no gradient for composite cardiovascular events and a gradient in the opposite direction for coronary heart disease.

© 2026 Armeni E. *JAMA Network Open*. This is an open access article distributed under the terms of the CC-BY-NC-ND License, which does not permit alteration or commercial use, including those for text and data mining, AI training, and similar technologies.

Nonlinearity. No nonlinear specification was supported for any outcome, and the linear model was preferred on both the Akaike and Bayesian information criteria; the comparisons are reported in eTable 20. Only 1 cohort reported more than 2 age bands for the event outcomes, so curvature there could be estimated only across cohorts and not within them; the linear model was retained throughout.

Analyses restricted to ages below 45 years. Within the range below 45 years the all-cause hazard ratio changed by a factor of 0.94 per 5-year increment (95% CI, 0.88-1.00; *P* = .05), estimated from the 3 of 7 cohorts reporting more than 1 age band below 45 years, and the contributing cohort slopes were concordant in direction ($Q = 0.48$; df = 2; *P* = .79). Restricting to cohorts whose referent comprised women without oophorectomy or pelvic surgery leaves 2 informative cohorts, and the gradient is no longer supported (0.94 per 5 years; 95% CI, 0.73-1.20; *P* = .18). For cardiovascular mortality, 2 cohorts report more than 1 age band below 45 years (Tuesley et al,[25] 2020: <35 and 35-44 y; Xu et al,[3] 2022: <40 and 40-44 y; eTable 19), so a within-range gradient would rest on 1 residual degree of freedom and was not estimated; unlike the all-cause restriction above, which is a sensitivity analysis of a better-supported estimate, it would constitute the primary estimate itself.. This analysis was post hoc, rests on 2 residual degrees of freedom in its primary form, and is reported as exploratory.

Age matching and exposure definition. Only 2 cohorts compare each age band with a referent group matched to that band rather than with a single common referent group (Gierach et al,[10] 2014 and the Olmsted County cohort [Rivera et al,[7] 2009; Rocca et al,[6] 2006]); in every other cohort each band is compared with 1 shared referent group, so the band-specific estimates within those cohorts are positively correlated and their standard errors are conservative. Restricted to the 2 band-matched cohorts, only 1 reports more than 1 band below 45 years and no pooled within-range gradient exists. For the Olmsted County cohort (Rivera et al,[7] 2009; Rocca et al,[6] 2006) the all-cause age strata describe women undergoing oophorectomy for prophylactic indications whereas the pooled estimate entered in Figure 3B describes all indications; the consequences of that mismatch are quantified in eTable 18 and do not alter the pooled all-cause estimate.

Sensitivity analyses

Fixed-effect models. Under a fixed-effect model the estimates for composite cardiovascular events, coronary heart disease and all-cause mortality lay closer to the null than the corresponding random-effects estimates, with narrower intervals, because the fixed-effect model gives greater weight to the largest cohorts; all-cause mortality nonetheless remained significant and cardiovascular mortality remained non-significant. Stroke was identical under both models, because restricted maximum likelihood returns a between-study variance of 0 for that outcome so that the random-effects weights reduce to the fixed-effect weights. The fixed-effect summaries, with the corresponding Cochran Q and I2 statistics, are presented in eTable 9, where the random-effects estimates are restated for comparison. Random-effects estimates are primary throughout irrespective of observed heterogeneity, as stated in the Methods, and the fixed-effect analysis is presented for comparison only.

Leave-one-out analyses. Every pooled outcome was refitted with each contributing cohort omitted in turn; the recalculated estimates, intervals and heterogeneity statistics are given in eTable 16 and only the conclusions are drawn here. The composite cardiovascular events estimate excluded the null in every iteration, and its heterogeneity is attributable to a single cohort: omission of Farland et al,[11] 2023 reduces I^2 from 86% to 0%. Coronary heart disease is the one morbidity outcome that loses conventional significance, on omission of the same cohort; omission of Honigberg et al,[12] 2019 leaves substantial residual heterogeneity, so no comparable attribution of heterogeneity to a single cohort holds for that outcome. Stroke was significant in every iteration. Cardiovascular mortality remained non-significant in every iteration, so the null result does not depend on any single report; because its 7 estimates arise from 6 cohorts, however, no iteration removes the duplicated Olmsted County population in full (eTable 17). All-cause mortality is the least robust estimate: it loses conventional significance on omission of Xu et al,[3] 2022 and sits at the threshold on omission of Michelsen et al,[8] 2023, whereas omission of Xing et al,[2] 2024, the other cohort using a natural-menopause referent, leaves it significant. Significance is therefore not simply a function of comparator definition, since 1 of the 2 influential cohorts uses a no-oophorectomy referent.

Leave-one-out analyses of the age-stratified estimates. For the age-stratified mortality analyses the procedure omits each individual age-band estimate rather than each cohort: 13 estimates from 4 cohorts for cardiovascular mortality, 5 below and 8 at or above 45 years, and 18 estimates from 5 cohorts for all-cause mortality, 8 and 10 respectively (eTable 18). Both subgroup estimates are robust: the estimate below 45 years excluded the null in every iteration and the estimate at or above 45 years included it in every iteration, for both outcomes. What moves is the test between strata, which treats age bands as independent and is anti-conservative; it crosses the

© 2026 Armeni E. *JAMA Network Open*. This is an open access article distributed under the terms of the CC-BY-NC-ND License, which does not permit alteration or commercial use, including those for text and data mining, AI training, and similar technologies.

conventional threshold in 2 of the 13 cardiovascular mortality iterations while remaining below it in all 18 all-cause iterations. The largest strengthening of the contrast follows omission of the estimate for women aged 53 years or older reported by Michelsen et al,[8] 2023, an older band with an elevated hazard ratio that therefore runs counter to a monotonic age gradient; it is also the only omission that materially reduces heterogeneity in the subgroup at or above 45 years for both outcomes. Omitting the band that spans the cutoff, reported by the same cohort for both outcomes, reproduces the alternative configuration in which spanning bands are excluded. Two further rows address the difference in exposure definition for the Olmsted County cohort, whose all-cause age strata describe prophylactic oophorectomy alone: neither omitting its stratum below 45 years nor omitting the cohort entirely materially alters the subgroup estimates. Inference on the age contrast rests instead on the cohort-clustered analysis reported in eTable 20.

Comparator type. Each pooled outcome was reanalyzed with the contributing cohorts grouped by the referent population used for comparison, and the 5 panels are presented as eFigures 1 to 5. These analyses were post hoc and are reported as exploratory. The test for subgroup differences reached conventional significance for coronary heart disease only (χ^2 = 23.28; df = 2; P < .001; eFigure 2), in which the single cohort restricted to menopause before age 40 years forms its own referent group, so that comparator type is confounded with age at menopause; the difference is absent when that cohort is omitted (χ^2 = 0.07; df = 1; P = .79). The test did not reach conventional significance for any other outcome; the stratum-specific estimates and heterogeneity statistics are shown on the eFigures. For all-cause mortality the referent was natural menopause in 2 cohorts (1.16; 95% CI, 1.06-1.27) and the absence of surgery in the remainder (1.06; 95% CI, 0.99-1.14). In 4 of the 5 outcomes at least 1 referent group is represented by a single cohort, so these comparisons have little power, and no adjustment was made for testing across 5 outcomes. The graded referent-purity restriction applied to the age-stratified mortality analyses is reported above (eTable 20).

Overlapping reports of the same cohort. Two reports of the Olmsted County cohort (Rocca et al,[6] 2006 and Rivera et al,[7] 2009) contribute to the pooled analysis of cardiovascular mortality, so that population is counted twice and carries 16.9% of the pooled weight. Excluding one or both reports did not materially alter the estimate, which remained non-significant in every configuration, and removing the duplicated population moved the estimate away from the null and reduced heterogeneity (eTable 17). Which of the 2 reports is retained is immaterial; only the decision to count the cohort once or twice affects the result.

Certainty of evidence. Certainty was rated for each pooled outcome by the GRADE approach, applied to the random-effects estimate that is primary for that outcome; the domain-by-domain ratings, and the reasons for rating up or down in each domain, are given in eTable 10. All contributing studies are non-randomized, so every outcome started at low certainty. Certainty is moderate for stroke, the only outcome in which no heterogeneity is detected under the random-effects model and for which rating up was applied because residual confounding in the contributing cohorts would be expected to reduce rather than to create the association; low for composite cardiovascular events; and very low for coronary heart disease and for both mortality outcomes. No rating up was applied to either mortality outcome, because no effect was demonstrated. Publication bias was not formally assessed, because the number of studies contributing to each synthesis precluded reliable evaluation.

Menopausal hormone therapy (exploratory)

Cohort-level meta-regression. In all 6 cohorts reporting prevalence by exposure group, prevalence was higher in the exposed arm, by between 16.8 and 63.2 percentage points; the largest difference is that of Wilson et al,[13] 2019, which also carries the highest all-cause hazard ratio in the set (eTable 12). Cohorts reporting prevalence had higher pooled hazard ratios than the full sets for both outcomes, so these models are fitted on a non-random subset of the evidence; the comparison is quantified in eTable 23.

Neither specification supported modification, and both Knapp-Hartung intervals include 1.00 (eTable 23). Confounding by age at surgery cannot in any case be quantified, because eTable 12 reports attained age rather than age at surgery, in 5 different formats across these cohorts.

Hormone therapy can only enter these models as a between-cohort covariate, since only 1 cohort reported prevalence by age band. In the decomposition of the age moderator into its within-cohort and between-cohort components, the between-cohort component carried no signal (0.99 per 5 years; 95% CI, 0.89-1.10; P = .79; eTable 20), so the covariate is confined to precisely the contrast in which this dataset has no resolving power.

© 2026 Armeni E. *JAMA Network Open*. This is an open access article distributed under the terms of the CC-BY-NC-ND License, which does not permit alteration or commercial use, including those for text and data mining, AI training, and similar technologies.

Within-cohort modification. The confidence intervals of the 3 tabulated contrasts are wide and none excludes the null (eTable 22). Estimates by hormone therapy stratum are also reported in eTable 13 for Olesen et al,[14] 2022 (a 2-stratum contrast for cardiovascular morbidity rather than mortality) and Zhu et al,[15] 2020 (by age band, against a single common referent), and for further cohorts reporting 1 stratum only; none contributes to an all-cause mortality contrast and they are not tabulated in eTable 22. Abildgaard et al,[16] 2018 reported both strata but is also excluded, for the reason given in the footnote to eTable 22.

These analyses do not permit any conclusion about whether hormone therapy modifies the association between age at iatrogenic menopause and mortality, and this is reported as a limitation of the available evidence rather than as a finding.

© 2026 Armeni E. *JAMA Network Open*. This is an open access article distributed under the terms of the CC-BY-NC-ND License, which does not permit alteration or commercial use, including those for text and data mining, AI training, and similar technologies.

eTable 1. Newcastle–Ottawa Scale quality assessment for cohort and case-control studies

Study (Year)	Population / Focus	Selection (4★)	Comparability (2★)	Outcome / Exposure (3★)	Total (9★)
Abildgaard et al,[16] 2018	Cohort study	4	2	3	9
Appiah et al,[5] 2015	Cohort, multicenter	4	2	3	9
Appiah et al,[4] 2020	Cohort	4	2	3	9
Dam et al,[18] 2019	Case-cohort study	4	2	2	8
Do Valle et al,[19] 2021	Population-based matched cohort; RRBSO in BRCA mutation carriers vs controls	4	2	3	9
Duan et al,[20] 2012	Cohort study	4	2	1	7
Farahmand et al,[21] 2014	Cohort study	4	1	3	8
Farland et al,[11] 2023	NHS II cohort; hysterectomy ± oophorectomy & CVD risk	4	2	2	8
Gierach et al,[10] 2014	BCDDP cohort; benign gynecologic surgery & mortality	4	2	3	9
Honigberg et al,[12] 2019	UK Biobank; surgical vs natural menopause & CVD	4	2	2	8
Howard et al,[22] 2005	WHI Observational Study; hysterectomy ± oophorectomy and CVD risk	4	2	3	9
Ingelsson et al,[1] 2010	Cohort, Sweden, Inpatient Register data	4	1	3	8
Krul et al,[23] 2018	Female Hodgkin lymphoma survivors, POI vs on-POI	4	2	3	9
Michelsen et al,[8] 2023	HUNT-2 cohort; hysterectomy ± oophorectomy & mortality	4	2	3	9
Olesen et al,[14] 2022	Danish registry; hysterectomy ± BSO & CVD	4	2	3	9
Poorthuis et al,[9] 2022	Cohort study (China Kadoorie Biobank)	4	2	2	8
Rivera et al,[7] 2009	Olmsted County cohort; unilateral vs bilateral oophorectomy & CVD mortality	4	2	3	9
Rocca et al,[6] 2006	Olmsted County cohort; bilateral oophorectomy & mortality	4	2	3	9
Thong et al,[24] 2022	Western Australia data linkage; iatrogenic early menopause & mortality	4	1	3	8
Tuesley et al,[25] 2020	Australian registry; hysterectomy ± BSO & chronic disease	4	2	2	8

© 2026 Armeni E. *JAMA Network Open*. This is an open access article distributed under the terms of the CC-BY-NC-ND License, which does not permit alteration or commercial use, including those for text and data mining, AI training, and similar technologies.

**Study (Year)**	**Population / Focus**	**Selection (4★)**	**Comparability (2★)**	**Outcome / Exposure (3★)**	**Total (9★)**
Wilson et al,[13] 2019	ALSWH cohort; hysterectomy ± BSO & mortality	4	2	3	9
Xing et al,[2] 2024	NHANES cohort; age at menopause & mortality	4	2	2	8
Xu et al,[3] 2022	UK Biobank; menopause type, MHT & cause-specific mortality	4	2	2	8

Zhu et al,[15] 2020 was a reanalysis of multiple cohort datasets; therefore, it was not included in the risk-of-bias tables.

© 2026 Armeni E. *JAMA Network Open*. This is an open access article distributed under the terms of the CC-BY-NC-ND License, which does not permit alteration or commercial use, including those for text and data mining, AI training, and similar technologies.

eTable 2. Newcastle–Ottawa Scale quality assessment for cross-sectional studies

Study (Year)	Population / Focus	Selection (5★)	Comparability (2★)	Outcome / Exposure (3★)	Total (10★)
Abbas et al,[26] 2018	PWV in natural and surgical menopause	2	1	2	5
Dorum et al,[27] 2008	HUNT-2 study	3	1	3	7
Indhavivadhana et al,[28] 2011	Thai clinic; metabolic syndrome predictors	3	2	3	8
Jamali et al,[29] 2024	Rafsanjan cohort; menopause type & CVD risk	3	2	2	7
Mack et al,[30] 2004	EPAT subset; carotid IMT & menopause type	1	2	2	5
Netterlid et al,[31] 2021	Female childhood cancer survivors, POI vs non-POI	3	1	2	6
Ou et al,[32] 2023	Taiwan Biobank; menopause type & metabolic syndrome	4	2	3	9
Ozdemir et al,[33] 2009	Turkish clinic; metabolic syndrome & BMD	3	1	2	6
Ozkaya et al,[34] 2011	Turkish clinic; IMT, BMD, homocysteine	3	2	2	7
Tuysuzoglu et al,[35] 2020	Turkish cohort; metabolic syndrome, BMD, vasomotor symptoms	2	1	2	5

Abbreviations: ALSWH, Australian Longitudinal Study on Women's Health; BCDDP, Breast Cancer Detection Demonstration Project; BRCA, breast cancer susceptibility gene; BSO, bilateral salpingo-oophorectomy; CAC, coronary artery calcium; CVD, cardiovascular disease; HUNT-2, Nord-Trøndelag Health Study (second survey); MHT, menopausal hormone therapy; NHANES, National Health and Nutrition Examination Survey; POI, premature ovarian insufficiency; RCT, randomized clinical trial; RRBSO, risk-reducing bilateral salpingo-oophorectomy; UK, United Kingdom; WHI, Women's Health Initiative.

© 2026 Armeni E. *JAMA Network Open*. This is an open access article distributed under the terms of the CC-BY-NC-ND License, which does not permit alteration or commercial use, including those for text and data mining, AI training, and similar technologies.

eTable 3. Cochrane Risk of Bias 2.0 assessment for randomized clinical trials

Study (year)	Domain 1: Randomization process	Domain 2: Deviations from intended interventions	Domain 3: Missing outcome data	Domain 4: Measurement of outcome	Domain 5: Selection of reported result	Overall RoB
Allison et al,[17] 2008 (WHI–Coronary Artery Calcium Substudy)	Low risk -Random sequence and allocation concealment performed within parent WHI-CEE RCT; baseline comparability confirmed.	Low risk -Double-blind placebo-controlled design; interventions delivered per protocol; ITT analysis used.	Some concerns -Only 62% of eligible women (1,079/1,742) underwent CAC scanning; participation bias possible if related to outcome.	Low risk -CAC measured using standardized CT protocol; central blinded reading; objective outcome.	Low risk -Analytic plan pre-specified; logistic regression adjusted for WHI assignment and confounders; all outcomes reported.	Some concerns -Primarily due to post-trial selection/participation bias in the substudy.
Chen et al,[36] 2023 (Early vs Late Intervention Trial with Estradiol -ELITE)	Low risk -Computer-generated 1:1 randomization stratified by menopausal stage; allocation concealed; baseline balance achieved.	Low risk -Double-blind, placebo-controlled; identical appearance of active/placebo; adherence and blinding maintained; ITT analysis used.	Low risk -High retention; repeated-measures mixed-effects model addressed missing data; no differential dropout.	Low risk -CIMT assessed by blinded central ultrasonographers using standardized protocol and centralized image analysis.	Low risk -Pre-specified analysis registered on ClinicalTrials.gov (NCT00114517); transparent reporting of primary and secondary endpoints.	Low risk -No major sources of bias identified; robust randomization and blinding maintained throughout.

Abbreviations: CAC, coronary artery calcium; CEE, conjugated equine estrogen; CIMT, carotid intima-media thickness; CT, computed tomography; ITT, intention-to-treat; RCT, randomized clinical trial; RoB, risk of bias; WHI, Women's Health Initiative.

© 2026 Armeni E. *JAMA Network Open*. This is an open access article distributed under the terms of the CC-BY-NC-ND License, which does not permit alteration or commercial use, including those for text and data mining, AI training, and similar technologies.

eTable 4. Studies reporting on absolute, relative, and incident risk for cardiovascular disease and coronary heart disease.

Study (Year)	CVD -Absolute/Relative risk	CVD -Incident risk	CHD -Absolute / Relative risk	CHD -Incident risk
Abbas et al,[26] 2018	Hysterectomy+BSO vs Naturally menopaused women, Absolute risk (mean ± SD)<br>cSBP (mmHg): 117.7 ± 16.2 vs. 123.44 ± 20.7<br>cDBP (mmHg): 80.2 ± 7.9 vs. 80.4 ± 10.3<br>cAP (mmHg): 7.3 ± 5.8 vs. 10.9 ±7.9<br>cfPWV (cm/s): 1104.4 ± 234.6 vs. 1061.03 ± 362.23<br>baPWV (cm/s): 1775.8 ± 604.12 vs. 1573.2 ± 544.6<br>ABI: 1.13 ± 0.23 vs. 1.14 ± 0.093<br>cPP (mmHg): 44 ± 13.9 vs 37.5 ± 14.1	NR	NR	NR
Allison et al,[17] 2008	BSO vs non-BSO (OR (95% CI) :<br>CAC score >1: 1.27 (0.94-1.71)	NR	NR	NR
Chen et al,[36] 2023	NR	Hysterectomy+BSO vs. natural menopause<br>Baseline CIMT (microm):<br>Overall β (95% CI):<br>3.1 (-21.72-27.91)<br>Age at menopause β (95% CI):<br>≤ 50 y: -3.83 (-31.82-24.16)<br>>50 y: 24.02 (-22.24-70.29)<br><br>Annual rate of CIMT progression (microm/y):	NR	NR

© 2026 Armeni E. *JAMA Network Open*. This is an open access article distributed under the terms of the CC-BY-NC-ND License, which does not permit alteration or commercial use, including those for text and data mining, AI training, and similar technologies.

Study (Year)	CVD -Absolute/Relative risk	CVD -Incident risk	CHD -Absolute / Relative risk	CHD -Incident risk
		Overall β (95% CI):<br>7.19 (6.22-8.17)<br><br>Difference in CIMT rate (microm/y):<br>Overall β (95% CI):<br>2.49 (-0.07to -0.05)<br>Age at menopause, β (95% CI)<br>≤ 50 y: 1.58 (-1.31-4.48)<br>> 50 y: 5.29 (0.42-10.15)		
Dam et al,[18] 2019	NR	NR	Bilateral oophorectomy vs. natural menopause (n/N):<br>Surgical menopause defined as bilateral ovariectomy only:<br>332/856 vs. 3,747/9,152	Bilateral oophorectomy vs. natural menopause, age-adjusted model (HR, 95% CI):<br>Surgical menopause defined as bilateral ovariectomy only:<br>1.22 (1.01-1.47)
Dorum et al,[27] 2008	Cases vs. comparators, absolute risk (n/N):<br>101/263 vs. 431/789	Cases vs. comparators:<br>Framingham risk score >10%:<br>Absolute risk (n/N): 58/263 vs. 114/789<br>Relative risk OR (95% CI):<br>1.68 (1.18-2.38)	Cases vs. comparators (n/N):<br>CHD events: 11/263 vs. 26/789<br>MI events: 2/263 vs. 6/789	NR
Do Valle et al,[19] 2021	Absolute risk (n/N, %):<br>BRCA+BSO: 25/360 (6.9%)<br>BSO (benign, no mutation): 322/3,600 (8.9%)<br>Intact ovaries, no mutation: 206/3,600 (5.7%)	Incident risk (HR, 95% CI):<br>BSO, BRCA+ vs. BSO, no mutation: 1.30 (0.73-2.32)<br>BRCA+BSO vs. intact ovaries: 1.79 (1.00-3.23)	Absolute risk (n/N, %)<br>MI events:<br>BRCA+BSO: 5/360 (1.4%)<br>BSO (benign, no mutation): 71/3,600 (2.0%)<br>Intact ovaries, no mutation: 46/3,600 (1.3%)	NR
Farland et al,[11] 2023	CVD+coronary revascularization, Absolute risk (n/N):	CVD+coronary revascularization, Incident risk (HR, 95% CI):	MI, Absolute risk (n/N):<br>-BSO+hysterectomy: 204/2,153	MI, Incident risk (HR, 95% CI):

© 2026 Armeni E. *JAMA Network Open*. This is an open access article distributed under the terms of the CC-BY-NC-ND License, which does not permit alteration or commercial use, including those for text and data mining, AI training, and similar technologies.

Study (Year)	CVD -Absolute/Relative risk	CVD -Incident risk	CHD -Absolute / Relative risk	CHD -Incident risk
	Overall:<br>BSO+hysterectomy: 442/2,153<br>Hysterectomy-only: 192/2,960<br>No surgery: 1,357/108,448<br>Age at surgery<br>≤ 50 y:<br>BSO+hysterectomy: 120/NR<br>Hysterectomy-only: 61/NR<br>> 50 y:<br>-BSO+hysterectomy: 322/NR<br>-Hysterectomy only: 131/NR<br><br>CVD (MI and stroke), Absolute risk (n/N):<br>-BSO+hysterectomy: 360/2,153<br>-Hysterectomy-only: 146/2,960<br>-No surgery: 1,067/108,448	Overall:<br>-BSO+hysterectomy: 1.57 (1.40-1.75)<br>-Hysterectomy-only: 1.32 (1.13-1.54)<br>-No surgery (reference category)<br>Age at surgery<br>≤ 50 y<br>-BSO+hysterectomy: 2.12 (1.74-2.58)<br>-Hysterectomy-only: 1.35 (1.03-1.75)<br>> 50 y<br>-BSO+hysterectomy: 1.40 (1.22-1.59)<br>-Hysterectomy only: 1.29 (1.07-1.55)<br><br>CVD (MI and stroke), Incident risk (HR 95% CI):<br>-BSO+hysterectomy: 1.58 (1.39-1.78)<br>-Hysterectomy-only: 1.25 (1.05-1.49)<br>-No surgery, Reference category	-Hysterectomy-only: 80/2,960<br>-No surgery: 529/108,448	BSO+hysterectomy: 1.7 (1.44-2.01)<br>Hysterectomy-only: 1.31 (1.04-1.66)<br>No surgery (reference)
Honigberg et al,[12] 2019	BSO vs. natural menopause, Relative risk (IRR, 95% CI):<br>11.27 (8.54-14.6) vs. 8.78 (7.83-9.82)	BSO <40 y vs. Menopause at age > 40 y, Incident risk (HR, 95% CI):<br>2.21 (1.66-2.92) vs. 1.6 (1.42-1.8)<br><br>Surgical menopause vs. natural menopause, Incident risk (HR, 95% CI):	BSO vs. natural menopause, Relative risk (IRR, 95% CI):<br>4.52 (2.94-6.7) vs. 2.33 (1.87-2.87)	BSO <40 y vs. Menopause at age > 40 y, Incident risk (HR, 95% CI):<br>3.76 (2.42-5.86) vs. 1.81 (1.44-2.28)<br><br>Surgical menopause vs. natural menopause, Incident risk (HR, 95% CI):

© 2026 Armeni E. *JAMA Network Open*. This is an open access article distributed under the terms of the CC-BY-NC-ND License, which does not permit alteration or commercial use, including those for text and data mining, AI training, and similar technologies.

Study (Year)	CVD -Absolute/Relative risk	CVD -Incident risk	CHD -Absolute / Relative risk	CHD -Incident risk
		<30y: 3.4 (1.8-6.6) vs. 1.6 (1.0-2.4)<br><35y: 3.0 (2.1- 4.5) vs. 1.7 (1.4-2.1)<br><40y: 2.4 (1.8-3.2) vs. 1.7 (1.5-2.0)<br><45y: 1.7 (1.4-2.1) vs. 1.5 (1.4-1.6)<br><50y: 1.5 (1.3-1.7) vs. 1.3 (1.2-1.4)<br>≥50 y: Reference		<30y: 6.5 (2.4-17.3) vs. 2.5 (1.2-4.9)<br><35y: 5.4 (3.0-9.8) vs. 2.0 (1.3-3.0)<br><40y: 4.2 (2.7-6.6) vs. 2.0 (1.6-2.5)<br><45y: 1.8 (1.3-2.6) vs. 1.6 (1.4-1.8)<br><50y: 1.6 (1.2-2.0) vs. 1.4 (1.2-1.5)<br>≥50 y: Reference
Howard et al,[22] 2005	Total fatal and non-fatal, Absolute risk (n/N, annual incidence %):<br>Hysterectomy+BSO: 588/18,251, annual 0.7%<br>Hysterectomy-only: 564/18,687, annual 0.65%<br>No surgery: 1,361/52,976, annual 0.54% vs	Total fatal and non fatal, Incident risk (HR, 95% CI):<br>Hysterectomy+BSO vs. no surgery: 1.28 (1.16-1.42)<br>Hysterectomy-only vs. no surgery: 1.23 (1.11-1.36)<br>All hysterectomy vs. no surgery: 1.26 (1.16-1.36)	Absolute risk for hospitalization from MI (n/N, annual incidence %):<br>Hysterectomy+BSO: 206/18,251, annual 0.23%<br>Hysterectomy-only: 204/18,687, annual 0.22%<br>No surgery: 483/52,976, annual 0.18%	NR
Ingelsson et al,[1] 2010	No surgery vs. hysterectomy+BSO vs. BSO-only vs. Hysterectomy-only, Absolute risk (n/N):<br><br>≤ 49y:<br>No surgery: 12,003/359,363<br>Hysterectomy+BSO: 308/8,347<br>BSO-only: 107/2,118<br>Hysterectomy-only: 4,103/104,770	No surgery vs. hysterectomy vs. oophorectomy<br>Incident risk (HR, 95% CI):<br><u>Age stratified:</u><br>≤ 49y: 1 vs. 1.17 (1.13-1.21) vs. 1.31 (1.12-1.53)<br>≥50 y: 1 vs. 0.97 (0.94-1.00) vs. 0.98 (0.82-1.18)	No surgery vs. hysterectomy+BSO vs. BSO-only vs. Hysterectomy-only, Absolute risk (n/N):<br><br>≤ 49y:<br>No surgery: 5,440/359,363<br>Hysterectomy+BSO: 134/8,347<br>BSO-only: 51/2,118<br>Hysterectomy-only: 1,828/104,770	No surgery vs. hysterectomy vs. oophorectomy<br>Incident risk (HR, 95% CI):<br><u>Age stratified:</u><br>≤ 49y: reference vs. 1.15 (1.09-1.21) vs. 1.30 (1.03-1.64)<br>≥50 y: reference vs. 0.94 (0.89-0.98) vs. 0.88 (0.65-1.18)

© 2026 Armeni E. *JAMA Network Open*. This is an open access article distributed under the terms of the CC-BY-NC-ND License, which does not permit alteration or commercial use, including those for text and data mining, AI training, and similar technologies.

Study (Year)	CVD -Absolute/Relative risk	CVD -Incident risk	CHD -Absolute / Relative risk	CHD -Incident risk
	≥50 y:<br>No surgery: 28,314/284,652<br>Hysterectomy+BSO: 1,318/19,789<br>BSO-only: 260/3,000<br>Hysterectomy-only: 4,768/51,535		≥50 y:<br>No surgery: 12,006/284,652<br>Hysterectomy+BSO: 527/19,789<br>BSO-only: 102/3,000<br>Hysterectomy-only: 1,946/51,535	
Jamali et al,[29] 2024	Cases vs. comparators, Relative risk (OR, 95% CI): 1.15 (0.85-1.55)	NR	Cases vs. comparators, Relative risk (OR, 95% CI): 1.17 (0.85-1.59)	NR
Krul et al,[23] 2018	POI vs Menopause ≥ 40 y, CVD: 85/299 vs 108/299<br><br>POI vs premenopausal, CVD: 85/299 vs 107/320	Cumulative incidence at age 70 years, 55.7% (95% CI: 50.4 - 60.7)<br>New onset CVD:<br>POI vs Menopause ≥ 40 y: HR 0.89 (95% CI 0.66–1.22)	POI vs Menopause ≥ 40 y, IHD: 23/299 vs 28/299<br><br>POI vs premenopausal, IHD: 23/299 vs 25/320	New onset IHD:<br>POI vs Menopause ≥ 40 y: HR 1.21 (95% CI: 0.68-2.17)<br>POI vs premenopausal : HR 1.72 (95% CI : 0.82 – 3.60)
Mack et al,[30] 2004	NR	Cases vs. comparators:<br><br>Carotid artery IMT (microm, mean ± SE):<br>Overall:<br>800 ± 20 vs. 750 ± 10<br>Age-stratified<br>≤ 45 y:<br>810 ± 30 vs. 750 ± 50<br>45-50 y:<br>780 ± 30 vs. 770 ± 20<br>> 50 y:<br>770 ± 30 vs. 750 ± 10	NR	NR
Ozkaya et al,[34] 2011	Cases vs. comparators: CIMT (mm): 0.88 ± 0.29 vs. 0.72 ± 0.16	NR	NR	NR

© 2026 Armeni E. *JAMA Network Open*. This is an open access article distributed under the terms of the CC-BY-NC-ND License, which does not permit alteration or commercial use, including those for text and data mining, AI training, and similar technologies.

Study (Year)	CVD -Absolute/Relative risk	CVD -Incident risk	CHD -Absolute / Relative risk	CHD -Incident risk
Poorthuis et al,[9] 2022	NR	Incident risk (HR, 95% CI):<br>Overall:<br>Hysterectomy + BSO: 1.18 (1.11-1.25)<br>Hysterectomy-only: 1.09 (1.06-1.12)<br>No surgery: 1.00 (0.99-1.01)<br>Age-stratified<br><48 y:<br>Hysterectomy + BSO: 1.21 (1.13-1.30)<br>Hysterectomy-only: 1.10 (1.06-1.13)<br>≥48 y:<br>Hysterectomy + BSO: 1.13 (1.01-1.28)<br>Hysterectomy-only: 1.07 (1.02-1.13)	NR	Incident risk (HR, 95% CI):<br>Overall:<br>Hysterectomy + BSO: 1.17 (1.07-1.27)<br>Hysterectomy-only: 1.11 (1.07-1.16)<br>No surgery: 1.00 (0.99-1.01)<br><br>Age-stratified<br><br><48 y:<br>Hysterectomy + BSO: 1.15 (1.04-1.28)<br>Hysterectomy-only: 1.12 (1.07-1.17)<br>≥48 y:<br>Hysterectomy + BSO: 1.25 (1.07-1.46)<br>Hysterectomy-only: 1.11 (1.03-1.19)
Thong et al,[24] 2022	NR	NR	Cases vs comparators, Relative risk (IRR, 95% CI):<br>IHD: 0.66 (0.48-0.88)	NR
Zhu et al,[15] 2020	NR	BSO±hysterectomy vs. Natural menopause, Incident risk (HR, 95% CI):<br><br>Overall: 1.22 (1.16-1.28)<br><br>Age-stratified<br><35 y:<br>2.55 (2.22-2.94) vs. 1.59 (1.23-2.05)<br>35-39 y:<br>1.91 (1.71-2.14) vs. 1.51 (1.33-1.72)	NR	BSO±hysterectomy vs. Natural menopause, Incident risk (HR, 95% CI):<br><br>Overall: 1.26 (1.19-1.33)<br><br>Age-stratified<br><35 y:<br>2.55 (2.17-2.99) vs. 1.59 (1.18-2.13)<br>35-39 y:<br>1.92 (1.69-2.19) vs. 1.49 (1.28-1.73)

© 2026 Armeni E. *JAMA Network Open*. This is an open access article distributed under the terms of the CC-BY-NC-ND License, which does not permit alteration or commercial use, including those for text and data mining, AI training, and similar technologies.

Study (Year)	CVD -Absolute/Relative risk	CVD -Incident risk	CHD -Absolute / Relative risk	CHD -Incident risk
		40-44 y:<br>1.58 (1.44-1.74) vs. 1.32 (1.24-1.41)<br>45-49 y:<br>1.20 (1.10-1.31) vs. 1.13 (1.08-1.18)<br>50-54 y:<br>0.91 (0.82-1.01) vs. Reference<br>≥ 55 y:<br>0.73 (0.61-0.87) vs. 0.97 (0.92-1.02)		40-44 y:<br>1.63 (1.46-1.81) vs. 1.32 (1.23-1.43)<br>45-49 y:<br>1.23 (1.11-1.36) vs. 1.13 (1.07-1.20)<br>50-54 y:<br>0.92 (0.81-1.05) vs. Reference<br>≥ 55 y:<br>0.76 (0.62-0.93) vs. 0.96 (0.90-1.03)

Abbreviations: ABI, ankle-brachial index; baPWV, brachial-ankle pulse wave velocity; BRCA, breast cancer susceptibility gene; BSO, bilateral salpingo-oophorectomy; CAC, coronary artery calcium; cAP, central aortic augmentation pressure; cDBP, central diastolic blood pressure; cfPWV, carotid-femoral pulse wave velocity; CHD, coronary heart disease; CI, confidence interval; CIMT, carotid intima-media thickness; cPP, central aortic pulse pressure; cSBP, central systolic blood pressure; CVD, cardiovascular disease; HR, hazard ratio; IHD, ischemic heart disease; IMT, intima-media thickness; IRR, incidence rate ratio; MI, myocardial infarction; NR, not reported; OR, odds ratio; POI, premature ovarian insufficiency; SD, standard deviation; SE, standard error; y, years.

© 2026 Armeni E. *JAMA Network Open*. This is an open access article distributed under the terms of the CC-BY-NC-ND License, which does not permit alteration or commercial use, including those for text and data mining, AI training, and similar technologies.

eTable 5. Studies reporting on absolute, relative, and incident risk for stroke and metabolic syndrome morbidity

Study (Year)	Stroke - Absolute/Relative risk	Stroke -Incident risk	Metabolic Syndrome - Absolute / Relative risk	Metabolic Syndrome - Incident risk
Dorum et al,[27] 2008	NR	Prevalent, self reported<br>Cases vs. comparators (n/N):<br>CVD events:<br>8/263 vs. 7/789	Cases vs. comparators (n/N):<br>NCEP criteria:<br>101/263 vs. 238/789<br><br>IDF criteria:<br>123/263 vs. 282/789<br><br>OR (95% CI) 1.58 (1.19-2.1)	NR
Do Valle et al,[19] 2021	Absolute risk (n/N, %):<br>BRCA+BSO:<br>12/360, 3.3%<br>BSO (benign, no mutation):<br>173/3,600, 4.8%<br>Intact ovaries, no mutation:<br>128/3,600, 3.6%	NR	NR	NR
Farahmand et al,[21] 2014	NR	NR	Cases vs. comparators:<br>IDF Criteria:<br>Absolute risk (%, N):<br>32.5%, 40 vs. 28.6%, 178<br>Relative risk OR (95% CI):<br>1.2 (0.6-2.5)	NR
Farland et al,[11] 2023	Absolute risk (n/N):<br>Hysterectomy+BSO: 158/2,153<br>Hysterectomy-only:<br>67/2,960<br>No surgery:<br>541/108,448	Incident risk (HR, 95% CI):<br>Hysterectomy+BSO vs. no surgery: 1.45 (1.21-1.75)<br>Hysterectomy-only vs. no surgery:<br>1.19 (0.92-1.54)	NR	NR
Honigberg et al,[12] 2019	Ischemic stroke only<br><br>BSO vs. natural menopause, Relative risk (IRR, 95% CI):<br><br>0.67 (0.24-1.61) vs. 0.94 (0.67-1.29)	Ischemic stroke only<br><br>BSO <40 y vs. POI, Incident risk (HR, 95% CI):<br>1.18 (0.38-3.66) vs. 1.59 (1.12-2.28)<br><br>Surgical menopause vs. natural menopause, Incident risk (HR, 95% CI):<br><30 y: NA vs. 1.3 (0.3-5.2)<br><35 y: 1.0 (0.1-7.1) vs. 1.8 (1.0-3.4)	NR	NR

© 2026 Armeni E. *JAMA Network Open*. This is an open access article distributed under the terms of the CC-BY-NC-ND License, which does not permit alteration or commercial use, including those for text and data mining, AI training, and similar technologies.

Study (Year)	Stroke - Absolute/Relative risk	Stroke -Incident risk	Metabolic Syndrome - Absolute / Relative risk	Metabolic Syndrome - Incident risk
		<40 y: 1.3 (0.4-4.0) vs. 1.7 (1.2-2.5)<br><45 y: 1.9 (1.1-3.2) vs. 1.3 (1.0-1.7)<br><50 y: 1.4 (0.9-2.1) vs. 1.2 (1.0-1.4)<br>≥50 y: Reference		
Howard et al,[22] 2005	Absolute risk (n/N) and annual incidence rate (%):<br>Hysterectomy+BSO : 208/18,251, annual 0.23%<br>Hysterectomy only: 198/18,687, annual 0.21%;<br>No surgery: 509/52,976, annual 0.19	NR	NR	NR
Indhavivadhana et al,[28] 2011	NR	NR	Cases vs. Comparators: NCEP Criteria:<br><u>Absolute risk (%, N):</u> 19.7%, 198) vs. 16.9%, 442<br><u>Relative risk (OR, 95% CI):</u> 0.9 (0.7-1.11)	NR
Ingelsson et al,[1] 2010	No surgery vs. hysterectomy+BSO vs. BSO-only vs. Hysterectomy-only, Absolute risk (n/N):<br><br>≤ 49y:<br>No surgery: 6,565/359,363<br>Hysterectomy+BSO : 174/8,347<br>BSO-only: 56/2,118<br>Hysterectomy-only: 2,275/104,770<br><br>≥50 y:<br>No surgery: 16,313/284,652<br>Hysterectomy+BSO : 791/19,789<br>BSO-only: 158/3,000<br>Hysterectomy-only: 2823/51,535	No surgery vs. hysterectomy vs. oophorectomy Incident risk (HR, 95% CI):<br><u>Age stratified:</u><br>≤ 49y: 1 vs. 1.19 (1.13-1.25) vs. 1.33 (1.08-1.64)<br>≥50 y: 1 vs. 1.00 (0.96-1.04) vs. 1.06 (0.84-1.34)	NR	NR
Netterlid et al,[31] 2021	NR	NR	OR 7.7 (95% CI 2.1–28.1) for MetS in childhood cancer survivors with POI vs controls	23% (5/22) in CCS with POI vs 4% (6/164) in controls

© 2026 Armeni E. *JAMA Network Open*. This is an open access article distributed under the terms of the CC-BY-NC-ND License, which does not permit alteration or commercial use, including those for text and data mining, AI training, and similar technologies.

Study (Year)	Stroke - Absolute/Relative risk	Stroke -Incident risk	Metabolic Syndrome - Absolute / Relative risk	Metabolic Syndrome - Incident risk
Ou et al,[32] 2023	NR	NR	BSO ± hysterectomy vs. Age-matched controls:<br><u>Absolute risk (%, n/N):</u><br>33%, 642/1,967 vs. 30%, 2,812/9,441<br><br><u>Relative risk (OR, 95% CI):</u><br>1.38 (1.19-1.6) vs 1.11 (0.97-1.26)	NR
Ozdemir et al,[33] 2009	NR	NR	Cases vs. Comparators (%, n/N)<br>47.8%, 45/94 vs. 40%, 38/95	NR
Poorthuis et al,[9] 2022	NR	Incident risk (HR, 95% CI):<br><u>Overall:</u><br>Hysterectomy + BSO: 1.18 (1.08-1.28)<br>Hysterectomy-only: 1.03 (0.99-1.08)<br>No surgery: 1.00 (0.99-1.01)<br><br><u>Age-stratified</u><br><48 y:<br>Hysterectomy + BSO: 1.23 (1.12-1.36)<br>Hysterectomy-only: 1.06 (1.01-1.11)<br><br>≥48 y:<br>Hysterectomy + BSO: 1.07 (0.91-1.26)<br>Hysterectomy-only: 0.99 (0.92-1.07)	NR	NR
Thong et al,[24] 2022	(Combined Morbidity and mortality risk)<br>IRR (95% CI): 0.56 (0.27-1.03)	NR	NR	NR
Tuysuzoglu et al,[35] 2020	NR	NR	Cases vs. Comparators (%, n/N)<br>40%, 14/35 vs. 17.14%, 6/35	NR
Zhu et al,[15] 2020	NR	BSO±hysterectomy vs. Natural menopause, Incident risk (HR, 95% CI):<br><u>Overall:</u> 1.21 (1.11-1.31)<br><br><u>Age-stratified</u><br><35 y:	NR	NR

© 2026 Armeni E. *JAMA Network Open*. This is an open access article distributed under the terms of the CC-BY-NC-ND License, which does not permit alteration or commercial use, including those for text and data mining, AI training, and similar technologies.

Study (Year)	Stroke - Absolute/Relative risk	Stroke -Incident risk	Metabolic Syndrome - Absolute / Relative risk	Metabolic Syndrome - Incident risk
		2.60 (2.03-3.33) vs. 1.41 (0.87-2.27)<br>35-39 y:<br>1.91 (1.56-2.33) vs. 1.77 (1.43-2.18)<br>40-44 y:<br>1.54 (1.30-1.82) vs. 1.31 (1.17-1.47)<br>45-49 y:<br>1.21 (1.04-1.41) vs. 1.11 (1.03-1.20)<br>50-54 y:<br>0.93 (0.78-1.12) vs. Reference<br>≥ 55 y:<br>0.61 (0.44-0.85) vs. 0.98 (0.89-1.08)		

Abbreviations: BRCA, breast cancer susceptibility gene; BSO, bilateral salpingo-oophorectomy; CCS, childhood cancer survivors; CI, confidence interval; CVD, cardiovascular disease; HR, hazard ratio; IDF, International Diabetes Federation; IRR, incidence rate ratio; MetS, metabolic syndrome; NA, not applicable; NCEP, National Cholesterol Education Program; NR, not reported; OR, odds ratio; POI, premature ovarian insufficiency; y, years.

© 2026 Armeni E. *JAMA Network Open*. This is an open access article distributed under the terms of the CC-BY-NC-ND License, which does not permit alteration or commercial use, including those for text and data mining, AI training, and similar technologies.

eTable 6. Studies reporting on absolute, relative, and incident risk for congestive heart failure.

Study (Year)	CHF -Absolute / Relative risk	CHF -Incident risk
Honigberg et al,[12] 2019	BSO vs. natural menopause, Relative risk (IRR, 95% CI):<br>2.02 (1.08-3.54) vs. 1.2 (0.89-1.6)	BSO <40 y vs. POI, Incident risk (HR, 95% CI):<br>2.74 (1.42-5.29) vs. 1.56 (1.14-2.16)<br><br>Surgical menopause vs. natural menopause, Incident risk (HR, 95% CI):<br><30 y: 5.3 (1.3-21.7) vs 1.1 (0.3-4.3)<br><35 y: 3.4 (1.3-9.1) vs 1.1 (0.5-2.3)<br><40 y: 3.1 (1.6-6.0) vs 1.8 (1.3-2.5)<br><45 y: 1.8 (1.1-2.9) vs 1.4 (1.2-1.8)<br><50 y: 1.7 (1.2-2.4) vs 1.5 (1.3-1.7)<br>≥50 y: Reference
Howard et al,[22] 2005 (WHI-OS)	(n/N, annual incidence %):<br>Hysterectomy+BSO: 263/18,251, annual 0.29%<br>Hysterectomy only: 255/18,687, annual 0.28%<br>No surgery: 548/52,976, annual 0.21%	NR

Abbreviations: BSO, bilateral salpingo-oophorectomy; CHF, congestive heart failure; CI, confidence interval; HR, hazard ratio; IRR, incidence rate ratio; NR, not reported; POI, premature ovarian insufficiency; WHI-OS, Women's Health Initiative Observational Study; y, years.

© 2026 Armeni E. *JAMA Network Open*. This is an open access article distributed under the terms of the CC-BY-NC-ND License, which does not permit alteration or commercial use, including those for text and data mining, AI training, and similar technologies.

eTable 7. Studies reporting on absolute, relative, and incident risk for cardiovascular disease mortality

Study (Year)	CVD Mortality -Absolute / Relative Risk (RR, 95% CI)	CVD Mortality -Incident Risk
Appiah et al,[5] 2015	CVD Mortality, Absolute risk (n/N):<br><br><u>Overall:</u><br>Cases (BSO, no diabetes): 261/1,340<br>Cases (BSO, diabetes): 35/101<br>Comparators (diabetes, no BSO): 132/401<br>Comparators (no BSO, no diabetes): 1,209/6,135<br><br><u>Age at menopause:</u><br>Diabetes<br>No BSO ≤ 45 y: 44/133<br>BSO ≤ 45 y: 17/47<br>No BSO >45 y: 88/268<br>BSO >45 y: 18/54<br><br>No diabetes<br>No BSO ≤ 45 y: 389/1,838<br>BSO ≤ 45 y: 102/488<br>No BSO >45 y: 820/4,297<br>BSO >45 y: 159/852	CVD Mortality, Incident risk (HR, 95% CI):<br><br><u>Overall:</u><br>Cases (BSO, no diabetes): 1.11 (0.97-1.27)<br>Cases (BSO, diabetes): 3.20 (2.28-4.48)<br>Comparators (diabetes, no BSO): 2.33 (1.95-2.79)<br>Comparators (no BSO, no diabetes): 1<br><br><u>Age at menopause:</u><br>Diabetes<br>No BSO ≤ 45 y: 1<br>BSO ≤ 45 y: 1.60 (0.91-2.81)<br>No BSO >45 y: 1<br>BSO >45 y: 1.19 (0.72-1.98)<br><br>No diabetes<br>No BSO ≤ 45 y: 1<br>BSO ≤ 45 y: 1.12 (0.90-1.38)<br>No BSO >45 y: 1<br>BSO >45 y: 1.10 (0.93-1.30)
Appiah et al,[4] 2020	Heart Disease Mortality, Absolute risk (n/N):<br><br><u>Overall:</u><br>No FHPMI, no BSO: 288/1915<br>FHPMI, no BSO: 29/243<br>BSO, no FHPMI: 61/515<br>BSO, FHPMI: 17/95<br><br><u>Age stratified:</u><br><45 y:<br>No FHPMI, no BSO: 96/652<br>FHPMI, no BSO: 11/96<br>BSO, no FHPMI: 31/290<br>BSO, FHPMI: 11/66<br><br>≥ 45 y:<br>No FHPMI, no BSO: 192/1263<br>FHPMI, no BSO: 18/147<br>BSO, no FHPMI: 30/225<br>BSO, FHPMI: 6/29<br><br>CVD Mortality, Absolute risk (n/N):<br><br><u>Overall:</u><br>No FHPMI, no BSO: 401/1915<br>FHPMI, no BSO: 37/243<br>BSO, no FHPMI: 87/515<br>BSO, FHPMI: 17/95<br><br><u>Age stratified:</u><br><45 y:<br>No FHPMI, no BSO: 124/652<br>FHPMI, no BSO: 13/96	Heart Disease Mortality, Incident risk (HR, 95% CI):<br><br><u>Overall:</u><br>No FHPMI, no BSO: 1<br>FHPMI, no BSO: 1.14 (0.78-1.69)<br>BSO, no FHPMI: 0.97 (0.74-1.27)<br>BSO, FHPMI: 2.36 (1.46-3.82)<br><br><u>Age stratified (BSO, FHPMI):</u><br><45 y vs. ≥ 45 y:<br>4.32 (1.95-9.50) vs 1.60 (0.63-4.09)<br><br>CVD Mortality, Incident risk (HR, 95% CI):<br><br><u>Overall:</u><br>No FHPMI, no BSO: 1<br>FHPMI, no BSO: 1.01 (0.73-1.41)<br>BSO, no FHPMI: 0.95 (0.76-1.20)<br>BSO, FHPMI: 1.63 (1.01-2.63)<br><br><u>Age stratified (BSO, FHPMI):</u><br><45 y vs. ≥ 45 y:<br>3.21 (1.52-6.80) vs. 1.06 (0.42-2.66)

© 2026 Armeni E. *JAMA Network Open*. This is an open access article distributed under the terms of the CC-BY-NC-ND License, which does not permit alteration or commercial use, including those for text and data mining, AI training, and similar technologies.

Study (Year)	CVD Mortality -Absolute / Relative Risk (RR, 95% CI)	CVD Mortality -Incident Risk
	BSO, no FHPMI: 45/290<br>BSO, FHPMI: 11/66<br><br>≥ 45 y:<br>No FHPMI, no BSO: 277/1263<br>FHPMI, no BSO: 24/147<br>BSO, no FHPMI: 42/225<br>BSO, FHPMI: 6/29	
Duan et al,[20] 2012	Surgical vs. natural menopause, Absolute (n/N) and relative risk RR, 95% CI:<br><br><u>Overall</u>:<br>338/9,785 vs. 1,729/32,219 and 0.86 (0.76-0.97)<br><br><u>Age at menopause</u>,<br>≥ 45 y:<br>202/6,031 vs. 1,572/29,748 and 0.83 (0.71-0.96)<br>< 45 y:<br>136/3,763 vs 157/2,480 and 0.85 (0.66-1.11)	NR
Gierach et al,[10] 2014	CHD Mortality, Absolute risk (n/N):<br><br><u>Age-stratified</u><br>≤ 35y:<br>BSO: 114/989<br>Hysterectomy-only: 131/2,104<br>No surgery: 3,208/49,753<br><br>≤ 40y:<br>BSO: 214/2,270<br>Hysterectomy-only: 276/4,927<br>No surgery: 2963/45,649<br><br>≤ 45y:<br>BSO: 371/4,715<br>Hysterectomy-only: 455/7,865<br>No surgery: 2,626/40,256<br><br>≤ 50 y:<br>BSO: 567/7,998<br>Hysterectomy-only: 600/9,840<br>No surgery: 2,280/34,918<br><br>≤ 55y:<br>BSO: 684/10,275<br>Hysterectomy-only: 642/10,448<br>No surgery: 2086/31,778<br><br>Stroke Mortality, Absolute risk (n/N):<br><br>≤ 35y:<br>BSO: 22/989<br>Hysterectomy-only: 32/2,104<br>No surgery: 1,123/49,753<br><br>≤ 40y:<br>BSO: 64/2,270<br>Hysterectomy-only: 93/4,927	CHD Mortality, Incident risk (HR, 95%CI):<br><br><u>Age at menopause, age-matched</u><br>≤ 35y:<br>BSO: 1.56 (1.29-1.89)<br>Hysterectomy-only: 1.21 (1.02-1.44)<br>No surgery: Reference<br><br>≤ 40y:<br>BSO: 1.37 (1.19-1.58)<br>Hysterectomy-only: 1.13 (0.99-1.28)<br>No surgery: Reference<br><br>≤ 45y:<br>BSO: 1.28 (1.14-1.43)<br>Hysterectomy-only: 1.10 (0.99-1.22)<br>No surgery: Reference<br><br>≤ 50 y:<br>BSO: 1.20 (1.08-1.32)<br>Hysterectomy-only: 1.11 (1.01-1.22)<br>No surgery: Reference<br><br>≤ 55y:<br>BSO: 1.10 (1.00-1.21)<br>Hysterectomy-only: 1.07 (0.97-1.17)<br>No surgery: Reference<br><br>Stroke Mortality, Incident risk (HR, 95%CI):<br><br>≤ 35y:<br>BSO: 0.84 (0.55-1.29)<br>Hysterectomy-only: 0.85 (0.59-1.20)<br>No surgery: Reference<br><br>≤ 40y:<br>BSO: 1.15 (0.89-1.48)<br>Hysterectomy-only: 1.09 (0.88-1.36)

© 2026 Armeni E. *JAMA Network Open*. This is an open access article distributed under the terms of the CC-BY-NC-ND License, which does not permit alteration or commercial use, including those for text and data mining, AI training, and similar technologies.

Study (Year)	CVD Mortality -Absolute / Relative Risk (RR, 95% CI)	CVD Mortality -Incident Risk
	No surgery:1,020/45,649<br><br>≤ 45y:<br>BSO: 126/4,715<br>Hysterectomy-only: 148/7,865<br>No surgery: 901/40,256<br><br>≤ 50 y:<br>BSO: 197/7,998<br>Hysterectomy-only: 193/9,840<br>No surgery: 781/34,918<br><br>≤ 55y:<br>BSO: 253/10,275<br>Hysterectomy-only: 214/10,448<br>No surgery: 687/31,778	No surgery: Reference<br><br>≤ 45y:<br>BSO: 1.19 (0.98-1.45)<br>Hysterectomy-only: 1.01 (0.85-1.21)<br>No surgery: Reference<br><br>≤ 50 y:<br>BSO: 1.12 (0.95-1.33)<br>Hysterectomy-only: 0.99 (0.84-1.17)<br>No surgery: Reference<br><br>≤ 55y:<br>BSO: 1.14 (0.97-1.34)<br>Hysterectomy-only: 1.02 (0.86-1.20)<br>No surgery: Reference
Howard et al,[22] 2005	CVD+CHD mortality, Absolute risk (n/N), annual incidence (%):<br>Hysterectomy+BSO: 135/18,251, annual 0.16%<br>Hysterectomy-only: 98/18,687, annual 0.11%<br>No surgery: 295/52,976, annual 0.11%<br><br>CHD mortality, Absolute risk (n/N), annual incidence (%):<br>Hysterectomy+BSO: 57/18,251, annual 0.07%<br>Hysterectomy-only: 40/18,687, annual 0.04%<br>No surgery: 118/52,976, annual 0.05%	NR
Michelsen et al,[8] 2023	Absolute risk (n/N):<br>Overall:<br>BSO ± Hysterectomy: 207/907<br>Hysterectomy-only: 186/1,199<br>No oophorectomy: 498/18,673<br><br>Age-stratified<br><40y:<br>BSO ± Hysterectomy: 32/207<br>Hysterectomy-only: 24/297<br>40-52y:<br>BSO ± Hysterectomy: 72/420<br>Hysterectomy-only: 110/761<br>≥53y:<br>BSO ± Hysterectomy: BSO: 103/280<br>Hysterectomy-only: 52/141	Incident risk (HR, 95% CI):<br>Overall:<br>BSO ± Hysterectomy vs. no oophorectomy: 1.15 (0.95-1.40)<br>Hysterectomy-only vs. no oophorectomy: 1.23 (1.02-1.49)<br><br>Age-stratified<br><40y:<br>BSO ± Hysterectomy vs. no oophorectomy: 1.20 (0.83-1.74)<br>Hysterectomy-only vs. no oophorectomy: 1.28 (0.85-1.94)<br><br>40-52y:<br>BSO ± Hysterectomy vs. no oophorectomy: 0.95 (0.73-1.24)<br>Hysterectomy-only vs. no oophorectomy: 1.21 (0.97-1.51)<br><br>≥53y:<br>BSO ± Hysterectomy vs. no oophorectomy: 1.37 (1.08-1.74)<br>Hysterectomy-only vs. no oophorectomy: 1.30 (0.95-1.78)

© 2026 Armeni E. *JAMA Network Open*. This is an open access article distributed under the terms of the CC-BY-NC-ND License, which does not permit alteration or commercial use, including those for text and data mining, AI training, and similar technologies.

Study (Year)	CVD Mortality -Absolute / Relative Risk (RR, 95% CI)	CVD Mortality -Incident Risk
Olesen et al,[14] 2022	BSO ± hysterectomy vs. age-adjusted controls without iatrogenic menopause, Absolute risk (n/N): 50/2,040 vs. 707/22,358	BSO ± hysterectomy vs. age-adjusted controls without iatrogenic menopause, Incident risk (MRR, 95% CI):<br>Overall: 1.07 (0.8-1.42)<br><br>Stratified by hysterectomy status:<br>BSO-only vs. no surgery: 1.68 (0.84-3.38)<br>BSO+hysterectomy vs. hysterectomy-only: 1.29 (0.79-2.12)
Rivera et al,[7] 2009	BSO vs. age-adjusted controls without iatrogenic menopause, Absolute risk (n/N):<br>Overall:<br>106/1,091 vs. 216/2,383<br><br>Age-stratified<br>< 45 y: 26/413 vs. 68/1,417<br>45-50 y: 39/430 vs. 88/645<br>> 50 y: 41/248 vs. 60/321	BSO vs. age-adjusted controls without iatrogenic menopause, Relative risk (HR, 95% CI):<br>Overall: 0.87 (0.69-1.1)<br><br>Age-stratified<br><45 y: 1.25 (0.79-1.96)<br>45-50 y: 0.72 (0.49-1.05)<br>>50 y: 0.86 (0.58-1.29)
Rocca et al,[6] 2006	BSO vs. age-adjusted controls without iatrogenic menopause, Absolute risk (n/N):<br>- Cardiovascular: 34/1,091 vs. 149/2383-Cerebrovascular 9/1,091 vs. 41/2383<br>- Arterial and venous thromboembolism, 13/1,091 vs. 30/2383	BSO vs. age-adjusted controls without iatrogenic menopause, Incident risk (HR, 95% CI):<br>0.75 (0.52-1.1)
Thong et al,[24] 2022	BSO vs. age-adjusted controls without iatrogenic menopause, Absolute risk (n/N) and Relative risk (IRR, 95% CI):<br>Overall: 7/45 vs. 132/591<br>Grouped by condition:<br>- Circulatory disorders: 0.62 (0.53-0.72)<br>- IHD: 0.66 (0.48-0.88)<br>- Cerebrovascular disease: 0.56 (0.27-1.03)	NR
Tuesley et al,[25] 2020	Hysterectomy + BSO vs. no surgery, Absolute risk (n/N):<br><br>Overall: 235/18,558 vs. 5,229/553,958<br><br>Age-stratified:<br>< 35 y: 11/1,014<br>35-44 y: 63/4,936<br>45-54 y: 91/8,599<br>55-64 y: 53/2,963<br>≥ 65 y: 17/1,046	Hysterectomy + BSO vs. no surgery, Incident risk (HR, 95% CI):<br><br>Overall: 0.96 (0.84-1.1)<br><br>Age-stratified:<br>< 35 y: 1.67 (0.92-3.02)<br>35-44 y: 1.22 (0.95-1.56)<br>45-54 y: 0.91 (0.74-1.12)<br>55-64 y: 0.89 (0.68-1.17)<br>≥ 65 y: 0.62 (0.39-1)
Xu et al,[3] 2022	Absolute risk (n/N):<br><br>Age-stratified<br>< 40 y:<br>BSO: 19/2,534<br>Hysterectomy-only: 87/9,054<br>Natural menopause: 35/2,548<br>40-44 y:<br>BSO: 22/3652<br>Hysterectomy-only: 63/7,953<br>Natural menopause: 81/10,045<br>45-49 y:<br>BSO: 29/6,064<br>Hysterectomy-only: 20/4,985<br>Natural menopause: 234/32,274	Incident risk (HR, 95% CI):<br><br>Age-stratified<br>< 40 y:<br>BSO: 1.78 (1.12-2.84)<br>Hysterectomy-only: 1.95 (1.53-2.48)<br>Naturally menopause: 3.18 (2.23-4.52)<br>40-44 y:<br>BSO: 1.55 (1.01-2.40)<br>Hysterectomy-only: 1.40 (1.06-1.85)<br>Natural menopause: 1.59 (1.24-2.04)<br>45-49 y:<br>BSO: 1.03 (0.70-1.50)<br>Hysterectomy-only: 0.64 (0.41-1.01)<br>Natural menopause: 1.54 (1.29-1.84)

© 2026 Armeni E. *JAMA Network Open*. This is an open access article distributed under the terms of the CC-BY-NC-ND License, which does not permit alteration or commercial use, including those for text and data mining, AI training, and similar technologies.

Study (Year)	CVD Mortality -Absolute / Relative Risk (RR, 95% CI)	CVD Mortality -Incident Risk
	50-52 y:<br>BSO: 24/3,405<br>Hysterectomy-only: 8/1,269<br>Natural menopause: 261/50,345<br>≥ 53 y:<br>BSO: 5/1914<br>Hysterectomy-only: 2/759<br>Natural menopause: 235/41,578	50-52 y:<br>BSO: 1.16 (0.77-1.77)<br>Hysterectomy-only: 1.02 (0.50-2.05)<br>Natural menopause (Reference)<br>≥ 53 y:<br>BSO: 0.40 (0.17-0.97)<br>Hysterectomy-only: 0.42 (0.10-1.67)<br>Natural menopause: 1.00 (0.83-1.19)

Abbreviations: BSO, bilateral salpingo-oophorectomy; CHD, coronary heart disease; CI, confidence interval; CVD, cardiovascular disease; FHPMI, family history of premature myocardial infarction; HR, hazard ratio; IHD, ischemic heart disease; IRR, incidence rate ratio; MRR, mortality rate ratio; NR, not reported; RR, risk ratio; y, years.

© 2026 Armeni E. *JAMA Network Open*. This is an open access article distributed under the terms of the CC-BY-NC-ND License, which does not permit alteration or commercial use, including those for text and data mining, AI training, and similar technologies.

eTable 8. Studies reporting on absolute, relative, and incident risk for all-cause mortality.

Study (Year)	All-Cause Mortality -Absolute (%, n/N) / Relative Risk (RR, 95% CI)	All-Cause Mortality -Incident Risk (HR or MRR, 95% CI)
Abildgaard et al,[16] 2018	Absolute risk (%, n/N):<br>Age-stratified<br>≤45 y: 0.3%, 1/302<br>> 45 y: 1.6%, 18/1,136	Oophorectomy vs. age-matched population control, Incident Risk (MRR, 95% CI):<br>Age-stratified<br>≤45 y: 0.56 (0.07-4.22)<br>> 45 y: 0.7 (0.44-1.14)
Appiah et al,[5] 2015	Absolute risk (n/N):<br><br>Overall:<br>Cases (BSO, no diabetes): 776/1,340<br>Cases (BSO, diabetes): 78/101<br>Comparators (diabetes, no BSO): 311/401<br>Comparators (no BSO, no diabetes): 3,632/6,135<br><br>Age at menopause:<br>Diabetes<br>No BSO ≤ 45 y: 100/133<br>BSO ≤ 45 y: 35/47<br>No BSO >45 y: 211/268<br>BSO >45 y: 43/54<br><br>No diabetes<br>No BSO ≤ 45 y: 1,131/1,838<br>BSO ≤ 45 y: 284/488<br>No BSO >45 y: 2,501/4,297<br>BSO >45 y: 492/852	Incident risk (HR, 95% CI):<br><br>Overall:<br>Cases (BSO, no diabetes): 1.10 (1.02-1.19)<br>Cases (BSO, diabetes): 2.36 (1.88-2.95)<br>Comparators (diabetes, no BSO): 1.83 (1.63-2.06)<br>Comparators (no BSO, no diabetes): 1<br><br>Age at menopause:<br>Diabetes<br>No BSO ≤ 45 y: 1<br>BSO ≤ 45 y: 1.45 (0.98-2.14)<br>No BSO >45 y: 1<br>BSO >45 y: 1.20 (0.86-1.66)<br><br>No diabetes<br>No BSO ≤ 45 y: 1<br>BSO ≤ 45 y: 1.07 (0.94-1.22)<br>No BSO >45 y: 1<br>BSO >45 y: 1.10 (1.00-1.22)
Appiah et al,[4] 2020	Absolute risk (n/N):<br><br>Overall:<br>No FHPMI, no BSO: 1,223/1,915<br>FHPMI, no BSO: 143/243<br>BSO, no FHPMI: 285/515<br>BSO, FHPMI: 62/95<br><br>Age stratified:<br><45 y:<br>No FHPMI, no BSO: 378/652<br>FHPMI, no BSO: 47/96<br>BSO, no FHPMI: 152/290<br>BSO, FHPMI: 42/66<br><br>≥ 45 y:<br>No FHPMI, no BSO: 845/1263<br>FHPMI, no BSO: 96/147<br>BSO, no FHPMI: 133/225<br>BSO, FHPMI: 20/29	Incident risk (HR, 95% CI):<br><br>No FHPMI, no BSO: 1<br>FHPMI, no BSO: 1.32 (1.12-1.57)<br>BSO, no FHPMI: 1.00 (0.90-1.12)<br>BSO, FHPMI: 1.83 (1.49-2.25)<br><br>Age stratified (BSO, FHPMI):<br><45 y vs. ≥ 45 y:<br>1.75 (1.28-2.41) vs. 1.31 (0.89- 1.93)
Duan et al,[20] 2012	BSO vs. natural menopause, Absolute risk (n/N) and Relative risk (RR, 95% CI):<br>Overall:<br>1,028/9,785 vs. 5,004/32,219 and 0.87 (0.81-0.94)<br><br>Age at menopause:<br>≥ 45 y: 0.87 (0.80-0.94)	NR

© 2026 Armeni E. *JAMA Network Open*. This is an open access article distributed under the terms of the CC-BY-NC-ND License, which does not permit alteration or commercial use, including those for text and data mining, AI training, and similar technologies.

Study (Year)	All-Cause Mortality -Absolute (%, n/N) / Relative Risk (RR, 95% CI)	All-Cause Mortality -Incident Risk (HR or MRR, 95% CI)
	< 45 y: 0.86 (0.74-1.00)	
Gierach et al,[10] 2014	Absolute risk (n/N):<br><br>Overall:<br>BSO: 3,293/12,652<br>Hysterectomy-only: 2,848/11,247<br>No surgery: 7,593/28,947<br>Age-stratified, age-matched (n of events)<br>≤ 35 y:<br>- BSO: 342<br>- Hysterectomy-only: 497<br>36-40 y:<br>- BSO: 349<br>- Hysterectomy-only: 649<br>41-45 y:<br>- BSO: 610<br>- Hysterectomy-only: 741<br>46-50 y:<br>- BSO: 779<br>- Hysterectomy-only: 576<br>≥51 y:<br>- BSO: 1,213<br>- Hysterectomy-only: 583	Incident risk (HR, 95% CI):<br><br>Overall:<br>BSO: 0.99 (0.95-1.04)<br>Hysterectomy-only: 1.01 (0.96-1.05)<br>No surgery: Reference<br><br>Age at menopause, age-matched<br>≤ 35 y:<br>BSO: 1.19 (1.07-1.33)<br>Hysterectomy-only: 1.09 (0.99-1.19)<br>36-40 y:<br>BSO: 1.03 (0.92-1.15)<br>Hysterectomy-only: 1.05 (0.96-1.13)<br>41-45 y:<br>BSO: 1.04 (0.96-1.14)<br>Hysterectomy-only: 0.98 (0.90-1.05)<br>46-50 y:<br>BSO: 0.96 (0.89-1.03)<br>Hysterectomy-only: 0.97 (0.89-1.06)<br>≥51 y:<br>BSO: 0.94 (0.89-1.00)<br>Hysterectomy-only: 0.98 (0.90-1.07)
Michelsen et al,[8] 2023	Absolute risk (n/N):<br>Overall:<br>BSO ± Hysterectomy: 362/907<br>Hysterectomy-only: 335/1,199<br>No oophorectomy: 1,300/18,673<br><br>Age-stratified<br><40y:<br>BSO ± Hysterectomy: 59/207<br>Hysterectomy-only: 46/297<br>40-52y:<br>BSO ± Hysterectomy: 141/420<br>Hysterectomy-only: 204 /761<br>≥53y:<br>BSO ± Hysterectomy: 162/280<br>Hysterectomy-only: 85/141	Incident risk (HR, 95% CI):<br>Overall:<br>BSO ± Hysterectomy vs. no oophorectomy: 1.24 (1.09-1.42)<br>Hysterectomy-only vs. no oophorectomy: 1.26 (1.11-1.44)<br><br>Age-stratified<br><40y:<br>BSO ± Hysterectomy vs. no oophorectomy: 1.31 (1.0-1.71)<br>Hysterectomy-only vs. no oophorectomy: 1.18 (0.88-1.59)<br><br>40-52y:<br>BSO ± Hysterectomy vs. no oophorectomy: 1.11 (0.92-1.33)<br>Hysterectomy-only vs. no oophorectomy: 1.22 (1.05-1.43)<br><br>≥53y:<br>BSO ± Hysterectomy vs. no oophorectomy: 1.40 (1.18-1.68)<br>Hysterectomy-only vs. no oophorectomy: 1.49 (1.18-1.88)
Rocca et al,[6] 2006	BSO vs. age-adjusted controls without iatrogenic menopause, Absolute risk (n/N):<br><br>Overall:<br>360/1,091 vs. 628/2,383<br><br>Age-stratified<br><45 y: 33/124 vs. 229/1,417<br>45-50 y: 75/243 vs. 240/645	BSO vs. age-adjusted controls without iatrogenic menopause, Incident risk (HR, 95% CI):<br>Overall: 1.05 (0.92-1.2)<br><br>Age stratified<br><45 y: 1.67 (1.16-2.4)<br>45-50 y: 1.02 (0.78-1.32)<br>> 50 y: 0.9 (0.68-1.19)

© 2026 Armeni E. *JAMA Network Open*. This is an open access article distributed under the terms of the CC-BY-NC-ND License, which does not permit alteration or commercial use, including those for text and data mining, AI training, and similar technologies.

Study (Year)	All-Cause Mortality -Absolute (%, n/N) / Relative Risk (RR, 95% CI)	All-Cause Mortality -Incident Risk (HR or MRR, 95% CI)
	> 50 y: 76/170 vs. 159/321	
Thong et al,[24] 2022	BSO vs. age-adjusted controls without iatrogenic menopause, Relative risk (IRR, 95% CI):<br>1.28 (1.15-1.41)	NR
Tuesley et al,[25] 2020	Hysterectomy + BSO vs. no surgery, Absolute risk (n/N):<br><br><u>Overall</u>:<br>1,101/18,558 vs. 25,575/553,958<br><br><u>Age-stratified</u><br>< 35 y: 59/1,014<br>35-44 y: 291/4,936<br>45-54 y: 414/8,599<br>55-64 y: 241/2,963<br>≥ 65 y: 96/1,046	Hysterectomy + BSO vs. no surgery, Relative risk (HR, 95% CI):<br><br><u>Overall:</u> HR 0.94 (0.88-1.00)<br><br><u>Age-stratified</u><br><35 y: 1.43 (1.11-1.85)<br>35-44 y: 1.19 (1.06-1.34)<br>45-54 y: 0.8 (0.73-0.88)<br>55-64 y: 0.9 (0.79-1.02)<br>≥ 65 y: 0.89 (0.73-1.09)
Wilson et al,[13] 2019	Absolute risk (n/N):<br><br>BSO+hysterectomy: 71/851<br>Hysterectomy: 167/2,472<br>No surgery: 663/10,218	Incident risk (HR, 95% CI):<br>BSO+hysterectomy vs. no surgery:<br>1.3 (1.02-1.66)<br>Hysterectomy vs. no surgery:<br>1.04 (0.88-1.24)
Xing et al,[2] 2024	BSO vs. natural menopause, Absolute risk (n/N):<br><br><u>Age-stratified</u><br>< 40 y: 306/870 vs. 216/525<br>≥ 40 y up to 50-54 y: 438/1437 vs. 2,130/6,238<br>≥ 55 y: 49/103 vs. 415/1174	BSO vs. Natural menopause age at ≥40 y, Incident risk (HR, 95% CI):<br><br><u>Age-stratified</u><br>< 40 y: 1.40 (1.20-1.65)<br>≥ 40 y: 0.94 (0.82-1.07)<br><br>BSO vs. BSO at age 45-54 y, Incident risk (HR, 95% CI):<br>< 40 y: 1.62 (1.31-2.02)<br>40-44 y: 1.15 (0.92-1.45)<br>45-54 y (reference)<br>≥ 55 y:1.00 (0.71-1.40)
Xu et al,[3] 2022	Absolute risk (n/N):<br><br><u>Age-stratified</u><br>< 40 y:<br>BSO: 67/2,534<br>Hysterectomy-only: 261/9,054<br>Natural menopause: 97/2,548<br>40-44 y:<br>BSO: 88/3,652<br>Hysterectomy-only: 193/7,953<br>Natural menopause: 305/10,045<br>45-49 y:<br>BSO: 168/6,064<br>Hysterectomy-only: 124/4,985<br>Natural menopause: 801/32,274<br>50-52 y:<br>BSO: 116/3,405<br>Hysterectomy-only: 26/1,269 vs.<br>Natural menopause: 1241/ 50,345<br>≥ 53 y:<br>BSO: 62/1914<br>Hysterectomy-only: 29/759	Incident risk (HR, 95% CI):<br><br><u>Age-stratified</u><br>< 40 y:<br>BSO: 1.31 (1.03-1.68)<br>Hysterectomy-only: 1.22 (1.07-1.39)<br>Natural menopause: 1.82 (1.48-2.24)<br>40-44 y:<br>BSO: 1.27 (1.02-1.57)<br>Hysterectomy-only: 0.91 (0.78-1.06)<br>Natural menopause: 1.26 (1.11-1.43)<br><br>45-49 y:<br>BSO: 1.22 (1.04-1.44)<br>Hysterectomy: HR 0.85 (0.71-1.02)<br>Natural menopause: 1.10 (1.01-1.20)<br>50-52 y:<br>BSO: 1.20 (0.99-1.45)<br>Hysterectomy-only: 0.71 (0.48-1.05)<br>Natural menopause: (reference)<br>≥ 53 y:<br>BSO: 1.07 (0.83-1.38)

© 2026 Armeni E. *JAMA Network Open*. This is an open access article distributed under the terms of the CC-BY-NC-ND License, which does not permit alteration or commercial use, including those for text and data mining, AI training, and similar technologies.

Study (Year)	All-Cause Mortality -Absolute (%, n/N) / Relative Risk (RR, 95% CI)	All-Cause Mortality -Incident Risk (HR or MRR, 95% CI)
	Natural menopause: 995/41,578	Hysterectomy-only: 1.30 (0.90-1.88)<br>Natural menopause: 0.89 (0.82-0.97)

Abbreviations: BSO, bilateral salpingo-oophorectomy; CI, confidence interval; FHPMI, family history of premature myocardial infarction; HR, hazard ratio; IRR, incidence rate ratio; MRR, mortality rate ratio; NR, not reported; RR, risk ratio; y, years.

© 2026 Armeni E. *JAMA Network Open*. This is an open access article distributed under the terms of the CC-BY-NC-ND License, which does not permit alteration or commercial use, including those for text and data mining, AI training, and similar technologies.

eTable 9. Fixed-effect sensitivity analysis of the pooled estimates shown in Figures 2 and 3

Outcome	Estimates	Iatrogenic menopause, No. of women	Control, No. of women	HR (95% CI), fixed effect	Heterogeneity: *Q* (df), *P*	I^2
**Cardiovascular morbidity**						
Composite cardiovascular events	7	53,004	1,260,756	1.23 (1.19-1.27)	25.13 (6), <.001	76%
Coronary heart disease	6	35,594	1,351,425	1.26 (1.21-1.32)	40.37 (5), <.001	88%
Stroke	4	34,094	1,203,561	1.22 (1.15-1.28)	4.02 (3), .26	25%
**Mortality**						
Cardiovascular mortality	7	41,166	634,909	1.04 (0.97-1.11)	11.03 (6), .09	46%
All-cause mortality	9	55,988	679,628	1.04 (1.01-1.07)	38.83 (8), <.001	79%

Hazard ratios were pooled using an inverse-variance-weighted fixed-effect model. Heterogeneity is expressed as Cochran *Q* with degrees of freedom and the corresponding *P* value, and as I^2 calculated as $(Q - \text{df})/Q$. Values differ from those shown on the random-effects figures, which calculate I^2 as $\tau^2/(\tau^2 + s^2)$ with τ^2 estimated by restricted maximum likelihood. The 2 expressions are algebraically identical when τ^2 is estimated by the method of DerSimonian and Laird, so the difference between the 2 sets of values reflects the estimator rather than the formula. Where restricted maximum likelihood returns $\tau^2 = 0$, as it does for stroke, the random-effects weights reduce to the fixed-effect weights and the 2 models return the same estimate.
Counts are numbers of women, not numbers of events, and are summed across the contributing estimates rather than across independent cohorts. The 7 cardiovascular mortality estimates arise from 6 independent cohorts, 2 of them being separate reports of the same Olmsted County population (Rivera et al,[7] 2009; Rocca et al,[6] 2006; eTable 17), so the deduplicated counts for that outcome are 40,075 women with iatrogenic menopause and 632,526 comparators.
For comparison, the corresponding random-effects estimates are 1.25 (95% CI, 1.12-1.39) for composite cardiovascular events, 1.48 (95% CI, 1.07-2.03) for coronary heart disease, 1.22 (95% CI, 1.15-1.28) for stroke, 1.03 (95% CI, 0.94-1.13) for cardiovascular mortality and 1.08 (95% CI, 1.01-1.16) for all-cause mortality.
Fixed-effect estimates lie closer to the null than the corresponding random-effects estimates for composite cardiovascular events, coronary heart disease and all-cause mortality, with narrower intervals, because the fixed-effect model gives greater weight to the largest cohorts. Stroke is identical under both models, and cardiovascular mortality is null under both, moving marginally away from the null. Given that heterogeneity was substantial for 3 of the 5 outcomes, this analysis is presented as a sensitivity comparison only; the random-effects estimates are primary in the main text and the Abstract.
Abbreviations: CI, confidence interval; df, degrees of freedom; HR, hazard ratio.

© 2026 Armeni E. *JAMA Network Open*. This is an open access article distributed under the terms of the CC-BY-NC-ND License, which does not permit alteration or commercial use, including those for text and data mining, AI training, and similar technologies.

eTable 10. GRADE certainty of evidence for each pooled outcome

Certainty assessment							№ of patients		Effect	Certainty	Importance
№ of estimates	Study design	Risk of bias	Inconsistency	Indirectness	Imprecision	Other considerations	Iatrogenic menopause	Comparators	Relative (95% CI)		
Composite cardiovascular events											
7	non-randomized studies	not serious	serious[a]	not serious	not serious	all plausible residual confounding would reduce the demonstrated effect	53,004	1,260,756	HR 1.25 (1.12 to 1.39)	⊕⊕◯◯ Low[a]	CRITICAL
Coronary heart disease											
6	non-randomized studies	not serious	very serious[b]	not serious	serious[b]	all plausible residual confounding would reduce the demonstrated effect	35,594	1,351,425	HR 1.48 (1.07 to 2.03)	⊕◯◯◯ Very low[b]	CRITICAL
Stroke											
4	non-randomized studies	not serious	not serious[c]	not serious	not serious	all plausible residual confounding would reduce the demonstrated effect	34,094	1,203,561	HR 1.22 (1.15 to 1.28)	⊕⊕⊕◯ Moderate[c]	CRITICAL
Cardiovascular mortality											

© 2026 Armeni E. *JAMA Network Open*. This is an open access article distributed under the terms of the CC-BY-NC-ND License, which does not permit alteration or commercial use, including those for text and data mining, AI training, and similar technologies.

Certainty assessment							№ of patients		Effect	Certainty	Importance
№ of estimates	Study design	Risk of bias	Inconsistency	Indirectness	Imprecision	Other considerations	Iatrogenic menopause	Comparators	Relative (95% CI)		
7[d]	non-randomized studies	not serious	not serious	not serious	serious[d]	none	41,166	634,909	HR 1.03 (0.94 to 1.13)	⊕◯◯◯ Very low[d]	CRITICAL
All-cause mortality											
9	non-randomized studies	not serious	serious[e]	not serious	serious[e]	none	55,988	679,628	HR 1.08 (1.01 to 1.16)	⊕◯◯◯ Very low[e]	CRITICAL

CI: confidence interval; HR: hazard ratio

Explanations

a. Substantial heterogeneity (I^2 = 86%), concentrated in a single cohort whose omission reduces it to 0% (eTable 16): rated down 1 level for inconsistency and rated up 1 level because all plausible residual confounding would reduce the demonstrated effect.
b. Very substantial heterogeneity (I^2 = 97%) with contributing estimates ranging from 1.12 to 3.76, and a pooled interval spanning 1.07 to 2.03 that includes the null on omission of a single cohort (eTable 16): rated down 2 levels for inconsistency and 1 level for imprecision, and rated up 1 level because all plausible residual confounding would reduce the demonstrated effect.
c. No heterogeneity detected under the random-effects model: the restricted maximum-likelihood estimate of the between-study variance lies at 0, so I^2 = 0% and the fixed-effect and random-effects estimates coincide, while the classical (Q - df)/Q value calculated from the same Q of 4.02 (df = 3; P = .26) is 25% (eTable 9). Rated up 1 level because all plausible residual confounding would reduce the demonstrated effect.
d. The pooled estimate includes the null and is imprecise (I^2 = 40%): rated down 1 level for imprecision. No rating up was applied, because no effect was demonstrated. The 7 estimates arise from 6 independent cohorts, 2 of them separate reports of the same Olmsted County population (eTable 17).
e. Substantial heterogeneity (I^2 = 79%) and a pooled interval whose lower limit lies at 1.01, conventional significance being lost on omission of 1 cohort and the estimate sitting at the threshold on omission of a second (eTable 16): rated down 1 level each for inconsistency and imprecision. No rating up was applied.
Counts are numbers of women, not numbers of events. Effect estimates are the random-effects pooled hazard ratios, which are primary for every outcome (eTable 9 reports the corresponding fixed-effect estimates). All contributing studies are non-randomized, so each outcome begins at low certainty.

© 2026 Armeni E. *JAMA Network Open*. This is an open access article distributed under the terms of the CC-BY-NC-ND License, which does not permit alteration or commercial use, including those for text and data mining, AI training, and similar technologies.

eTable 11. Descriptive characteristics of the included studies

Study (year)	Type of study	Geographic location, Healthcare setting	Race and ethnicity	Sample size (to include N of cases and comparators)	Cause of iatrogenic menopause (if available)	Control definition
Abbas et al,[26] 2018	Cross-sectional study	India, Menopause Clinic of Medical College Hospitals	NR	127<br>Cases: 31<br>Comparators: 64	Hysterectomy + BSO	Natural menopause
Abildgaard et al,[16] 2018	Retrospective cohort study	Denmark, Danish national health registries	NR	20,020<br>Cases: 2,002<br>≤45 y non-MHT use: 302<br>≤45 y MHT users: 564<br>> 45 y: 1136<br><br>Comparators: 18,018<br>≤45 y non-MHT use: 2,718<br>≤45 y MHT use: 5,076<br>> 45 y: 10,224	Prophylactic oophorectomy with a simultaneous diagnosis of a family history of cancer	Population age matched comparison
Allison et al,[17] 2008	Randomized controlled trial	USA, multicenter study	American Indian, Asian/Pacific Islander, Black, Hispanic, White	1,064<br>Cases: 378<br>Comparators: 459	BSO	Hysterectomy / No oophorectomy
Appiah et al,[5] 2015	Prospective observational study	USA, multicenter study	White	7,977<br>Cases (BSO, no diabetes): 1,340<br>Cases (BSO, diabetes): 101<br>Comparators (diabetes, no BSO): 401<br>Comparators (no BSO, no diabetes): 6,135	BSO	No surgery
Appiah et al,[4] 2020	Retrospective cohort study	USA, National Center for Health Statistics	Non-Hispanic Black, Non-Hispanic White, other	2,763<br>Cases (BSO, no FHPMI): 515<br>Cases (BSO and FHPMI): 95<br>Comparators (FHPMI, no BSO): 243<br>Comparators (no BSO, no FHPMI):1,915	BSO	No surgery
Chen et al,[36] 2023	Randomized controlled trial	USA, National Institute on Aging, National Institutes of Health	Asian or Pacific Islander, Black non-Hispanic, Hispanic, White non-Hispanic	590<br>Cases: 79<br>Comparators (natural menopause): 476<br>Comparators (hysterectomy-only): 35	Hysterectomy + BSO	Natural menopause or Hysterectomy-only

© 2026 Armeni E. *JAMA Network Open*. This is an open access article distributed under the terms of the CC-BY-NC-ND License, which does not permit alteration or commercial use, including those for text and data mining, AI training, and similar technologies.

Study (year)	Type of study	Geographic location, Healthcare setting	Race and ethnicity	Sample size (to include N of cases and comparators)	Cause of iatrogenic menopause (if available)	Control definition
Dam et al,[18] 2019	Case-cohort study	EPIC-CVD; Denmark, France, Germany, Greece, Italy, Norway, Spain, Sweden, the Netherlands, and the UK	NR	9916<br>Cases: 2206<br>Comparators: 7710	Bilateral oophorectomy	Natural menopause
Dorum et al,[27] 2008	Cross-sectional study	Norway, HUNT-2 study, a rural and urban Norwegian county	White	1,052<br>Cases 263<br>Comparators: 789	BSO ± Hysterectomy	No oophorectomy, age matched
Do Valle et al,[19] 2021	Retrospective population-based cohort	Canada (British Columbia provincial registry)	NR	7560<br>BRCA+ RRBSO: 360<br>BSO (benign, no mutation): 3,600<br>Intact ovaries: 3,600	RRBSO for BRCA1/2 mutation carriers	Age-matched women with benign BSO (no mutation) or intact ovaries (no mutation)
Duan et al,[20] 2012	Retrospective cohort study	USA, California Teachers Study (CTS)	African American, White, or other (Asian/Pacific Islander, Hispanic, mixed, Native American, not reported)	42,004<br>Cases: 9,785<br>Comparators: 32,219	BSO	Natural menopause
Farahmand et al,[21] 2014	Cohort, Case-control study	Iran, Tehran Lipid and Glucose Study	NR	218<br>Cases: 40<br>Comparators: 178	No precise definition	Natural menopause
Farland et al,[11] 2023	Prospective cohort study	USA, multicenter	Non-Hispanic White	110,601<br>Cases: 2,153<br>Comparators (no surgery): 108,448<br>Comparators (hysterectomy-only): 2,960	Hysterectomy+BSO	No surgery or Hysterectomy-only
Gierach et al,[10] 2014	Prospective cohort study	USA, Breast Cancer Detection	Non-Hispanic White vs other/unknown:	41,599<br>Cases: 12,652<br>Comparators (no surgery): 28,947	BSO	No surgery or Hysterectomy-only

© 2026 Armeni E. *JAMA Network Open*. This is an open access article distributed under the terms of the CC-BY-NC-ND License, which does not permit alteration or commercial use, including those for text and data mining, AI training, and similar technologies.

Study (year)	Type of study	Geographic location, Healthcare setting	Race and ethnicity	Sample size (to include N of cases and comparators)	Cause of iatrogenic menopause (if available)	Control definition
		Demonstration Project (BCDDP)	-BSO: 86.9% vs. 13.1%<br>-Hysterectomy-only: 86.1% vs. 13.9%<br>-None: 86.3% vs 13.7%	Comparators (hysterectomy-only): 11,247		
Honigberg et al,[12] 2019	Prospective cohort study	USA, UK Biobank	Asian, Black, Mixed, White, Other	144,260<br>Cases (surgical premature menopause): 644<br>Comparators (menopause at least 40 y): 138,712	BSO	Natural menopause
Howard et al,[22] 2005	Prospective cohort (Women's Health Initiative Observational Study)	USA, 40 clinical centers (community-based)	Black, 9%; Hispanic, 4%; White, 83%; Other, 4%	89,915<br>-Hysterectomy+BSO: 18,251<br>-Hysterectomy-only: 18,687<br>-No surgery: 52,976	Hysterectomy + BSO	No surgery or Hysterectomy-only
Indhavivadhana et al,[28] 2011	Cross-sectional study	Thailand, university hospital	NR	640<br>Cases: 198<br>Comparators: 442	BSO ± Hysterectomy	Natural menopause
Ingelsson et al,[1] 2010	Prospective cohort study	Sweden, Inpatient Register data	NR	833,484<br>Hysterectomy+BSO: 28,136<br>Only hysterectomy: 156,305<br>Only BSO: 5,118<br>No surgery: 643,925	BSO ± Hysterectomy	Age matched, No surgery or hysterectomy-only

© 2026 Armeni E. *JAMA Network Open*. This is an open access article distributed under the terms of the CC-BY-NC-ND License, which does not permit alteration or commercial use, including those for text and data mining, AI training, and similar technologies.

Study (year)	Type of study	Geographic location, Healthcare setting	Race and ethnicity	Sample size (to include N of cases and comparators)	Cause of iatrogenic menopause (if available)	Control definition
Jamali et al,[29] 2024	Cross-sectional study	Iran, Rafsanjan Cohort Study (RCS), branch of the Prospective Epidemiological Research Studies in Iran (PERSIAN)	NR	2,076<br>Cases: 457<br>Comparators: 1,619	BSO ± Hysterectomy or Chemotherapy-induced	Natural menopause
Krul et al,[23] 2018	Retrospective cohort	Netherlands	NR	918 Hodgkin lymphoma survivors; 299 with POI vs survivors without POI	Cancer treatment-induced POI after alkylating chemotherapy and/or pelvic radiotherapy	Lymphoma survivors with menopause ≥40 years or still pre-menopausal
Mack et al,[30] 2004	Cross-sectional study	USA, single center study	NR	189<br>Cases: 57<br>Comparators: 132	Hysterectomy + BSO	Natural menopause
Michelsen et al,[8] 2023	Prospective cohort study	Norway, Part of the Trøndelag Health Study, Norwegian Cause of Death Registry (HUNT2)	NR	19,580<br>Cases: 907<br>Comparators (no oophorectomy): 18,673<br>Comparators (hysterectomy-only): 1,199	BSO ± Hysterectomy	No oophorectomy or Hysterectomy-only
Netterlid et al,[31] 2021	Cross-sectional case–control	Swedish Cancer Registry / Skåne University Hospital	NR	167 childhood cancer survivors vs 164 matched controls	Ovarian failure after childhood cancer therapy (radiotherapy, chemotherapy, HSCT)	Population controls matched for age, sex, ethnicity, residence, smoking
Olesen et al,[14] 2022	Prospective cohort study	Denmark, University Hospital	NR	25,338<br>Cases: 2,040<br>Comparators: 22,358	BSO ± hysterectomy	Age-adjusted controls without iatrogenic menopause

© 2026 Armeni E. *JAMA Network Open*. This is an open access article distributed under the terms of the CC-BY-NC-ND License, which does not permit alteration or commercial use, including those for text and data mining, AI training, and similar technologies.

Study (year)	Type of study	Geographic location, Healthcare setting	Race and ethnicity	Sample size (to include N of cases and comparators)	Cause of iatrogenic menopause (if available)	Control definition
Ou et al,[32] 2023	Cross-sectional study	Taiwan, multicenter	NR	11,408<br>Cases: 1,967<br>Comparators: 9,441	BSO ± hysterectomy	Natural menopause, matched for age where possible
Ozdemir et al,[33] 2009	Cross-sectional study	Turkey, single center	NR	189<br>Cases: 94<br>Controls: 95	Hysterectomy + BSO	Natural menopause, matched for age where possible
Ozkaya et al,[34] 2011	Cross-sectional study	Turkey, single center	NR	90<br>Cases: 45<br>Comparators: 45	Hysterectomy + BSO	Natural menopause, matched for age where possible
Poorthuis et al,[9] 2022	Retrospective cohort study	China Kadoorie Biobank	Chinese	282,722<br>Cases: 1,360<br>Comparators (no surgery): 272,884<br>Comparators (hysterectomy-only): 8,478	Hysterectomy + BSO	No surgery or Hysterectomy-only
Rivera et al,[7] 2009	Retrospective cohort study	USA	Predominantly White, of European ancestry	3,474<br>Cases: 1,091<br>Comparators: 2,383	BSO	Women without oophorectomy, individually matched by year of birth and drawn from the same population; hysterectomy was not an exclusion
Rocca et al,[6] 2006	Retrospective cohort study	USA	Predominantly White, of European ancestry	3,474<br>Cases: 1,091<br>Comparators: 2,383	BSO	Women without oophorectomy, individually matched by year of birth and drawn from the same population; hysterectomy was not an exclusion
Thong et al,[24] 2022	Retrospective cohort study	Australia	NR	17,839<br>Cases: 414<br>Comparators: 16,998	BSO	Age-adjusted controls without iatrogenic menopause
Tuesley et al,[25] 2020	Retrospective cohort study	Australia	Predominantly European ancestry	665,792<br>Cases: 18,558<br>Comparators (no surgery): 553,958	Hysterectomy + BSO	No surgery or Hysterectomy-only

© 2026 Armeni E. *JAMA Network Open*. This is an open access article distributed under the terms of the CC-BY-NC-ND License, which does not permit alteration or commercial use, including those for text and data mining, AI training, and similar technologies.

Study (year)	Type of study	Geographic location, Healthcare setting	Race and ethnicity	Sample size (to include N of cases and comparators)	Cause of iatrogenic menopause (if available)	Control definition
				Comparators (hysterectomy-only): 73,145		
Tuysuzoglu et al,[35] 2020	Cross-sectional study	Turkey, single center	NR	70<br>Cases: 35<br>Controls: 35	BSO	Natural menopause, matched for age where possible
Wilson et al,[13] 2019	Prospective cohort study	Australia	White Australian women	13,541<br>Cases: 851<br>Comparators (no surgery): 10,218<br>Comparators (hysterectomy-only): 2,472	Hysterectomy + BSO	No surgery or Hysterectomy-only
Xing et al,[2] 2024	Retrospective cohort study	USA	Non-Hispanic White; other, including Asian, Hispanic, multiracial, and non-Hispanic Black women	14,161<br>Cases: 2,410<br>Comparators: 7,937	BSO	Natural menopause, matched for age where possible
Xu et al,[3] 2022	Prospective cohort study	Australia, UK Biobank	Asian, Black, White, others	178,379<br>Cases: 17,569<br>Comparators (natural menopause): 136,790<br>Comparators (hysterectomy-only): 24,020	BSO	Natural menopause or Hysterectomy-only
Zhu et al,[15] 2020	Observational cohort study	Australia	Asian, Black, White-American, White-Australian, White-European, Others	203,767<br>-Cases: 25,463<br>-Comparators: 178,304	BSO ± hysterectomy	Natural menopause matched for age where possible

Race and ethnicity were reported as classified by the primary studies; categories are listed as reported, in alphabetical order, with groups labelled "other" last. Groups labelled "other" comprise, where specified by the primary study: Asian/Pacific Islander, Hispanic, mixed or multiracial, Native American, and not reported. NR indicates that race and ethnicity were not reported by the primary study; the geographic location of each study is given in a separate column.

Abbreviations: BCDDP, Breast Cancer Detection Demonstration Project; BRCA, breast cancer susceptibility gene; BSO, bilateral salpingo-oophorectomy; CTS, California Teachers Study; CVD, cardiovascular disease; EPIC, European Prospective Investigation into Cancer and Nutrition; FHPMI, family history of premature myocardial infarction; HSCT, hematopoietic stem cell transplantation; HUNT-2, Nord-Trøndelag Health Study (second survey); MHT, menopausal hormone therapy; NR, not reported; PERSIAN, Prospective Epidemiological Research Studies in Iran; POI, premature ovarian insufficiency; RCS, Rafsanjan Cohort Study; RRBSO, risk-reducing bilateral salpingo-oophorectomy; UK, United Kingdom; USA, United States of America; y, years.

© 2026 Armeni E. *JAMA Network Open*. This is an open access article distributed under the terms of the CC-BY-NC-ND License, which does not permit alteration or commercial use, including those for text and data mining, AI training, and similar technologies.

eTable 12. Main anthropometric characteristics of participants and menopause-related characteristics in the included studies

Study (year)	Age of cases and comparators (y) (mean ± SD) or (median (IQR) or (%, N)	Body mass index of cases and comparators (kg/m²) (mean ± SD) or (%, N)	MHT use (%, N)	Time of follow up (y) (mean) or (median (IQR))
Abbas et al,[26] 2018	Cases: 54.2 ± 4.21<br>Comparators: 58.2 ± 3.14	Cases: 24.6 ± 4.3<br>Comparators: 24.3 ± 4.6	NR	NR
Abildgaard et al,[16] 2018	Cases:<br>≤45 y:<br>-non-MHT use: 42 (39–44)<br>-MHT users: 40 (37–42)<br>>45 y: 51 (48–57)<br><br>Comparators: age matched at each group	NR	NR	Total 8.7 y<br>Cases:<br>≤45 y:<br>-non-MHT use: 6.2 (3.9–8.9)<br>-MHT users: 5.1 (2.8–8.2)<br>> 45 y: 5.3 (3.1–8.2)<br><br>Comparators (respective to each case group):<br>-6.2 (3.9-9.0)<br>-5.1 (2.8-8.2)<br>-5.3 (3.0-8.2)
Allison et al,[17] 2008	NR	NR	Cases: 72%, 272 vs. Comparators: 42%, 192	NR
Appiah et al,[5] 2015	Cases (BSO, no diabetes): 71 ± 4.8<br>Cases (BSO, diabetes): 71.3 ± 4.8 Comparators (diabetes, no BSO): 71.7 ± 5.1<br>Comparators (no BSO, no diabetes): 71.7 ± 5.4	Cases (BSO, no diabetes) and Cases (BSO, diabetes) vs. Comparators (diabetes, no BSO) and Comparators (no BSO, no diabetes):<br>BMI:<br><25 kg/m^2: 45.8% and 35.2% vs. 26.7% and 44.7%<br>25.0-29.9 kg/m^2: 35.6% and 30.5% vs. 36.9% and 37.2%<br>≥30 kg/m^2:<br>18.6% and 34.3% vs. 36.4% and 18.1%	Cases (BSO, no diabetes) and Cases (BSO, diabetes) vs. Comparators (diabetes, no BSO) and Comparators (no BSO, no diabetes):<br><br>Never:<br>31.5% and 41.9% vs. 73.8% and 57.5%<br>Former:<br>43.3% and 42.9% vs. 22.2% and 32.5%<br>Current:<br>25.2% and 15.2% vs. 4.0% and 10.0%	15.1
Appiah et al,[4] 2020	Cases (BSO, no FHPMI) (mean (SE)): 60.0 (0.5)<br>Cases (BSO and FHPMI): 58.2 (0.8)<br>Comparators (FHPMI, no BSO): 58.5 (0.7)<br>Comparators (no BSO, no FHPMI): 63.7 (0.3)	Cases (BSO, no FHPMI) (mean (SE)): 27.7 (0.2)<br>Cases (BSO and FHPMI): 28.0 (0.5)<br>Comparators (FHPMI, no BSO): 28.4 (0.4)<br>Comparators (no BSO, no FHPMI): 27.6 (0.1)	Cases (BSO, no FHPMI) (mean (SE)): 48.3%, 198<br>Cases (BSO and FHPMI): 47.5%, 36<br>Comparators (FHPMI, no BSO): 18.5%, 32<br>Comparators (no BSO, no FHPMI): 13.7%, 189	22
Chen et al,[36] 2023	Cases: 60.8 ± 8.4<br>Natural menopause: 59.3 ± 8.9<br>Hysterectomy-only: 67.3 ± 8.9	Cases: 27.3 ± 8.0<br>Natural menopause: 26.1 ± 7.0	Cases: 93.7%, 74<br>Natural menopause: 66.8%, 318	4.8

© 2026 Armeni E. *JAMA Network Open*. This is an open access article distributed under the terms of the CC-BY-NC-ND License, which does not permit alteration or commercial use, including those for text and data mining, AI training, and similar technologies.

Study (year)	Age of cases and comparators (y) (mean ± SD) or (median (IQR) or (%, N)	Body mass index of cases and comparators (kg/m²) (mean ± SD) or (%, N)	MHT use (%, N)	Time of follow up (y) (mean) or (median (IQR))
		Hysterectomy-only: 27.9 ± 7.1	Hysterectomy-only: 97.1%, 34	
Dam et al,[18] 2019	Cases: 57.4 ± 8.0<br>Comparators: 60.4 ± 6.2	Cases vs. comparators:<br>BMI:<br>≤20 kg/m^2: 3.3%, 72 vs. 4.5%, 346<br>>20 -<25 kg/m^2: 36.0%, 789 vs. 37.3%, 2864<br>≥25-<30 kg/m^2: 39.5%, 865 vs. 37.4% 2874<br>≥30 kg/m^2: 21.3%, 466 vs. 20.7%, 1591	Cases vs. comparators:<br>Ever MHT use: 40.8%, 901 vs. 31.1%, 2105	11
Dorum et al,[27] 2008	Cases: 56.4 ± 7.7<br>Comparators: 56.4 ± 7.8	Cases vs. comparators:<br>BMI ≥ 30kg/m^2: 30%, 80 vs. 21%, 169	Cases: 38%, 101<br>Comparators: 13%, 101	15
Do Valle et al,[19] 2021	BRCA+RRBSO: 42.5 ± 4.9<br>BSO (benign, no mutation): 42.6 ± 4.9<br>Intact ovaries (no mutation): 42.6 ± 4.9	NR	BRCA+RRBSO: 44.7%, 161<br>BSO, no mutation: 64.5%, 2323<br>Intact ovaries (no mutation): NR	BRCA+RRBSO: 7.34 ± 4.40<br>BSO, non-BRCA: 9.82 ± 5.57<br>Intact ovaries, median 8.9±5.71
Duan et al,[20] 2012	Cases:<br>Never MHT use: 67.9 ± 10.4<br>Ever MHT use: 59.2 ± 9.8<br><br>Comparators:<br>Never MHT use: 65.4 ± 9.3<br>Ever MHT use: 62.7 ± 9.0	Cases vs. comparators:<br>Never MHT use:<br>BMI<br>16-24.9 kg/m^2: 41.8 % vs. 8.1%<br>25-29.9 kg/m^2: 29.5 % vs. 26.9%<br>30-54.9 kg/m^2: 22.0% vs. 17.1%<br><br>Ever MHT use:<br>BMI<br>16-24.9 kg/m^2: 50.9% vs. 57.4%<br>25-29.9 kg/m^2: 27.6% vs. 26.5%<br>30-54.9 kg/m^2: 17.0% vs. 11.6%	Cases: 97%, 9,476<br>Comparators: 66%, 21,368	11.3
Farahmand et al,[21] 2014	Cases: 42.6 ± 4.6<br>Comparators: 49.4 ± 4.0	Cases: 28.8 ± 4.1<br>Comparators: 29.7 ± 4.6	NR	NR
Farland et al,[11] 2023	Cases: 38.8 ± 3.3<br>Comparators (no surgery): 34.6 ± 4.6<br>-Comparators (hysterectomy-only): 38.9 ± 3.1	Cases: 24.8 ± 5.4<br>Comparators (no surgery): 24.0 ± 5.0<br>Comparators (hysterectomy-only): 25.0 (5.4)	NR	30
Gierach et al,[10] 2014	Cases vs. Comparators (no surgery) vs. Comparators (hysterectomy-only):	Cases vs. Comparators (no surgery) vs.	Cases vs. Comparators (no surgery) vs.	22.1

© 2026 Armeni E. *JAMA Network Open*. This is an open access article distributed under the terms of the CC-BY-NC-ND License, which does not permit alteration or commercial use, including those for text and data mining, AI training, and similar technologies.

Study (year)	Age of cases and comparators (y) (mean ± SD) or (median (IQR) or (%, N)	Body mass index of cases and comparators (kg/m²) (mean ± SD) or (%, N)	MHT use (%, N)	Time of follow up (y) (mean) or (median (IQR))
	<50 y: 32.4% vs. 31.4% vs. 32.4%<br>50-54 y: 22.2% vs. 22.3% vs. 22.2%<br>55-59 y: 19.3% vs. 18.9% vs. 19.3%<br>60-64 y: 12.0% vs. 12.1% vs. 12.0%<br>65-69 y: 7.6% vs. 7.5% vs. 7.6%<br>70-74 y: 4.5% vs. 5.2% vs. 4.5%<br>75-79 y: 1.7% vs. 2.2% vs. 1.7%<br>≥80 y: 0.2% vs. 0.4% vs. 0.2%	Comparators (hysterectomy-only): BMI<br><25 kg/m²: 63.8% vs. 66.8% vs. 63.1%<br>25–<30 kg/m²: 25.3% vs. 24.0% vs. 26.6%<br>≥30 kg/m²: 10.9% vs. 9.2% vs. 10.3%	Comparators (hysterectomy-only):<br><br>Never MHT users: 15.0% vs. 47.1% vs. 23.8%<br>ET <10 y: 27.9% vs. 12.1% vs. 33.8%<br>ET ≥10 y: 35.6% vs. 2.0% vs. 24.6%<br>Other/unknown: 21.4% vs. 38.8% vs. 17.8%	
Honigberg et al,[12] 2019	Cases (surgical premature menopause): 57.4 ± 7.8<br>Comparators (natural premature menopause): 58.9 ± 7.1<br>Menopause at least at 40 y of age: 60.0 ± 5.4	Cases (surgical premature menopause) :28.7 ± 6.0<br>Comparators (natural premature menopause): 27.9 ± 5.3<br>Menopause at least at 40 y of age: 27.0 ± 4.9	Cases: 84.5%, 544<br>Comparators: 71.5%, 3,490<br>Menopause at least at 40 y of age: 44.1%	NR
Howard et al,[22] 2005	Hysterectomy + BSO (mean): 63.97<br>Hysterectomy only (mean): 63.62<br>No surgery (mean): 63.35	Hysterectomy + BSO (mean): 28.4<br>Hysterectomy only (mean): 28.1<br>No surgery (mean): 26.9	Hysterectomy + BSO, 56%<br>Hysterectomy only, 51%<br>No surgery, 28%	5.1
Indhavivadhana et al,[28] 2011	Cases: 49.2 ± 6.7<br>Comparators: 53.0 ± 5.0	Cases: 23.9 ± 3.3<br>Comparators: 23.6 ± 3.5	Cases: 31%, 61<br>Comparators: 3.9%, 17	NR
Ingelsson et al,[1] 2010	NR	NR	NR	10.4 (5-17.1)
Jamali et al,[29] 2024	NR	NR	NR	NR
Krul et al,[23] 2018	Median age at end of follow-up 50 y	NR	19.3% (177/918) used HRT	Median 24 years
Mack et al,[30] 2004	Cases: 61.6 ± 7.9<br>Comparators: 60.8 ± 6.7	Cases: 29.6 ± 5.9<br>Comparators: 28.8 ± 5.7	None	NR
Michelsen et al,[8] 2023	Cases: 64.5 ± 11.9<br>Comparators (no oophorectomy): 43.0 ± 13.7<br>Comparators (hysterectomy-only): 64.5 ± 11.9	Cases: 27.7 ± 4.5<br>Comparators (no oophorectomy): 25.8 ± 4.5<br>Comparators (hysterectomy-only): 27.4 ± 4.5	Cases: 24.3%, 220)<br>Comparators (no oophorectomy): 7.5%, 1,414<br>Comparators (hysterectomy-only): 21.2%, 254)	Cases: 16.9<br>Comparators (no oophorectomy): 17.3<br>Comparators (hysterectomy-only): 17.1
Netterlid et al,[31] 2021	CCS 34 y (19–57); controls 35 y (19–58)	CCS median 24 kg/m² (16–44); controls 23 kg/m² (18–35)	CCS 12% (20/167) HRT; 68% (15/22) among POI	Median 25 years since cancer diagnosis
Olesen et al,[14] 2022	Cases: 50 (45-68)<br>Comparators: 50 (44-71)	Cases vs. comparators:<br>BMI:	Cases: 45.4%, 927<br>Comparators 25.6%, 5,730	NR

© 2026 Armeni E. *JAMA Network Open*. This is an open access article distributed under the terms of the CC-BY-NC-ND License, which does not permit alteration or commercial use, including those for text and data mining, AI training, and similar technologies.

Study (year)	Age of cases and comparators (y) (mean ± SD) or (median (IQR) or (%, N)	Body mass index of cases and comparators (kg/m²) (mean ± SD) or (%, N)	MHT use (%, N)	Time of follow up (y) (mean) or (median (IQR))
		<18.5 kg/m²: 1.5% vs. 2.6%<br>18.5-24.9 kg/m²: 67.8% vs. 69.2%<br>25-29.9 kg/m²: 23% vs. 22.7%<br>>30 kg/m²: 7.7% vs. 5.5%		
Ou et al,[32] 2023	NR	NR	NR	NR
Ozdemir et al,[33] 2009	Cases: 52.69 ± 5.02<br>Comparators: 53.01 ± 4.83	Cases: 29.69 ± 3.79<br>Comparators: 30.01 ± 4.84	None	NA
Ozkaya et al,[34] 2011	Cases: 62 ± 10.8<br>Comparators: 60 ± 7.7	Cases: 31 ± 3.1<br>Comparators: 30.4 ± 2.7	None	NR
Poorthuis et al,[9] 2022	Cases: 53.2 ± 7.8<br>Comparators (no surgery): 50.9 ± 10.4<br>Comparators (hysterectomy-only): 51.9 ± 7.5	Cases: 23.1 ± 3.4<br>Comparators (no surgery): 23.7 ± 3.4<br>Comparators (hysterectomy-only): 24.3 ± 3.4	NR	9.8
Rivera et al,[7] 2009	NR	NR	NR	Cases: 25.0 (min:4 days–max:53.8 y)<br>Comparators: 26.4 (min: 66 days–max:55.1 y)
Rocca et al,[6] 2006	NR	NR	NR	Cases: 25.0 (0.01–53.8)<br>Comparators: 26.4 (0.2–55.1)
Thong et al,[24] 2022	Cases: 39.0 ± 4.3	NR	NR	36
Tuesley et al,[25] 2020	Cases: 39 (min:15-max:78) vs.<br>Comparators (no surgery): 33 (min:15-max:80)<br>Comparators (hysterectomy-only): 39 (min:15-max:79)	NR	NR	24.2 (max: 26.7)
Tuysuzoglu et al,[35] 2020	Cases: 50.05 ± 2.74<br>Comparators: 50.62 ± 3.78	Cases: 31.24 ± 4.87<br>Comparators: 30.00 ± 5.2	None	NR
Wilson et al,[13] 2019	Cases vs. Comparators (hysterectomy-only) vs. Comparators (no surgery):<br><br>Age 45:<br>4.3%, 129 vs. 16.3%, 493 vs. 79.4%, 2,042<br>Age 46:<br>5.4%, 168 vs. 16.9%, 485 vs. 77.7%, 2,142<br>Age 47:<br>5.9%, 169 vs. 16.5%, 445 vs. 77.6%, 1,981<br>Age 48:	Cases vs. Comparators (hysterectomy-only) vs. Comparators (no surgery):<br><br>BMI:<br><18.5 kg/m²: 6.2%, 16 vs. 15.1%, 34 vs. 78.7%, 179<br>18.5–24.9 kg/m²: 4.6%, 313 vs. 15.3%, 1,073 vs. 80.1%, 5,225	Cases vs. Comparators (hysterectomy-only) vs. Comparators (no surgery):<br><br>82.5%, 702/851 vs. 40.0%, 990/2,472 vs. 19.3%, 1,972/10,218	21.5

© 2026 Armeni E. *JAMA Network Open*. This is an open access article distributed under the terms of the CC-BY-NC-ND License, which does not permit alteration or commercial use, including those for text and data mining, AI training, and similar technologies.

Study (year)	Age of cases and comparators (y) (mean ± SD) or (median (IQR) or (%, N)	Body mass index of cases and comparators (kg/m²) (mean ± SD) or (%, N)	MHT use (%, N)	Time of follow up (y) (mean) or (median (IQR))
	6.2%, 164 vs. 18.2%, 476 vs. 75.6%, 1,932<br>Age 49:<br>7.1%, 208 vs. 19.1%, 534 vs. 73.7%, 2,018<br>Age 50:<br>14.6%, 13 vs. 25.0%, 39 vs. 60.4%, 103	25–29.9 kg/m^2: 6.6%, 262 vs. 18.8%, 712 vs. 74.6%, 2,784<br>≥30 kg/m^2: 8.5%, 224 vs. 23.0%, 561 vs. 68.5%, 1,636		
Xing et al,[2] 2024	Cases vs. Comparators (natural menopause):<br>Age at menopause subgroups:<br><40 y: 62.2 ± 12.0 vs. 62.7 ± 13.2<br>40-44 y: 63.8 ± 11.5 vs. 64.4 ± 11.8<br>45-54 y: 65.6 ± 9.7 vs. 64.6 ± 10.1<br>>55 y: 72.0 ± 7.4 vs. 68.8 ± 8.2	Cases vs. Comparators (natural menopause):<br>Age at menopause subgroups:<br><40 y: 30.0 ± 6.9 vs. 29.4 ± 7.0<br>40–44 y: 29.7 ± 6.9 vs. 29.2 ± 6.9<br>45–54 y: 29.7 ± 6.7 vs. 29.1 ± 7.0<br>≥55 y: 30.4 ± 7.7 vs. 29.4 ± 7.0	Cases vs. Comparators (natural menopause): Age at menopause subgroups:<br><40 y: 79.4%, 617 vs. 22.8%, 97<br>40-44 y: 76.6%, 385 vs. 31.5%, 208<br>45-54 y: 72.1%, 602 vs. 30.3%, 1260<br>>55 y: 70.2%, 64 vs. 39.3%, 360	Maximum, 31 y
Xu et al,[3] 2022	NR	Cases vs. Comparators (natural menopause) vs. Comparators (hysterectomy-only):<br>BMI:<br><18.5 kg/m^2: 0.4% vs. 0.8% vs. 0.5%<br>18.5–24.9 kg/m^2: 30.8% vs. 39.0% vs. 30.1%<br>25–29.9 kg/m^2: 38.8% vs. 37.9% vs. 40%<br>≥30 kg/m^2: 30% vs. 22.3% vs. 29.5%	NR	10.3
Zhu et al,[15] 2020	Cases: 57.5±7.5<br>Comparators: 58.1±7.1	Cases vs comparators:<br>BMI<br><18.5 kg/m^2: 0.7%, 173 vs. 1.1%, 1,986<br>18.5-24.9 kg/m^2: 35.6%, 9,060 vs. 43.7%, 77,971<br>25-29.9 kg/m^2: 37%, 9,433 vs. 35.5%, 63,358<br>≥30 kg/m^2: 26.7%, 6,797 vs. 19.7%, 35,069	Cases: 25.8%, 6,571 vs.<br>Comparators: 59.5%, 106,094	NR

Abbreviations: BMI, body mass index; BRCA, breast cancer susceptibility gene; BSO, bilateral salpingo-oophorectomy; CCS, childhood cancer survivors; ET, estrogen therapy; FHPMI, family history of premature myocardial infarction; IQR, interquartile range; MHT, menopausal hormone therapy; NR, not reported; POI, premature ovarian insufficiency; RRBSO, risk-reducing bilateral salpingo-oophorectomy; SD, standard deviation; SE, standard error; y, years.

© 2026 Armeni E. *JAMA Network Open*. This is an open access article distributed under the terms of the CC-BY-NC-ND License, which does not permit alteration or commercial use, including those for text and data mining, AI training, and similar technologies.

eTable 13. Studies reporting on the role of menopausal hormone therapy on the absolute, relative, and incident risk for cardiovascular disease, coronary heart disease, and stroke morbidity, and for cardiovascular and all-cause mortality.

Study (year)	CVD or CHD or Stroke morbidity/mortality– Absolute / Incident or Relative Risk	All-Cause Mortality – Absolute (prevalence %, n/N) /. Incident or Relative Risk (MRR, 95% CI)
Abildgaard et al,[16] 2018	NR	Oophorectomy age at ≤ 45 y vs. age-matched population control<br><br>MHT-users vs. non-MHT users:<br>Absolute risk (%, n/N): 1.4%, 8/564 vs. 0.3%, 1/302<br>Relative risk (MRR, 95% CI): 3.45 (1.53-7.79) vs. 0.56 (0.07-4.22)
Do Valle et al,[19] 2021	MHT users vs. non-MHT users, Incident risk (HR, 95% CI):<br>CVD events:<br>BRCA-RRSO: 0.93 (0.41-2.09)	NR
Olesen et al,[14] 2022	BSO/ever MHT users vs. Natural menopause/ever MHT users, Relative risk (RR, 95% CI):<br><br>Overall:<br>0.77 (0.67-0.88) vs. 0.85 (0.77-0.94)<br><br>Age at menopause<br>≥45 y: 0.75 (0.64-0.88) vs. 0.85 (0.76-0.94)<br><45 y: 0.77 (0.56-1.05) vs. 0.91 (0.66-1.25)	BSO/ever MHT users vs. Natural menopause/ever MHT users, Relative risk (RR, 95% CI):<br><br>Overall<br>0.79 (0.73-0.86) vs. 0.87 (0.82-0.92)<br><br>Age at menopause<br>≥45 y: 0.79 (0.72-0.86) vs. 0.86 (0.81-0.92)<br><45 y: 0.79 (0.66-0.95) vs. 0.90 (0.74-1.09)
Olesen et al,[14] 2022	BSO ± hysterectomy vs. age-adjusted controls without iatrogenic menopause, Absolute risk (n/N):<br><br>Stratified by MHT use<br>BSO/MHT users: n=33<br>Non-BSO/MHT users: n=220<br>BSO/non-MHT use: n=17<br>Non-BSO/non-MHT use: n=487<br><br>BSO ± hysterectomy vs. age-adjusted controls without iatrogenic menopause, Incident risk (MRR, 95% CI):<br><br>Stratified by MHT use<br>Non-MHT users: 1.38 (0.80-2.38)<br>MHT users: 1.28 (0.80-2.05)	NR
Rocca et al,[6] 2006	NR	BSO, ≤ 45y/MHT users vs. age-adjusted controls without iatrogenic menopause:<br>Absolute risk (n/N): 10/45 vs. 229/1,417<br>Incident Risk (HR, 95% CI): 1.27 (0.67-2.39)

© 2026 Armeni E. *JAMA Network Open*. This is an open access article distributed under the terms of the CC-BY-NC-ND License, which does not permit alteration or commercial use, including those for text and data mining, AI training, and similar technologies.

Study (year)	CVD or CHD or Stroke morbidity/mortality– Absolute / Incident or Relative Risk	All-Cause Mortality – Absolute (prevalence %, n/N) /. Incident or Relative Risk (MRR, 95% CI)
Wilson et al,[13] 2019	NR	MHT users, Absolute risk (n/N):<br>BSO+hysterectomy: 54/736<br>Hysterectomy-only: 74/1,004<br>No surgery: 162/1,972<br><br>MHT-users vs. No surgery/non-MHT users, Incident Risk (HR, 95%CI):<br>BSO+hysterectomy: 1.21 (0.92-1.61)<br>Hysterectomy-only: 1.22 (0.95-1.56)<br>No surgery: 1.37 (1.15-1.63)
Xing et al,[2] 2024	NR	BSO/ MHT users, incident risk (HR, 95%CI):<br><br>Age stratified<br>< 40 y: 1.73 (1.26-2.37)<br>40-44 y: 1.34 (0.99-1.81)<br>45-54 y (reference)<br>≥ 55 y: 1.09 (0.70-1.71)<br><br>BSO/ non-MHT users, incident risk (HR, 95%CI):<br>< 40 y: 1.01 (0.72-1.42)<br>40-44 y: 0.87 (0.56-1.37)<br>45-54 y (reference)<br>≥ 55 y: 0.71 (0.39-1.26)
Xu et al,[3] 2022	MHT users vs. non-MHT users, Incident risk (HR, 95% CI):<br><br>Natural menopause, age stratified<br><45 y: 1.03 (0.71-1.49)<br>45-49 y: 0.90 (0.70-1.17)<br>≥ 50 y: 1.02 (0.86-1.22)<br><br>Hysterectomy-only, age stratified<br><45 y: 1.00 (0.70-1.43)<br>45-49 y: 1.02 (0.39-2.67)<br>≥ 50 y: 0.21 (0.06-0.82)<br><br>Surgical menopause, age stratified<br><45 y: 0.75 (0.35-1.64)<br>45-49 y: 0.95 (0.32-2.80)<br>≥ 50 y: 0.58 (0.25-1.37)	MHT users vs. non-MHT users, Incident risk (HR, 95% CI):<br><br>Natural menopause, age stratified<br><45 y: 0.79 (0.65-0.97)<br>45-49 y: 0.89 (0.77-1.02)<br>≥ 50 y: 0.87 (0.80–0.95)<br><br>Hysterectomy-only, age stratified<br><45 y: 0.90 (0.74-1.10)<br>45-49 y: 0.92 (0.63-1.34)<br>≥ 50 y: 0.66 (0.39-1.12)<br><br>Surgical menopause, age stratified<br><45 y: 0.67 (0.45-0.99)<br>45-49 y: 0.74 (0.47-1.15)<br>≥ 50 y: 0.80 (0.55-1.16)
Zhu et al,[15] 2020	**CVD Events**<br>BSO ± hysterectomy/MHT users vs. Natural menopause/non-MHT users, Incident risk (HR, 95% CI):<br>1.06 (0.98-1.15)<br><br>MHT users vs. non-MHT users, Incident risk (HR, 95% CI):<br>BSO ± hysterectomy, age stratified:<br>< 40 y: 1.64 (1.41-1.91) vs. 2.00 (1.79-2.25)<br>40-44 y: 1.28 (1.09-1.51) vs. 1.49 (1.32-1.68)<br>45-49 y: 1.19 (1.02-1.40) vs. 1.01 (0.91-1.13)<br>50-54 y: 0.78 (0.65-0.95) vs. 0.83 (0.73-0.95)	NR

© 2026 Armeni E. *JAMA Network Open*. This is an open access article distributed under the terms of the CC-BY-NC-ND License, which does not permit alteration or commercial use, including those for text and data mining, AI training, and similar technologies.

Study (year)	CVD or CHD or Stroke morbidity/mortality– Absolute / Incident or Relative Risk	All-Cause Mortality – Absolute (prevalence %, n/N) /. Incident or Relative Risk (MRR, 95% CI)
	≥ 55 y: 0.59 (0.44-0.79) vs. 0.71 (0.57-0.88)<br><br>Natural menopause, MHT use vs non-use, age-stratified:<br>< 40 y: 1.41 (1.21-1.65) vs. 1.42 (1.18-1.71)<br>40-44 y: 1.13 (1.03-1.24) vs. 1.31 (1.18-1.46)<br>45-49 y: 0.98 (0.91-1.04) vs. 1.08 (0.99-1.16)<br>50-54 y: 0.85 (0.80 – 0.90) vs. Reference<br>≥ 55 y: 0.87 (0.80-0.93) vs. 0.91 (0.83-1.00)<br><br>**CHD Events**<br>BSO ± hysterectomy/MHT users vs. Natural menopause/non-MHT users, Incident risk (HR, 95% CI):<br>1.08 (0.98-1.18)<br><br>MHT users vs. non-MHT users, Incident risk (HR, 95% CI):<br>BSO ± hysterectomy, age stratified:<br>< 40 y: 1.53 (1.29-1.83) vs. 2.01 (1.76-2.30)<br>40-44 y: 1.29 (1.07-1.54) vs. 1.48 (1.28-1.71)<br>45-49 y: 1.17 (0.98-1.39) vs. 1.00 (0.88-1.14)<br>50-54 y: 0.76 (0.61-0.94) vs. 0.83 (0.71-0.97)<br>≥ 55 y: 0.53 (0.37-0.75) vs. 0.76 (0.59-0.98)<br><br>Natural menopause, age-stratified:<br>< 40 y: 1.44 (1.21-1.71) vs. 1.21 (0.96-1.54)<br>40-44 y: 1.08 (0.97-1.20) vs. 1.32 (1.16-1.50)<br>45-49 y: 0.95 (0.88-1.03) vs. 1.04 (0.95-1.15)<br>50-54 y: 0.81 (0.76-0.87) vs. Reference<br>≥ 55 y: 0.85 (0.78-0.93) vs. 0.85 (0.77-0.95)<br><br>**Stroke Events**<br>BSO ± hysterectomy/MHT users vs. Natural menopause/non-MHT users, Incident risk (HR, 95% CI):<br>1.07 (0.97-1.19)<br><br>MHT users vs. non-MHT users, Incident risk (HR, 95% CI):<br>BSO ± hysterectomy, age stratified:<br>< 40 y: 1.89 (1.43-2.50) vs. 1.97 (1.61-2.41)<br>40-44 y: 1.39 (1.02-1.90) vs. 1.43 (1.16-1.76)<br>45-49 y: 1.26 (0.95-1.68) vs. 1.08 (0.89-1.30)<br>50-54 y: 0.96 (0.70-1.31) vs. 0.86 (0.68-1.08)<br>≥ 55 y: 0.64 (0.38-1.09) vs. 0.58 (0.38-0.90)<br><br>Natural menopause, age-stratified:<br>< 40 y: 1.44 (1.09-1.91) vs. 1.73 (1.32-2.28)<br>40-44 y: 1.20 (1.03-1.41) vs. 1.25 (1.04-1.50)<br>45-49 y: 0.98 (0.87-1.10) vs. 1.11 (0.98-1.27)<br>50-54 y: 0.90 (0.82-1.00) vs. Reference	

© 2026 Armeni E. *JAMA Network Open*. This is an open access article distributed under the terms of the CC-BY-NC-ND License, which does not permit alteration or commercial use, including those for text and data mining, AI training, and similar technologies.

Study (year)	CVD or CHD or Stroke morbidity/mortality– Absolute / Incident or Relative Risk	All-Cause Mortality – Absolute (prevalence %, n/N) /. Incident or Relative Risk (MRR, 95% CI)
	≥ 55 y: 0.88 (0.77-1.01) vs. 1.02 (0.88-1.18)	

Abbreviations: BRCA, breast cancer susceptibility gene; BRCA-RRSO, risk-reducing salpingo-oophorectomy in BRCA mutation carriers; BSO, bilateral salpingo-oophorectomy; CHD, coronary heart disease; CI, confidence interval; CVD, cardiovascular disease; HR, hazard ratio; MHT, menopausal hormone therapy; MRR, mortality rate ratio; n/N, number of events/total population; NR, not reported; RR, relative risk; y, years.

© 2026 Armeni E. *JAMA Network Open*. This is an open access article distributed under the terms of the CC-BY-NC-ND License, which does not permit alteration or commercial use, including those for text and data mining, AI training, and similar technologies.

eTable 14. Cardiovascular risk stratification of participants at baseline for all studies.

Author	Age at surgery / menopause	Body mass index	Central adiposity	Hypertension	Smoking	Diabetes / Glucose	Dyslipidemia	Cardiometabolic risk phenotype
Abbas et al,[26] 2018	Moderate	Moderate	Moderate	Moderate	Low	Moderate	moderate	Intermediate
Abildgaard et al,[16] 2018	Moderate	Moderate	Moderate	Moderate	Moderate	Moderate	Moderate	Low–Intermediate
Allison et al,[17] 2008	Moderate	High	High	High	High	Moderate	Moderate -to-high	Intermediate–High
Poorthuis et al,[9] 2022	High	Low	Low	Low-Moderate	Low	Moderate	High	Low–Intermediate
Chen et al,[36] 2023	Moderate	Moderate	Moderate	Moderate	Low	Low	Moderate	Low–Intermediate
Duan et al,[20] 2012	High	Moderate	Moderate	High	High	High	High	Intermediate–High
Do Valle et al,[19] 2021	Low	NA	Low	Low	NA	Low	NA	Low
Dam et al,[18] 2019	High	Moderate	Moderate	High	High	Moderate	High	Intermediate–High
Farahmand et al,[21] 2014	High	High	High	High	Moderate	High	High	High
Farland et al,[11] 2023	High	High	High	High	High	High	High	Intermediate–High
Gierach et al,[10] 2014	High	Moderate	Moderate	High	High	High	High	Intermediate–High
Dorum et al,[27] 2008	High	High	High	High	High	Moderate	High	High
Honigberg et al,[12] 2019	High	Moderate	Moderate	High	High	High	High	High
Howard et al,[22] 2005	High	High	High	High	Moderate	Moderate-high	High	Intermediate–High
Indhavivadhana et al,[28] 2011	High	Moderate	High	Moderate	Low	Moderate	Moderate	Intermediate
Jamali et al,[29] 2024	Moderate	High	High	High	Low	High	High	High

© 2026 Armeni E. *JAMA Network Open*. This is an open access article distributed under the terms of the CC-BY-NC-ND License, which does not permit alteration or commercial use, including those for text and data mining, AI training, and similar technologies.

Author	Age at surgery / menopause	Body mass index	Central adiposity	Hypertension	Smoking	Diabetes / Glucose	Dyslipidemia	Cardiometabolic risk phenotype
Appiah et al,[4] 2020	Moderate	Moderate	High	High	Moderate	High	moderate	Intermediate–High
Appiah et al,[5] 2015	High	High	High	High	Moderate	Moderate-high	NA	Intermediate–High
Ingelsson et al,[1] 2010	NA	NA	NA	NA	NA	NA	NA	NA
Krul et al,[23] 2018	Moderate	Moderate	N/A	Low-Moderate	Low	Moderate	Moderate	Intermediate
Mack et al,[30] 2004	Moderate	Moderate	Moderate	Low-Moderate	Low	Low	moderate	Low–Intermediate
Michelsen et al,[8] 2023	Moderate	Moderate	Moderate	Moderate	Low	Low	Moderate	Low–Intermediate
Netterlid et al,[31] 2021	Moderate	Low	Moderate	Low-Moderate	Low	Moderate	moderate	Intermediate
Olesen et al,[14] 2022	Moderate	Moderate-high	Moderate	Moderate-high	Low-moderate	Moderate	Low-moderate	Low–Intermediate
Ou et al,[32] 2023	High	High	High	High	Moderate	High	High	High
Ozdemir et al,[33] 2009	High	Moderate	High	High	Low	High	High	High
Ozkaya et al,[34] 2011	High	Moderate	Moderate	High	Low	Moderate	moderate	Intermediate–High
Rivera et al,[7] 2009	High	Moderate	Moderate	Moderate	Moderate	Moderate	High	Intermediate–High
Rocca et al,[6] 2006	High	Moderate	Moderate	Moderate	Moderate	Moderate	High	Intermediate–High
Thong et al,[24] 2022	High	Moderate	Moderate	Moderate	Moderate	Moderate	High	Intermediate–High
Tuesley et al,[25] 2020	High	Moderate	Moderate	Moderate	Moderate	Moderate	Moderate-high	Intermediate–High
Tuysuzoglu et al,[35] 2020	Moderate	Moderate	Moderate-high	High	Low	Moderate	Moderate-high	High
Wilson et al,[13] 2019	High	High	Moderate-high	Moderate-high	Moderate	Moderate	Moderate-high	Intermediate
Xing et al,[2] 2024	High	High	High	High	Moderate-high	High	Moderate-high	Intermediate–High
Xu et al,[3] 2022	High	High	High	Moderate-high	Moderate	Moderate-high	Moderate-high	Intermediate–High

© 2026 Armeni E. *JAMA Network Open*. This is an open access article distributed under the terms of the CC-BY-NC-ND License, which does not permit alteration or commercial use, including those for text and data mining, AI training, and similar technologies.

Author	Age at surgery / menopause	Body mass index	Central adiposity	Hypertension	Smoking	Diabetes / Glucose	Dyslipidemia	Cardiometabolic risk phenotype
Zhu et al,[15] 2020	High	moderate	Moderate	Moderate	Moderate	Low	Low-moderate	Low–Intermediate

Abbreviations: NA, not available.

Studies were categorized into low, intermediate, and high cardiometabolic risk phenotypes based on study-level baseline characteristics (adiposity, blood pressure, glycemic status, lipid profile, and smoking prevalence), using a qualitative domain-based approach to harmonize heterogeneous reporting.

The distribution of studies across phenotypes illustrates clustering of cardiometabolic burden, with most cohorts falling within intermediate to high-risk categories. Higher-risk phenotypes were more frequently characterized by earlier age at iatrogenic menopause, greater prevalence of obesity and central adiposity, hypertension, diabetes, and dyslipidemia.

© 2026 Armeni E. *JAMA Network Open*. This is an open access article distributed under the terms of the CC-BY-NC-ND License, which does not permit alteration or commercial use, including those for text and data mining, AI training, and similar technologies.

eTable 15. Qualitative summary of cardiovascular and mortality outcomes after surgical menopause, stratified by baseline cardiovascular risk

Study	Exposure	Outcome(s)	Direction of Association	Overall pattern
Low or Low–Intermediate Baseline Cardiovascular Risk				
Abildgaard et al,[16] 2018	Prophylactic BSO	All-cause mortality	= Mortality risk	Subclinical vascular changes predominate; no consistent increase in CVD, CHD, stroke, or mortality.
Chen et al,[36] 2023	Hysterectomy + BSO	CIMT progression	↑ Subclinical vascular risk (no events)	
Do Valle et al,[19] 2021	RRBSO, BRCA positive	CVD,CHD, stroke events	= (slightly ↑ when compared to intact ovaries) in MHT users	
Mack et al,[30] 2004	Hysterectomy + BSO	CIMT	↑ Subclinical vascular risk (lost after adjustment for menopausal years)	
Michelsen et al,[8] 2023	BSO ± Hysterectomy	CVD and all-cause mortality	= CVD mortality<br>↑ all-cause mortality, especially <40 y	
Olesen et al,[14] 2022	BSO ± Hysterectomy	CVD mortality	= (similar for MHT users)	
Poorthuis et al,[9] 2022	Hysterectomy+BSO	CVD, CHD, stroke events	↑ CVD event risk (remained significant after age stratification)	
Zhu et al,[15] 2020	BSO ± hysterectomy	CVD, CHD, stroke events	↑ CVD, CHD event risk <50y, (= risk ≥50y)<br>↑ CVD, CHD event risk in MHT users	
Intermediate or Intermediate–High Baseline Cardiovascular Risk				
Abbas et al,[26] 2018	Hysterectomy + BSO	Subclinical vascular (cAP)	↓	
Allison et al,[17] 2008	BSO	Subclinical vascular (CAC score)	↑ but non-significant trend	
Appiah et al,[5] 2015	BSO	CVD, all-cause mortality	↑ CVD mortality only in diabetic patients, ↑ all-cause mortality	
Appiah et al,[4] 2020	BSO	CVD, HD, all-cause mortality	↑ CVD, HD and all-cause mortality especially <45 y	
Duan et al,[20] 2012	BSO	CVD mortality, all-cause mortality	= CVD and all-cause mortality (even after adjusted for age and MHT)	
Farland et al,[11] 2023	Hysterectomy+BSO	CVD, CHD, stroke events	↑ CVD, CHD, stroke risk, stronger ≤50 y	
Dam et al,[18] 2019	Hysterectomy+BSO(/USO)	CHD event	↑ in the age-adjusted model; attenuated to the null after adjustment for confounders and for age at menopause	
Gierach et al,[10] 2014	BSO	CHD, stroke and all-cause mortality	↑ CHD mortality if ≤ 50y<br>= stroke mortality<br>↑ all-cause mortality if ≤35 y<br>= all-cause mortality overall	

© 2026 Armeni E. *JAMA Network Open*. This is an open access article distributed under the terms of the CC-BY-NC-ND License, which does not permit alteration or commercial use, including those for text and data mining, AI training, and similar technologies.

Study	Exposure	Outcome(s)	Direction of Association	Overall pattern
Howard et al,[22] 2005	Hysterectomy + BSO	CVD, stroke, CHF events, CVD mortality	↑ CVD, stroke, CHF events<br>= CVD mortality	Clear increase in CVD/CHD/stroke and selective increases in mortality, especially when surgery occurs before 45–50 years.
Indhavivadhana et al,[28] 2011	BSO ± Hysterectomy	Metabolic syndrome	= risk	
Krul et al,[23] 2018	Cancer treatment-induced POI	CVD and IHD event	= risk	
Netterlid et al,[31] 2021	Ovarian failure after childhood cancer therapy	Metabolic syndrome event	↑ risk	
Ozkaya et al,[34] 2011	Hysterectomy + BSO	Subclinical vascular (CIMT)	↑ Subclinical vascular risk (no events)	
Rivera et al,[7] 2009	BSO	CVD mortality	= risk	
Rocca et al,[6] 2006	BSO	CVD, all-cause mortality	= CVD mortality<br>↑ all-cause mortality if <45y, otherwise =	
Tuesley et al,[25] 2020	Hysterectomy + BSO	CVD, all-cause mortality	= CVD mortality<br>↑ all-cause mortality if <35–44y; = overall	
Wilson et al,[13] 2019	Hysterectomy + BSO	All-cause mortality	= all-cause mortality overall; ↑ in non-MHT users only	
Thong et al,[24] 2022	BSO	CVD, stroke, CVD mortality, all-cause mortality	↓ CVD events<br>= stroke events<br>= CVD mortality<br>↑ all-cause mortality	
Xu et al,[3] 2022	BSO	CVD, all-cause mortality	↑ CVD mortality if <40–44y; = older ages<br>↑ all-cause mortality if ≤49y	
Xing et al,[2] 2024	BSO	All-cause mortality	↑ all-cause mortality if <40y	
High Baseline Cardiovascular Risk				
Farahmand et al,[21] 2014	BSO ± Hysterectomy	Metabolic syndrome	= risk	Clear increase in CVD, stroke, metabolic syndrome, CHF, and cardiovascular & all-cause mortality, especially at younger surgical ages.
Dorum et al,[27] 2008	BSO ± Hysterectomy	Framingham score, stroke, metabolic syndrome	↑ Subclinical, ↑ stroke, ↑ metabolic syndrome	
Honigberg et al,[12] 2019	BSO <40	IHD, CHD, CHF, stroke events	↑↑ IHD, CHD, stronger if <40y<br>↑ CHF if <40 y<br>= stroke	
Jamali et al,[29] 2024	Induced menopause	CVD, CHD	=	
Ozdemir et al,[33] 2009	Hysterectomy + BSO	Metabolic syndrome	=	
Ou et al,[32] 2023	BSO ± hysterectomy	Metabolic syndrome	↑	
Tuysuzoglu et al,[35] 2020	BSO	Metabolic syndrome	↑	

© 2026 Armeni E. *JAMA Network Open*. This is an open access article distributed under the terms of the CC-BY-NC-ND License, which does not permit alteration or commercial use, including those for text and data mining, AI training, and similar technologies.

Abbreviations: BRCA, breast cancer susceptibility gene; BSO, bilateral salpingo-oophorectomy; CAC, coronary artery calcium; cAP, central aortic augmentation pressure; CHD, coronary heart disease; CHF, congestive heart failure; CIMT, carotid intima-media thickness; CVD, cardiovascular disease; IHD, ischemic heart disease; MHT, menopausal hormone therapy; POI, premature ovarian insufficiency; RRBSO, risk-reducing bilateral salpingo-oophorectomy; USO, unilateral salpingo-oophorectomy; y, years. Symbols: "=", equivalent; "↑", higher; "↓", lower.

The study conducted by Ingelsson et al,[1] 2010 was not included due to insufficient data for baseline cardiovascular risk categorization.

© 2026 Armeni E. *JAMA Network Open*. This is an open access article distributed under the terms of the CC-BY-NC-ND License, which does not permit alteration or commercial use, including those for text and data mining, AI training, and similar technologies.

eTable 16. Leave-one-out sensitivity analysis of the pooled estimates, by outcome

Cohort omitted	Estimates	HR (95% CI)	Test for overall effect	Heterogeneity: *Q* (df), *P*	I^2
**Composite cardiovascular events — primary model, 7 cohorts, 1.25 (1.12-1.39), I^2 = 86%**					
do Valle et al,[19] 2021	6	1.23 (1.10-1.38)	*Z* = 3.71; *P* < .001	23.54 (5), <.001	88%
Farland et al,[11] 2023	6	1.21 (1.17-1.25)	*Z* = 10.86; *P* < .001	7.99 (5), .16	0%
Howard et al,[22] 2005	6	1.24 (1.08-1.43)	*Z* = 2.98; *P* = .003	24.41 (5), <.001	90%
Ingelsson et al,[1] 2010	6	1.26 (1.11-1.45)	*Z* = 3.42; *P* = .001	24.13 (5), <.001	90%
Krul et al,[23] 2018	6	1.28 (1.16-1.41)	*Z* = 4.82; *P* < .001	20.86 (5), <.001	85%
Poorthuis et al,[9] 2022	6	1.26 (1.10-1.45)	*Z* = 3.26; *P* = .001	22.66 (5), <.001	86%
Zhu et al,[15] 2020	6	1.25 (1.08-1.45)	*Z* = 3.05; *P* = .002	25.01 (5), <.001	86%
**Coronary heart disease — primary model, 6 cohorts, 1.48 (1.07-2.03), I^2 = 97%**					
Dam et al,[18] 2019	5	1.55 (1.04-2.30)	*Z* = 2.17; *P* = .03	40.23 (4), <.001	98%
Farland et al,[11] 2023	5	1.45 (0.97-2.15)	*Z* = 1.82; *P* = .07	27.43 (4), <.001	98%
Honigberg et al,[12] 2019	5	1.28 (1.12-1.46)	*Z* = 3.55; *P* < .001	16.83 (4), .002	85%
Ingelsson et al,[1] 2010	5	1.57 (1.07-2.30)	*Z* = 2.33; *P* = .02	38.66 (4), <.001	98%
Poorthuis et al,[9] 2022	5	1.56 (1.06-2.31)	*Z* = 2.25; *P* = .02	36.20 (4), <.001	97%
Zhu et al,[15] 2020	5	1.54 (1.03-2.30)	*Z* = 2.11; *P* = .03	40.34 (4), <.001	96%
**Stroke — primary model, 4 cohorts, 1.22 (1.15-1.28), I^2 = 0%**					
Farland et al,[11] 2023	3	1.20 (1.13-1.26)	*Z* = 6.34; *P* < .001	0.17 (2), .92	0%
Ingelsson et al,[1] 2010	3	1.22 (1.15-1.29)	*Z* = 6.82; *P* < .001	3.99 (2), .14	0%
Poorthuis et al,[9] 2022	3	1.25 (1.14-1.37)	*Z* = 4.86; *P* < .001	3.27 (2), .19	30%
Zhu et al,[15] 2020	3	1.24 (1.11-1.39)	*Z* = 3.87; *P* < .001	4.00 (2), .14	49%
**Cardiovascular mortality — primary model, 7 estimates from 6 cohorts, 1.03 (0.94-1.13), I^2 = 40%**					
Appiah et al,[5] 2015	6	1.01 (0.90-1.14)	*Z* = 0.18; *P* = .86	9.75 (5), .08	46%
Appiah et al,[4] 2020	6	1.03 (0.92-1.16)	*Z* = 0.52; *P* = .61	11.00 (5), .05	53%
Michelsen et al,[8] 2023	6	1.01 (0.91-1.13)	*Z* = 0.21; *P* = .83	9.80 (5), .08	46%

© 2026 Armeni E. *JAMA Network Open*. This is an open access article distributed under the terms of the CC-BY-NC-ND License, which does not permit alteration or commercial use, including those for text and data mining, AI training, and similar technologies.

Cohort omitted	Estimates	HR (95% CI)	Test for overall effect	Heterogeneity: *Q* (df), *P*	I^2
Rivera et al,[7] 2009	6	1.06 (0.96-1.16)	*Z* = 1.17; *P* = .24	8.60 (5), .13	31%
Rocca et al,[6] 2006	6	1.05 (0.96-1.15)	*Z* = 1.10; *P* = .27	7.95 (5), .16	35%
Tuesley et al,[25] 2020	6	1.05 (0.94-1.17)	*Z* = 0.88; *P* = .38	9.26 (5), .10	41%
Xu et al,[3] 2022	6	1.01 (0.92-1.11)	*Z* = 0.16; *P* = .87	8.19 (5), .15	32%
**All-cause mortality — primary model, 9 cohorts, 1.08 (1.01-1.16), I^2 = 79%**					
Appiah et al,[5] 2015	8	1.08 (1.00-1.17)	*Z* = 2.08; *P* = .04	36.53 (7), <.001	81%
Appiah et al,[4] 2020	8	1.09 (1.01-1.18)	*Z* = 2.27; *P* = .02	38.81 (7), <.001	82%
Gierach et al,[10] 2014	8	1.10 (1.03-1.18)	*Z* = 2.64; *P* = .008	31.61 (7), <.001	74%
Michelsen et al,[8] 2023	8	1.07 (1.00-1.14)	*Z* = 1.95; *P* = .05	31.75 (7), <.001	78%
Rocca et al,[6] 2006	8	1.09 (1.01-1.17)	*Z* = 2.28; *P* = .02	38.81 (7), <.001	83%
Tuesley et al,[25] 2020	8	1.11 (1.04-1.18)	*Z* = 3.16; *P* = .002	27.02 (7), <.001	69%
Wilson et al,[13] 2019	8	1.07 (1.00-1.15)	*Z* = 2.10; *P* = .04	35.57 (7), <.001	80%
Xing et al,[2] 2024	8	1.08 (1.01-1.17)	*Z* = 2.09; *P* = .04	37.28 (7), <.001	82%
Xu et al,[3] 2022	8	1.07 (1.00-1.14)	*Z* = 1.89; *P* = .06	26.67 (7), <.001	76%

In each row the named cohort is omitted and the remaining estimates are re-pooled. All analyses use the random-effects model with the between-study variance estimated by restricted maximum likelihood and Wald confidence intervals, consistent with the primary analysis for each outcome. I^2 is calculated as $\tau^2/(\tau^2 + s^2)$, as in the random-effects figures.

The composite cardiovascular events and stroke estimates are robust, excluding the null in every iteration. Coronary heart disease is not: it becomes non-significant on omission of Farland et al,[11] 2023 (1.45; 95% CI, 0.97-2.15). Heterogeneity in composite cardiovascular events is attributable to a single cohort rather than being diffuse, omission of Farland et al,[11] 2023 reducing I^2 from 86% to 0% with a residual *Q* of 7.99 (df = 5; *P* = .16). No comparable attribution holds for coronary heart disease, where omission of Honigberg et al,[12] 2019 leaves substantial residual heterogeneity (I^2 = 85%).

All-cause mortality is the least robust estimate. It falls to 1.07 (95% CI, 1.00-1.14) and loses conventional significance on omission of Xu et al,[3] 2022 (*P* = .06), and sits on the conventional threshold on omission of Michelsen et al,[8] 2023 (*P* = .05). Omission of Xing et al,[2] 2024, the other cohort using a natural-menopause referent, leaves the estimate significant (1.08; 95% CI, 1.01-1.17; *P* = .04), so the significance of the pooled estimate is not simply a function of comparator definition.

Cardiovascular mortality remains non-significant in every iteration.

For stroke, omission of Ingelsson et al,[1] 2010 and omission of Zhu et al,[15] 2020 give almost identical Cochran *Q* (3.99 and 4.00, both df = 2) but I^2 of 0% and 49%. This is not an inconsistency. Restricted maximum likelihood does not estimate τ^2 from *Q* and df alone: it solves an estimating equation weighted by the squared inverse-variance weights, so τ^2 depends on how the observed dispersion is distributed across estimates of differing precision. In the first set the dispersion is carried almost entirely by the least precise estimate and is what within-study sampling error alone would predict, and τ^2 is returned at its boundary value of 0; in the second it is spread across estimates of more similar precision and τ^2 is 0.0048. Under the method of DerSimonian and Laird both sets would give I^2 of approximately 50%.

The 7 cardiovascular mortality estimates arise from 6 independent cohorts (eTable 17). No iteration removes that source population in full, since omission of either report leaves the other in the model; the analysis therefore tests the influence of individual reports rather than of individual source populations. All values were independently reproduced from the underlying log[HR] and standard error inputs.

Abbreviations: CI, confidence interval; df, degrees of freedom; HR, hazard ratio.

© 2026 Armeni E. *JAMA Network Open*. This is an open access article distributed under the terms of the CC-BY-NC-ND License, which does not permit alteration or commercial use, including those for text and data mining, AI training, and similar technologies.

eTable 17. Sensitivity analysis for the overlapping reports of the Olmsted County cohort, cardiovascular mortality

Analysis	Estimates	HR (95% CI)	*P* Value	τ^2	Heterogeneity: *Q* (df), *P* Value	I^2
Both Olmsted County reports included (primary analysis, Figure 3A)	7	1.03 (0.94-1.13)	.51	0.006	11.03 (6), .09	40%
Rivera et al,[7] 2009 retained, Rocca et al,[6] 2006 excluded	6	1.05 (0.96-1.15)	.27	0.004	7.95 (5), .16	35%
Rocca et al,[6] 2006 retained, Rivera et al,[7] 2009 excluded	6	1.06 (0.96-1.16)	.24	0.004	8.60 (5), .13	31%
Both Olmsted County reports excluded	5	1.08 (0.98-1.18)	.10	0.003	5.18 (4), .27	27%

Rocca et al,[6] 2006 and Rivera et al,[7] 2009 report cardiovascular mortality from the same Olmsted County cohort, with the same 1,091 exposed and 2,383 comparator women. Both are entered in the primary analysis shown in Figure 3A, where they carry a combined 16.9% of the pooled weight (Rivera et al,[7] 2009, 11.4%; Rocca et al,[6] 2006, 5.5%), so that population is counted twice. The primary analysis therefore comprises 7 estimates arising from 6 cohorts, which is why the column is headed “Estimates” rather than “Cohorts.”
All analyses use the random-effects model with the between-study variance estimated by restricted maximum likelihood and Wald confidence intervals. I^2 is calculated here as $\tau^2/(\tau^2 + s^2)$, and in the fixed-effect sensitivity analysis in eTable 9 as $(Q - \text{df})/Q$. Under the DerSimonian-Laird estimator these 2 expressions are algebraically identical; the values differ between the 2 tables because the random-effects analyses use restricted maximum likelihood rather than DerSimonian-Laird, that is, because of the estimator and not because of the formula.
The pooled estimate is not materially altered by any of these configurations and remains non-significant in all 4. Removing the duplicated population moves the estimate away from the null rather than towards it (1.03 to 1.08) and reduces heterogeneity monotonically (I^2, 40% to 27%), because both Olmsted County estimates lie below unity (Rivera et al,[7] 2009, 0.87; Rocca et al,[6] 2006, 0.75).
Which of the 2 reports is retained makes almost no difference to the pooled estimate (1.05 vs 1.06), and the between-study variance is the same (τ^2 = 0.004) whichever is kept, so neither report can be said to contribute more of the between-study variance than the other. The small difference in residual I^2 between those 2 configurations (35% vs 31%) reflects the different precision of the estimate retained rather than a difference in the dispersion it introduces. Only the decision to count the cohort once or twice is consequential.
The same cohort contributes to the age-stratified analysis of cardiovascular mortality (Figure 4A) through the Rivera et al,[7] 2009 age bands only, so it is entered once there and this sensitivity analysis does not apply.
All-cause mortality carries no duplicated population: Rivera et al,[7] 2009 reports cardiovascular mortality only, so the Olmsted County cohort enters the 9 all-cause estimates through Rocca et al,[6] 2006 alone. Appiah et al,[5] 2015 and Appiah et al,[4] 2020 are treated throughout as reports of independent populations.
The leave-one-out analysis in eTable 16 does not isolate this overlap, because omitting either Olmsted County report leaves the other in the analysis; no iteration of that analysis removes the source population in full.
Abbreviations: CI, confidence interval; HR, hazard ratio

© 2026 Armeni E. *JAMA Network Open*. This is an open access article distributed under the terms of the CC-BY-NC-ND License, which does not permit alteration or commercial use, including those for text and data mining, AI training, and similar technologies.

eTable 18. Leave-one-out sensitivity analysis of the age-stratified mortality analyses

Estimate omitted	Age <45 y, HR (95% CI)	*P*	*I*^{2}	Age ≥45 y, HR (95% CI)	*P*	*I*^{2}	Subgroup *P*
**Cardiovascular mortality (Figure 4A)**							
None (primary analysis)	1.20 (1.04-1.37)	.01	0%	0.95 (0.81-1.11)	.49	61%	.03
Tuesley et al,[25] 2020 (<35 y)	1.18 (1.02-1.35)	.02	0%	—	—	—	.04
Michelsen et al,[8] 2023 (<40 y)	1.20 (1.03-1.39)	.02	0%	—	—	—	.03
Tuesley et al,[25] 2020 (35-44 y)	1.19 (1.01-1.40)	.04	0%	—	—	—	.05
Rivera et al,[7] 2009 (<45 y)	1.19 (1.03-1.38)	.02	0%	—	—	—	.03
Appiah et al,[5] 2015 (≤45 y)	1.25 (1.05-1.50)	.01	0%	—	—	—	.02
Michelsen et al,[8] 2023 (40-52 y)	—	—	—	0.94 (0.78-1.13)	.50	67%	.04
Rivera et al,[7] 2009 (45-50 y)	—	—	—	0.98 (0.84-1.14)	.77	59%	.05
Appiah et al,[5] 2015 (>45 y)	—	—	—	0.91 (0.76-1.09)	.33	60%	.02
Tuesley et al,[25] 2020 (45-54 y)	—	—	—	0.95 (0.79-1.14)	.56	65%	.05
Rivera et al,[7] 2009 (>50 y)	—	—	—	0.95 (0.80-1.13)	.58	66%	.04
Michelsen et al,[8] 2023 (≥53 y)	—	—	—	0.91 (0.80-1.03)	.15	32%	.004
Tuesley et al,[25] 2020 (55-64 y)	—	—	—	0.95 (0.79-1.14)	.58	66%	.04
Tuesley et al,[25] 2020 (≥65 y)	—	—	—	0.98 (0.85-1.14)	.83	55%	.05
**All-cause mortality (Figure 4B)**							
None (primary analysis)	1.15 (1.06-1.25)	<.001	59%	0.99 (0.89-1.09)	.77	85%	.02
Tuesley et al,[25] 2020 (<35 y)	1.13 (1.05-1.21)	<.001	48%	—	—	—	.03
Gierach et al,[10] 2014 (≤35 y)	1.16 (1.05-1.28)	.004	66%	—	—	—	.03
Michelsen et al,[8] 2023 (<40 y)	1.14 (1.05-1.24)	.001	60%	—	—	—	.02
Gierach et al,[10] 2014 (36-40 y)	1.18 (1.08-1.29)	<.001	59%	—	—	—	.008
Tuesley et al,[25] 2020 (35-44 y)	1.16 (1.05-1.28)	.004	67%	—	—	—	.02
Rocca et al,[6] 2006 (<45 y)	1.13 (1.05-1.21)	<.001	49%	—	—	—	.03

© 2026 Armeni E. *JAMA Network Open*. This is an open access article distributed under the terms of the CC-BY-NC-ND License, which does not permit alteration or commercial use, including those for text and data mining, AI training, and similar technologies.

Estimate omitted	Age <45 y, HR (95% CI)	*P*	I^2	Age ≥45 y, HR (95% CI)	*P*	I^2	Subgroup *P*
Gierach et al,[10] 2014 (41-45 y)	1.18 (1.08-1.29)	<.001	55%	—	—	—	.008
Appiah et al,[5] 2015 (≤45 y)	1.18 (1.07-1.30)	.001	67%	—	—	—	.01
Michelsen et al,[8] 2023 (40-52 y)	—	—	—	0.97 (0.88-1.08)	.63	86%	.01
Rocca et al,[6] 2006 (45-50 y)	—	—	—	0.98 (0.88-1.09)	.76	87%	.02
Appiah et al,[5] 2015 (>45 y)	—	—	—	0.97 (0.87-1.08)	.60	85%	.01
Tuesley et al,[25] 2020 (45-54 y)	—	—	—	1.01 (0.92-1.11)	.78	79%	.04
Gierach et al,[10] 2014 (46-50 y)	—	—	—	0.99 (0.89-1.11)	.86	85%	.03
Rocca et al,[6] 2006 (>50 y)	—	—	—	0.99 (0.89-1.10)	.89	87%	.03
Gierach et al,[10] 2014 (≥51 y)	—	—	—	0.99 (0.89-1.11)	.90	83%	.03
Michelsen et al,[8] 2023 (≥53 y)	—	—	—	0.95 (0.88-1.02)	.17	72%	<.001
Tuesley et al,[25] 2020 (55-64 y)	—	—	—	1.00 (0.89-1.11)	.96	86%	.04
Tuesley et al,[25] 2020 (≥65 y)	—	—	—	1.00 (0.90-1.11)	.94	87%	.03

In each row a single age-band estimate is omitted and both subgroups are refitted under the random-effects model, with the between-study variance estimated by restricted maximum likelihood, Wald confidence intervals and I^2 calculated as $\tau^2/(\tau^2 + s^2)$, consistent with the primary analysis. An em dash indicates that the subgroup is unchanged from the primary analysis. Rows below 45 years are ordered by the upper limit of the omitted band and rows at or above 45 years by its lower limit. The single band spanning the cutoff (Michelsen et al,[8] 2023, 40-52 years) is assigned to the group at or above 45 years by its midpoint, as in Figures 4A and 4B; the row omitting that band therefore reproduces the alternative configuration in which spanning bands are excluded. The cardiovascular mortality analysis comprises 13 estimates from 4 cohorts (5 below and 8 at or above 45 years) and the all-cause analysis 18 estimates from 5 cohorts (8 and 10 respectively).

Both subgroup estimates are robust. Below 45 years the pooled estimate ranges from 1.18 to 1.25 for cardiovascular mortality and 1.13 to 1.18 for all-cause mortality, excluding the null in every iteration; at or above 45 years it ranges from 0.91 to 0.98 and 0.95 to 1.01 respectively, including the null in every iteration.

The subgroup-difference *P* value is shown for completeness. It treats age bands as independent and is anti-conservative; it crosses the conventional threshold in 2 of the 13 iterations for cardiovascular mortality, on omission of the 45-50 year and 65 year or older bands (*P* = .054 in both), while remaining below it in all 18 iterations for all-cause mortality. Two further cardiovascular mortality iterations lie between *P* = .04 and *P* = .05 and are shown as .05 under 2-decimal rounding. Inference on the age contrast is based instead on the cohort-clustered analysis reported in eTable 20.

Omission of the estimate for women aged 53 years or older reported by Michelsen et al,[8] 2023 is the only exclusion that materially reduces heterogeneity in the subgroup at or above 45 years, for both outcomes (I^2 falling from 61% to 32% for cardiovascular mortality and from 85% to 72% for all-cause mortality), and it produces the largest strengthening of the age contrast, from *P* = .03 to *P* = .004 for cardiovascular mortality and from *P* = .02 to *P* < .001 for all-cause mortality. This estimate is an older age band with an elevated hazard ratio and therefore runs counter to a monotonic age gradient. Seven further omissions strengthen the contrast to a lesser degree, 2 for cardiovascular mortality and 5 for all-cause mortality.

All-cause mortality estimates from the Olmsted County cohort (Rocca et al,[6] 2006) are stratified by age at prophylactic bilateral oophorectomy (537 women: 124 aged <45 years, 243 aged 45-50 years and 170 aged >50 years) rather than by age at bilateral oophorectomy for all indications (1091 women), because age-stratified estimates for the all-indication group are not reported. The cohort's pooled all-cause estimate in Figure 3B is the all-indication estimate. Cardiovascular mortality strata from the same cohort (Rivera et al,[7] 2009) are drawn from the full bilateral oophorectomy group, so the exposure definition differs between the 2 mortality outcomes for this cohort.

Referent women were individually matched by year of birth and compared within the same age stratum. The referent counts for the 3 strata (1417, 645 and 321 women) are not per-stratum matched sets of the exposed women shown above them: they partition the entire comparator group of the oophorectomy cohort (2383 women), which also includes the comparators for women who underwent unilateral oophorectomy, and are therefore larger than the corresponding exposed counts.

© 2026 Armeni E. *JAMA Network Open*. This is an open access article distributed under the terms of the CC-BY-NC-ND License, which does not permit alteration or commercial use, including those for text and data mining, AI training, and similar technologies.

Two further sensitivity analyses address the difference in exposure definition. Omitting the Olmsted County <45-year stratum alone gives a pooled estimate of 1.13 (95% CI, 1.05-1.21) below age 45 years, unchanged at 0.99 (0.89-1.09) at or above 45 years, and a subgroup difference of χ^2 = 4.83 (*P* = .03). Omitting the cohort entirely gives 1.13 (1.05-1.21) and 0.99 (0.88-1.11), χ^2 = 3.58 (*P* = .06). Substituting the prophylactic-indication estimate for the Olmsted County cohort in the overall all-cause analysis leaves the pooled estimate at 1.08 (1.01-1.16), so the mismatch is confined to the age-stratified analyses.
Consistent with the extraction rule set out in the eMethods, Tuesley et al,[25] 2020 contributes Model 1 (age as the underlying time variable) estimates to the all-cause mortality analysis in Figure 4B, for which that report publishes 2 models, and Model 2 (adjusted additionally for tubal ligation, parity, age at entry, area of residence and area-level socioeconomic status, stratified by birth cohort) estimates to the cardiovascular mortality analysis in Figure 4A, for which it publishes only 1. The adjustment set therefore differs between the 2 panels for this cohort. The same applies to the corresponding rows of this table.
Abbreviations: CI, confidence interval; HR, hazard ratio.

© 2026 Armeni E. *JAMA Network Open*. This is an open access article distributed under the terms of the CC-BY-NC-ND License, which does not permit alteration or commercial use, including those for text and data mining, AI training, and similar technologies.

eTable 19. Age bands as reported by each contributing cohort and the age interval across which each contrast is measured

Cohort	Age band as published	Assigned midpoint, y	Subgroup at the 45-y cut-off	Group mean age, <45 y / ≥45 y	Age interval, y	Ratio of HRs	Per-year equivalent
**All-cause mortality (25 age-band estimates, 7 cohorts), ordered by age interval**							
Appiah et al,[5] 2015	≤45 y	42	<45 y	42.0 / 47.5	5.5	1.03	+0.005
	>45 y	47.5	≥45 y				
Rocca et al,[6] 2006	<45 y	42	<45 y	42.0 / 50.5	8.5	0.58	−0.065
	45-50 y	47.5	≥45 y				
	>50 y	53	≥45 y				
Xing et al,[2] 2024	<40 y	37	<45 y	37.0 / 46.0	9.0	0.67	−0.044
	≥40 y	46	≥45 y				
Xu et al,[3] 2022	<40 y	37	<45 y	39.8 / 50.1	10.2	0.92	−0.008
	40-44 y	42	<45 y				
	45-49 y	47	≥45 y				
	50-52 y	51	≥45 y				
	≥53 y	56	≥45 y				
Gierach et al,[10] 2014	≤35 y	32	<45 y	38.6 / 51.7	13.1	0.88	−0.010
	36-40 y	38	<45 y				
	41-45 y	43	<45 y				
	46-50 y	48	≥45 y				
	≥51 y	54	≥45 y				
Michelsen et al,[8] 2023	<40 y	37	<45 y	37.0 / 51.2	14.2	0.96	−0.003
	40-52 y	46	≥45 y				
	≥53 y	56	≥45 y				
Tuesley et al,[25] 2020	<35 y	32	<45 y	38.2 / 54.8	16.7	0.68	−0.023
	35-44 y	39.5	<45 y				
	45-54 y	49.5	≥45 y				
	55-64 y	59.5	≥45 y				
	≥65 y	68	≥45 y				
**Cardiovascular mortality (18 age-band estimates, 5 cohorts), ordered by age interval**							
Appiah et al,[5] 2015	≤45 y	42	<45 y	42.0 / 47.5	5.5	0.98	−0.004
	>45 y	47.5	≥45 y				
Rivera et al,[7] 2009	<45 y	42	<45 y	42.0 / 50.1	8.1	0.63	−0.057
	45-50 y	47.5	≥45 y				
	>50 y	53	≥45 y				

© 2026 Armeni E. *JAMA Network Open*. This is an open access article distributed under the terms of the CC-BY-NC-ND License, which does not permit alteration or commercial use, including those for text and data mining, AI training, and similar technologies.

Cohort	Age band as published	Assigned midpoint, y	Subgroup at the 45-y cut-off	Group mean age, <45 y / ≥45 y	Age interval, y	Ratio of HRs	Per-year equivalent
Xu et al,[3] 2022	<40 y	37	<45 y	39.7 / 49.5	9.8	0.60	−0.052
	40-44 y	42	<45 y				
	45-49 y	47	≥45 y				
	50-52 y	51	≥45 y				
	≥53 y	56	≥45 y				
Michelsen et al,[8] 2023	<40 y	37	<45 y	37.0 / 51.5	14.5	0.97	−0.002
	40-52 y	46	≥45 y				
	≥53 y	56	≥45 y				
Tuesley et al,[25] 2020	<35 y	32	<45 y	38.4 / 54.8	16.4	0.68	−0.023
	35-44 y	39.5	<45 y				
	45-54 y	49.5	≥45 y				
	55-64 y	59.5	≥45 y				
	≥65 y	68	≥45 y				

Abbreviations: HR, hazard ratio.

Each cohort contributes 1 row per age band as that band was published. The assigned midpoint is the value at which the band enters the continuous meta-regression shown in Figure 5, and the subgroup column gives the side of the 45-year cut-off to which the band was allocated in the analyses at that threshold.

Closed bands take their arithmetic midpoint. Open-ended bands take a midpoint 3 years beyond the reported cut-off, with 2 exceptions, both of which are bands that span the 45-year threshold or sit immediately above it: the ≥40-year band of Xing et al,[2] 2024 is assigned 46 years, and the >45-year band of Appiah et al,[5] 2015 is assigned 47.5 years. Bands spanning the cut-off are allocated to the older group by their midpoint, identically for both cohorts to which the rule applies (Michelsen et al,[8] 2023, 40-52 years, midpoint 46 years; Xing et al,[2] 2024, ≥40 years, midpoint 46 years), matching eTable 20, Figures 4A and 4B, and Figure 5. Assigned midpoints were varied by 3 years in either direction as a sensitivity analysis (eTable 20).

The mean age of each group is the precision-weighted mean of the midpoints of the bands it contains. The age interval is the difference between the 2 means and is the distance across which that cohort's contrast is measured. The ratio of HRs is the ≥45-year estimate divided by the <45-year estimate, each obtained by fixed-effect combination within the cohort; the per-year equivalent is its natural logarithm divided by the age interval.

The age interval varies just over threefold across cohorts, from 5.5 to 16.7 years, so a cohort with a narrow interval measures a smaller contrast for the same underlying gradient. That does not, however, explain the disagreement between cohorts: the unweighted correlation between the age interval and the log ratio of HRs is −0.09 for all-cause mortality and −0.10 for cardiovascular mortality, weighted regression of the contrast on the interval gives $P = .52$ and $P = .61$ respectively, and substantial heterogeneity remains after adjustment (residual $Q = 23.67$; $df = 5$; $P < .001$ for all-cause mortality; $Q = 5.72$; $df = 3$; $P = .13$ for cardiovascular mortality).

The per-year equivalents in this table are contrast-based and are not identical to the per-cohort slopes fitted by the continuous model in Figure 5, which use every band rather than 2 combined groups; the 2 agree in sign for all but 1 cohort on each outcome, and differ in magnitude by up to twofold. Michelsen et al,[8] 2023 is the cohort whose contrast-based value and fitted slope differ in sign, for both outcomes. The cohorts whose fitted slopes run counter to the pooled direction are therefore not identical to those with a ratio of HRs above 1.00 in this table, which are Appiah et al,[5] 2015 alone for all-cause mortality and none for cardiovascular mortality.

Consistent with the extraction rule set out in the eMethods, the minimally adjusted estimate was taken where a study reported more than 1 model. For Tuesley et al,[25] 2020 this gives Model 1 for all-cause mortality, for which that report publishes 2 models, and Model 2 for cardiovascular mortality, for which it publishes only 1. The rule bites on 1 further cohort: Appiah et al,[5] 2015 reports both an age-adjusted and a multivariable-adjusted estimate for each stratum, and the age-adjusted estimate was taken.

© 2026 Armeni E. *JAMA Network Open*. This is an open access article distributed under the terms of the CC-BY-NC-ND License, which does not permit alteration or commercial use, including those for text and data mining, AI training, and similar technologies.

eTable 20. Age at iatrogenic menopause as a moderator of mortality and cardiovascular outcomes, by analysis set and unit of inference

Analysis set	Cohorts, No.	Age bands, No.	Ratio of HRs, bands independent	*P* value	Ratio of HRs, cohort-clustered (95% CI)	*P* value
**All-cause mortality**						
Primary analysis: all contributing cohorts	7	25	0.84	.003	0.82 (0.68-0.98)	.04
Tier 1: restricted to no-surgery referent	5	18	0.84	.01	0.83 (0.63-1.09)	.13
Tier 2: Tier 1 excluding Appiah et al,[5] 2015	4	16	0.82	.009	0.78 (0.56-1.08)	.09
Tier 3: strict referent definition	3	13	0.84	.03	0.82 (0.54-1.25)	.18
Tier 1 plus Xu et al,[3] 2022	6	23	0.86	.01	0.85 (0.69-1.04)	.09
Tier 1 plus Xing et al,[2] 2024	6	20	0.82	0.003	0.80 (0.64-1.00)	.05
Tier 1, band spanning cut-off excluded	5	17	0.83	.01	0.84 (0.63-1.13)	.18
**Cardiovascular mortality**						
Primary analysis: all contributing cohorts	5	18	0.74	.004	0.77 (0.58-1.04)	.07
Tier 1: restricted to no-surgery referent	4	13	0.79	.04	0.82 (0.57-1.17)	.17
Tier 2: Tier 1 excluding Appiah et al,[5] 2015	3	11	0.73	.03	0.74 (0.44-1.26)	.13
Tier 3: strict referent definition	2	8	0.75	.10	Not estimable	—
Tier 1, band spanning cut-off excluded	4	12	0.78	.05	0.84 (0.54-1.29)	.29
**Cardiovascular events, coronary heart disease and stroke (continuous age, per 5-year increment)**						
Composite cardiovascular events	4	12	0.83	<.001	0.83 (0.70-1.00)	.04
Coronary heart disease	3	10	0.81	<.001	0.89 (0.61-1.30)	.31
Stroke	3	10	0.84	<.001	0.84 (0.68-1.03)	.06
**Mortality outcomes, continuous age, per 5-year increment (primary analysis set, Figure 5)**						
All-cause mortality	7	25	0.95 (0.92-0.98)	.002	0.94 (0.86-1.02)	.12
All-cause mortality, within-cohort component	7	25	0.94 (0.91-0.98)	.002	—	—
All-cause mortality, between-cohort component	7	25	0.99 (0.89-1.10)	.79	—	—
Cardiovascular mortality	5	18	0.91 (0.85-0.97)	.003	0.92 (0.79-1.08)	.22
Cardiovascular mortality, within-cohort component	5	18	0.91 (0.85-0.97)	.006	—	—
Cardiovascular mortality, between-cohort component	5	18	0.83 (0.63-1.09)	.18	—	—

Abbreviations: CI, confidence interval; HR, hazard ratio.
The ratio of hazard ratios is the pooled estimate for age bands at or above 45 years divided by the pooled estimate for bands below 45 years; a value below 1 indicates a smaller association with mortality when menopause occurred at an older age. The age bands contributed by each cohort and the age interval across which each contrast is measured are given in eTable 19.
The "bands independent" columns treat every age band as an independent observation and are anti-conservative, because several cohorts contribute more than one band; they are shown for comparison of the *P* values only and are not intended for interpretation, which is why no confidence intervals are given for the dichotomous models. The "cohort-clustered" columns are the reported analysis: bands below and at or above 45 years were combined by fixed effect within each cohort, the contrast was taken within the cohort, and cohort-level contrasts were pooled by random effects with the Knapp-Hartung adjustment.
The primary analysis set is the full stratum set plotted in Figure 5 and is the same set throughout this table: 25 all-cause estimates from 7 cohorts and 18 cardiovascular mortality estimates from 5 cohorts. Age bands spanning the 45-year cut-off are assigned to the older group by band midpoint (Michelsen et al,[8] 2023, 40-52 years, midpoint 46 years; Xing et al,[2] 2024, ≥40 years, midpoint 46 years), consistently in every analysis and in Figures 4A, 4B and 5. The single row labelled "band spanning cut-off excluded" is the sensitivity analysis under the alternative rule.

© 2026 Armeni E. *JAMA Network Open*. This is an open access article distributed under the terms of the CC-BY-NC-ND License, which does not permit alteration or commercial use, including those for text and data mining, AI training, and similar technologies.

The tier rows are the comparator-restriction sensitivity analysis. Tier 1 restricts the set to cohorts using a referent defined by absence of surgery, excluding the cohorts with a natural-menopause referent (Xing et al,[2] 2024 and Xu et al,[3] 2022). Tier 2 additionally excludes Appiah et al,[5] 2015, whose comparator group, defined as no bilateral salpingo-oophorectomy, includes women who underwent hysterectomy with ovarian conservation. Tier 3 retains only cohorts in which hysterectomy alone is a separate exposure group; the Olmsted County cohort (Rocca et al,[6] 2006; Rivera et al,[7] 2009) is excluded because its referent women were selected only on the absence of oophorectomy and hysterectomy was not an exclusion criterion, while 95% of its exposed women underwent oophorectomy with hysterectomy.

Cardiovascular mortality at Tier 3 rests on 2 cohorts and 1 residual degree of freedom; the resulting interval spans an 87-fold range and is reported as not estimable.

The within-cohort component for cardiovascular mortality coincides with the estimate from the single-covariate model to 2 decimal places because the between-cohort component for that outcome is close to null, so the gradient is expressed almost entirely within cohorts. The 2 rows are nonetheless distinct quantities, estimated from different models and carrying different standard errors, and the coincidence is a property of these data rather than a repeated entry.

Across the 10 estimable mortality specifications the ratio of hazard ratios ranged from 0.74 to 0.85 (median, 0.82; interquartile range, 0.79-0.84). The 3 event-outcome gradients are a different quantity and are excluded from that summary.

Between-cohort heterogeneity in the cohort-level contrast is substantial for all-cause mortality (Q = 26.5; df = 6; P < .001) and not for cardiovascular mortality (Q = 7.03; df = 4; P = .13).

The three event outcomes are reported as the ratio of hazard ratios per 5-year increment in age at iatrogenic menopause, not as a contrast across 45 years, because the contributing cohorts reported age bands divided at 48, 49 or 50 years and no band boundary at 45 years was available in more than 1 cohort.

Cohort-level gradients for the event outcomes were markedly heterogeneous (Q = 32.1; df = 3; P < .001 for composite cardiovascular events; Q = 30.6; df = 2; P < .001 for coronary heart disease; Q = 13.3; df = 2; P = .001 for stroke), 1 cohort (Poorthuis et al,[9] 2022) showing no gradient for composite cardiovascular events and a gradient in the opposite direction for coronary heart disease. A quadratic term was not supported for any outcome (all likelihood ratio P = .29 to .73).

The continuous all-cause model does not reach conventional significance in its primary form, and only 1 omission makes it do so: omission of Xing et al,[2] 2024, whose gradient of −0.044 per year is the steepest in the series and rests on 2 bands, one of them open above 40 years, gives 0.95 per 5 years (95% CI, 0.92-0.98; P = .01), because that cohort is also the principal source of the between-cohort disagreement in slope. No other single omission moves the P value below .05 (range, .07-.18), and the point estimate is stable at 0.92 to 0.97 per 5 years across every specification examined. Shifting all open-ended band midpoints together by ±3 years gives P = .09 to .15, so the result is robust to the midpoint rule and sensitive only to cohort composition.

The all-cause age strata of the Olmsted County cohort (Rocca et al,[6] 2006) are reported for prophylactic oophorectomy only, whereas its pooled estimate and its cardiovascular mortality age strata are for oophorectomy of any indication (eTable 18).

© 2026 Armeni E. *JAMA Network Open*. This is an open access article distributed under the terms of the CC-BY-NC-ND License, which does not permit alteration or commercial use, including those for text and data mining, AI training, and similar technologies.

eTable 21. Age at iatrogenic menopause as a continuous moderator, pooled across cardiovascular morbidity and mortality end points, with independent source populations as the unit of inference

Hazard ratios are expressed per 5-year increment in age at iatrogenic menopause. A value below 1.00 indicates that the association with cardiovascular risk is weaker when menopause occurs at an older age.

Analysis	Contributing data	HR per 5 y (95% CI)	P value	Detail
**Primary analysis**				
**Pooled across morbidity and mortality endpoints**	**11 populations, 37 age bands**	**0.89 (0.83-0.97)**	**.01**	**9 of 11 negative**
**Population-specific gradients**				
Farland 2023[11]	Composite CV events, 2 bands	0.71 (0.58-0.86)	—	47-53 y
Olmsted County[6,7]	All-cause mortality, 3 bands	0.77 (0.63-0.94)	—	42-53 y
Zhu 2020[15]	Composite CV events, 6 bands	0.78 (0.76-0.81)	—	32-58 y
Xing 2024[2]	All-cause mortality, 2 bands	0.80 (0.71-0.90)	—	37-46 y
Ingelsson 2010[1]	Composite CV events, 2 bands	0.87 (0.85-0.90)	—	46-53 y
Tuesley 2020[25]	All-cause mortality, 5 bands	0.94 (0.91-0.97)	—	32-68 y
Poorthuis 2022[9]	Composite CV events, 2 bands	0.94 (0.84-1.06)	—	45-51 y
Gierach 2014[10]	All-cause mortality, 5 bands	0.95 (0.93-0.98)	—	32-54 y
Xu 2022[3]	All-cause mortality, 5 bands	0.95 (0.88-1.03)	—	37-56 y
Appiah 2015[5]	All-cause mortality, 2 bands	1.03 (0.88-1.19)	—	42-47 y
Michelsen 2023[8]	All-cause mortality, 3 bands	1.04 (0.96-1.13)	—	37-56 y
**Endpoint-pairing sensitivity**				
Composite events + all-cause mortality (primary)	11 populations	0.89 (0.83-0.97)	.01	
Composite events + cardiovascular mortality	11 populations	0.88 (0.81-0.95)	.004	
Stroke + all-cause mortality	11 populations	0.89 (0.82-0.96)	.008	
Stroke + cardiovascular mortality	11 populations	0.87 (0.81-0.94)	.003	
Coronary heart disease + all-cause mortality	11 populations	0.90 (0.83-0.98)	.022	
Coronary heart disease + cardiovascular mortality	11 populations	0.88 (0.81-0.96)	.010	
**Leave-one-population-out**				
Omit Appiah 2015[5]	10 populations	0.88 (0.82-0.96)	.008	
Omit Farland 2023[11]	10 populations	0.91 (0.84-0.98)	.015	
Omit Gierach 2014[10]	10 populations	0.89 (0.81-0.97)	.013	
Omit Ingelsson 2010[1]	10 populations	0.90 (0.82-0.98)	.021	
Omit Michelsen 2023[8]	10 populations	0.88 (0.81-0.95)	.005	
Omit Olmsted County[6,7]	10 populations	0.90 (0.83-0.98)	.019	
Omit Poorthuis 2022[9]	10 populations	0.89 (0.81-0.97)	.014	
Omit Tuesley 2020[25]	10 populations	0.89 (0.81-0.97)	.014	

© 2026 Armeni E. *JAMA Network Open*. This is an open access article distributed under the terms of the CC-BY-NC-ND License, which does not permit alteration or commercial use, including those for text and data mining, AI training, and similar technologies.

Analysis	Contributing data	HR per 5 y (95% CI)	P value	Detail
Omit Xing 2024[2]	10 populations	0.90 (0.83-0.98)	.023	
Omit Xu 2022[3]	10 populations	0.89 (0.81-0.97)	.013	
Omit Zhu 2020[15]	10 populations	0.91 (0.85-0.99)	.026	
**Distribution-free analyses**				
Combined Kendall tau, populations with ≥3 bands	6 populations	tau = -0.78	<.001	5 of 6 monotone decreasing
Exact sign test on population slopes	11 populations	9/11 negative	.065	Two-sided
Exact sign-flip permutation	2048 permutations	—	.007	Smallest attainable P = 0.0005
**Outcome-specific gradients, for comparison**				
All-cause mortality	7 populations	0.94 (0.86-1.02)	.124	
Cardiovascular mortality	5 populations	0.92 (0.79-1.08)	.214	
Composite cardiovascular events	4 populations	0.83 (0.70-1.00)	.048	
Coronary heart disease	3 populations	0.89 (0.61-1.30)	.308	
Stroke	3 populations	0.84 (0.68-1.03)	.066	

One endpoint was taken per source population, so that no population contributes twice. No population reports age bands for both a morbidity and a mortality endpoint, so the 4 event-reporting and 7 mortality-reporting populations are mutually exclusive. In the endpoint-pairing sensitivity analyses, each event-reporting population contributes the named event endpoint where age-band estimates for that endpoint are available; Farland et al,[11] 2023 reports age-band estimates for composite cardiovascular events only and contributes that endpoint in every pairing, which is why each pairing retains 11 populations. Rocca et al,[6] 2006 and Rivera et al,[7] 2009 are separate reports of the Olmsted County population and are combined into a single cluster.

Within each population the slope of the log hazard ratio on the assigned age-band midpoint was fitted by weighted least squares with fixed dispersion; the population-specific slopes were then pooled by random-effects meta-analysis with restricted maximum-likelihood estimation of the between-population variance and the Knapp-Hartung adjustment.

Between-population heterogeneity in the fitted gradient was substantial (Q = 137.9; df = 10; P < .001): populations agreed in the direction of the gradient but not in its magnitude, so these data support the existence of a continuous decline in risk with older age at surgery without resolving its slope.

The exact sign-flip permutation test enumerates all 2^{11} = 2048 sign assignments of the population 11 population slopes; the reported P value is two-sided, and the smallest attainable two-sided P value is .001. The combined Kendall rank correlation is precision-weighted across the 6 populations reporting 3 or more age bands.

This analysis was post hoc and assumes that the proportional attenuation of risk with older age at surgery is common to fatal and non-fatal endpoints. It is reported as exploratory.

Abbreviations: CI, confidence interval; CV, cardiovascular; HR, hazard ratio; y, years.

© 2026 Armeni E. *JAMA Network Open*. This is an open access article distributed under the terms of the CC-BY-NC-ND License, which does not permit alteration or commercial use, including those for text and data mining, AI training, and similar technologies.

eTable 22. Within-cohort modification of the association by menopausal hormone therapy stratum

Source	Comparison	Estimate, First Stratum	Estimate, Second Stratum	Test of Modification	*P* Value
Rocca et al,[6] 2006	All-cause mortality after iatrogenic menopause before age 45 y, against a common referent	Hormone therapy users, 1.27 (0.67-2.39)	Non-users, 1.80 (1.25-2.59)	Ratio of HRs, non-users to users, 1.31 (0.69-2.50)	.41
Xing et al,[2] 2024	Gradient in all-cause mortality with age at surgery, within surgical menopause, per year	Hormone therapy users, slope −0.0208	Non-users, slope −0.0167	Interaction, −0.0040 per year (SE, 0.0211)	.85
Xu et al,[3] 2022	Hormone therapy compared with no hormone therapy, within menopause type	Surgical menopause, 0.74 (0.58-0.93)	Natural menopause, 0.87 (0.81-0.93)	Ratio, surgical to natural, 0.85 (0.67-1.09)	.19

Estimates are reproduced from the subgroup data in eTable 13 and are not pooled, because only 3 cohorts report both hormone therapy strata for all-cause mortality. eTable 13 contains hormone therapy-stratified data for further cohorts, but these report cardiovascular or coronary events rather than all-cause mortality; they include a directly reported 2-stratum contrast in Olesen et al,[14] 2022 (non-users, 1.38 [95% CI, 0.80-2.38]; users, 1.28 [95% CI, 0.80-2.05]) and estimates across 5 age bands in Zhu et al,[15] 2020.

For Rocca et al,[6] 2006 the estimate shown for hormone therapy users is the adjusted hazard ratio reported by the authors, whereas the estimate shown for non-users is a crude risk ratio derived from the reported counts (23 of 79 deaths) against the same referent (229 of 1,417). The test of modification is calculated from the crude estimates in both strata, that is, 1.80 divided by the crude risk ratio of 1.38 for the hormone therapy stratum (10 of 45 against the same referent), and therefore does not equal the ratio of the 2 estimates displayed.

Because both Rocca strata are compared with the same referent group, the variance contributed by that group cancels from the contrast, and the interval shown is calculated accordingly (SE, 0.330 on the log scale). Treating the 2 strata as independent would give 1.31 (95% CI, 0.67-2.55; *P* = .43) and would overstate the variance.

The contrasts reported for Rocca et al,[6] 2006 and Xu et al,[3] 2022 estimate the same interaction parameter from opposite directions, since the 2 × 2 interaction is symmetric. Expressed on a common scale as the effect among hormone therapy users divided by the effect among non-users, they are 0.76 (95% CI, 0.40-1.46) and 0.85 (95% CI, 0.67-1.09), respectively, both indicating attenuation and neither significant. The contrast reported for Xing et al,2 2024 estimates a different parameter, the interaction with the age gradient.

The slopes shown for Xing et al,[2] 2024 are rounded to 4 decimal places and their displayed difference is −0.0041; the interaction term shown is calculated from the unrounded slopes. The *P* value is unaffected (*P* = .85 on either value).

For Xing et al,[2] 2024, eTable 13 does not state whether the 2 series are referenced to a common stratum or each to its own stratum at ages 45 to 54 years. The interaction test is unaffected, since it compares slopes, but the absolute levels should be interpreted with this ambiguity in mind.

Abildgaard et al,[16] 2018 also reported estimates by hormone therapy stratum but is not included, because the same cell is labeled as an age stratum in eTable 8 and as a non-hormone-therapy subgroup in eTable 13, and the conflict could not be resolved from the published report. That cell contains a single death, and its interval (0.07-4.22) is correspondingly uninformative.

Abbreviations: CI, confidence interval; HR, hazard ratio; SE, standard error; y, years.

© 2026 Armeni E. *JAMA Network Open*. This is an open access article distributed under the terms of the CC-BY-NC-ND License, which does not permit alteration or commercial use, including those for text and data mining, AI training, and similar technologies.

eTable 23. Exploratory cohort-level meta-regression of all-cause mortality on menopausal hormone therapy prevalence

Moderator	Cohorts	Ratio of HRs per 10 Percentage Points	95% CI (Wald)	Wald *P* Value	95% CI (Knapp-Hartung)	Knapp-Hartung *P* Value	R^2, %
Difference in prevalence, exposed arm minus comparator arm	6	1.01	0.94-1.08	.82	0.91-1.11	.83	0
Prevalence, exposed arm	6	0.98	0.95-1.01	.13	0.93-1.02	.25	50
Sensitivity analysis: difference in prevalence, restricted to cohorts with a no-oophorectomy or no-pelvic-surgery referent	5	1.01	0.93-1.10	.78	0.88-1.16	.80	—

All models are random-effects meta-regressions of the log hazard ratio for all-cause mortality on a single cohort-level covariate, with the between-study variance estimated by restricted maximum likelihood and inference by the Knapp-Hartung adjustment. Residual degrees of freedom = 4 for the 2 primary models and 3 for the sensitivity model. A ratio of hazard ratios below 1.00 indicates that cohorts with higher hormone therapy prevalence reported lower hazard ratios for the association between iatrogenic menopause and all-cause mortality. Both primary analyses were prespecified as exploratory.

The 6 contributing cohorts are Appiah et al,[5] 2015; Appiah et al,[4] 2020; Gierach et al,[10] 2014; Michelsen et al,[8] 2023; Wilson et al,[13] 2019; and Xing et al,[2] 2024. The sensitivity model excludes Xing et al,[2] 2024, the only 1 of the 6 that uses a natural-menopause referent.

The between-study variance fell from 0.0049 to 0.0025 for the exposed-arm model and rose from 0.0049 to 0.0065 for the difference model, so no heterogeneity is explained by the latter. R^2 estimated from 6 cohorts and a single covariate is highly unstable and values of this magnitude arise frequently by chance at this sample size; the R^2 column should not be read as a measure of heterogeneity genuinely explained. R^2 was not computed for the sensitivity model.

In leave-one-out analysis of the exposed-arm model the Knapp-Hartung P value ranged from .12 to .93 and did not fall below .05 on omission of any single cohort. An exact permutation test over all 720 permutations of the moderator gave P = .23 for the exposed-arm model and P = .81 for the difference model.

The 2 moderators correlate at r = 0.66, which on 6 cohorts carries a 95% CI of approximately −0.32 to 0.96. They are different covariates estimating different quantities — the absolute prevalence in the exposed arm and the difference in prevalence between arms — and the fitted slopes differ accordingly (+0.00079 and −0.00225 per percentage point). Neither is distinguishable from 0 in this set of cohorts.

Hormone therapy prevalence by exposure group was not reported for 3 of the 9 cohorts contributing to all-cause mortality (Rocca et al,[6] 2006; Tuesley et al,[25] 2020; and Xu et al,[3] 2022), which do not contribute to these models. For cardiovascular mortality it was not reported for 4 of the 7 contributing estimates — which arise from 6 cohorts, since Rivera et al,[7] 2009 and Rocca et al,[6] 2006 report the same Olmsted County population (eTable 17) — leaving 3 cohorts and 1 residual degree of freedom, so no model was fitted.

Cohorts reporting prevalence had higher pooled hazard ratios than the full sets for both outcomes, so these models are fitted on a non-random subset of the evidence. For all-cause mortality, the outcome modeled here, the difference is small (pooled HR, 1.09 across the 6 reporting cohorts vs 1.08 across all 9). It is larger for cardiovascular mortality, where no model was fitted (1.09 across the 3 reporting cohorts vs 1.03 across all 7 estimates).

Prevalence values are taken from eTable 12. Where that table reports never-use, ever-use was derived as 100 minus never-use (Appiah et al,[5] 2015 and Gierach et al,[10] 2014). For Xing et al,[2] 2024, the only cohort reporting prevalence by age band, a weighted mean was computed after back-solving band denominators from the reported percentages and counts; the resulting denominators (2,206 exposed and 6,159 comparator women) are smaller than the numbers contributing to the pooled analysis (2,410 and 7,937), so prevalence for this cohort is computed on a subset of each arm.

Abbreviations: CI, confidence interval; HR, hazard ratio.

© 2026 Armeni E. *JAMA Network Open*. This is an open access article distributed under the terms of the CC-BY-NC-ND License, which does not permit alteration or commercial use, including those for text and data mining, AI training, and similar technologies.

eFigures 1 to 5. Pooled estimates stratified by comparator type

Each panel repeats a primary pooled analysis with the contributing cohorts grouped by the referent population used for comparison. These analyses were not prespecified and are reported as post hoc and exploratory. Comparator subgroups are presented because the referent definitions do not estimate the same quantity, and because in at least 1 outcome comparator type is confounded with age at iatrogenic menopause (eFigure 2). All subgroup and overall estimates were obtained from random-effects models (restricted maximum-likelihood τ^2; Wald 95% CIs), with subgroup-specific τ^2 unless stated otherwise; single-cohort subgroups are shown with their own 95% CI and no between-study heterogeneity statistic. Tests for subgroup differences are reported as χ^2 with the corresponding df and *P* value, and the accompanying I^2 is the proportion of the variation attributable to differences between subgroups, not the within-subgroup heterogeneity. The test for subgroup differences reached conventional significance for coronary heart disease only (eFigure 2). In 4 of the 5 outcomes at least 1 referent group is represented by a single cohort, and in the composite cardiovascular events analysis 2 of the 3 groups are; all-cause mortality is the only outcome in which both referent groups contain more than 1 cohort. No adjustment was made for testing across 5 outcomes.

© 2026 Armeni E. *JAMA Network Open*. This is an open access article distributed under the terms of the CC-BY-NC-ND License, which does not permit alteration or commercial use, including those for text and data mining, AI training, and similar technologies.

eFigure 1. Composite cardiovascular events, stratified by comparator type.

eFigure 1. Composite cardiovascular events, stratified by comparator type

Study or subgroup	HR (95% CI)	Iatrogenic menopause, No.	Comparators, No.
Natural menopause as reference group (1 cohort)			
Zhu et al,[15] 2020	1.22 (1.16-1.28)	25,463	178,304
**Subtotal**	**1.22 (1.16-1.28)**	**25,463**	**178,304**
No oophorectomy as reference group (5 cohorts)			
do Valle et al,[19] 2021	1.79 (1.00-3.22)	360	3,600
Farland et al,[11] 2023	1.58 (1.40-1.79)	2,153	108,448
Howard et al,[22] 2005	1.28 (1.16-1.42)	18,251	52,976
Ingelsson et al,[1] 2010	1.16 (1.03-1.30)	5,118	643,925
Poorthuis et al,[9] 2022	1.18 (1.11-1.25)	1,360	272,884
**Subtotal (random effects)**	**1.30 (1.14-1.49)**	**27,242**	**1,081,833**
Menopause after cancer treatment as reference group (1 cohort)			
Krul et al,[23] 2018	0.89 (0.65-1.21)	299	619
**Subtotal**	**0.89 (0.65-1.21)**	**299**	**619**
**Overall (random effects)**	**1.25 (1.12-1.39)**	**53,004**	**1,260,756**

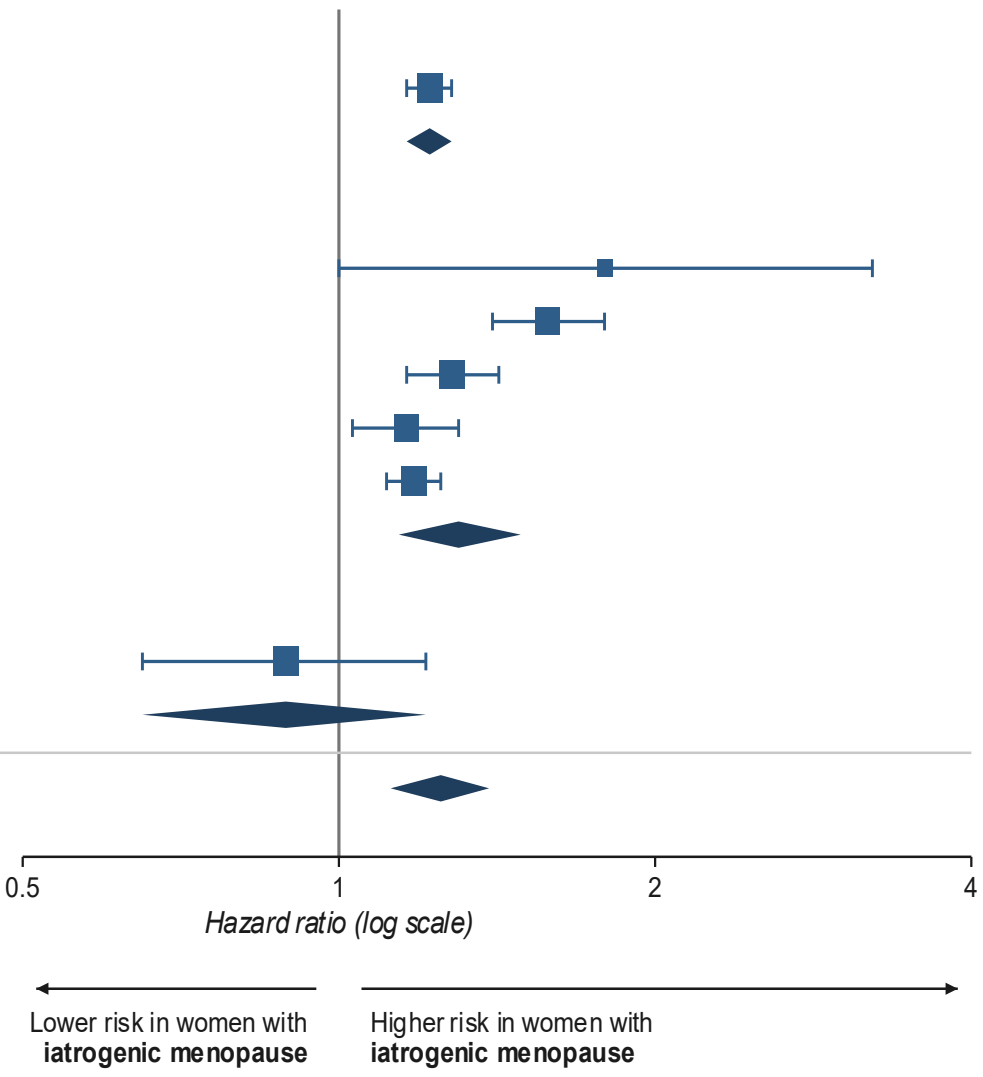

Each estimate compares women with iatrogenic menopause against the reference group named in its subgroup heading.

Test for subgroup differences: $\chi^2 = 5.00$; df = 2; P = .08

Forest plot of the composite cardiovascular events outcome (7 cohorts), with studies grouped by the referent population used for comparison: natural menopause (a single cohort, Zhu et al,[15] 2020); no oophorectomy or no pelvic surgery (5 cohorts: Poorthuis et al,[9] 2022; Howard et al,[22] 2005; do Valle et al,[19] 2021; Farland et al,[11] 2023; and Ingelsson et al,[1] 2010); and menopause after cancer treatment without premature ovarian insufficiency (a single cohort, Krul et al,[23] 2018). The test for subgroup differences ($\chi^2 = 5.00$; df = 2; $P = .08$) does not reach conventional significance; 2 of the 3 subgroups contain a single cohort, and under a common between-study variance the same comparison gives $\chi^2 = 2.96$ (df = 2; $P = .23$). Comparator type does not explain the heterogeneity present in the overall analysis ($I^2 = 86\%$), which is concentrated in a single cohort: omitting Farland et al,[11] 2023 alone reduces τ^2 from 0.02 to 0 and leaves a residual Q of 7.99 (df = 5; $P = .16$), so that $I^2 = 0\%$ (eTable 16).

© 2026 Armeni E. *JAMA Network Open*. This is an open access article distributed under the terms of the CC-BY-NC-ND License, which does not permit alteration or commercial use, including those for text and data mining, AI training, and similar technologies.

eFigure 2. Coronary heart disease, stratified by comparator type.

eFigure 2. Coronary heart disease, stratified by comparator type

Study or subgroup	HR (95% CI)	Iatrogenic menopause, No.	Comparators, No.
Natural menopause as reference group (2 cohorts)			
Dam et al,[18] 2019	1.22 (1.01-1.47)	856	9,152
Zhu et al,[15] 2020	1.26 (1.19-1.33)	25,463	178,304
**Subtotal (random effects)**	**1.26 (1.19-1.33)**	**26,319**	**187,456**
No oophorectomy as reference group (3 cohorts)			
Farland et al,[11] 2023	1.70 (1.44-2.01)	2,153	108,448
Ingelsson et al,[1] 2010	1.12 (0.93-1.35)	5,118	643,925
Poorthuis et al,[9] 2022	1.17 (1.07-1.27)	1,360	272,884
**Subtotal (random effects)**	**1.30 (1.01-1.68)**	**8,631**	**1,025,257**
Menopause at ≥40 years as reference group (1 cohort)			
Honigberg et al,[12] 2019 †	3.76 (2.42-5.85)	644	138,712
**Subtotal**	**3.76 (2.42-5.85)**	**644**	**138,712**
**Overall (random effects)**	**1.48 (1.07-2.03)**	**35,594**	**1,351,425**

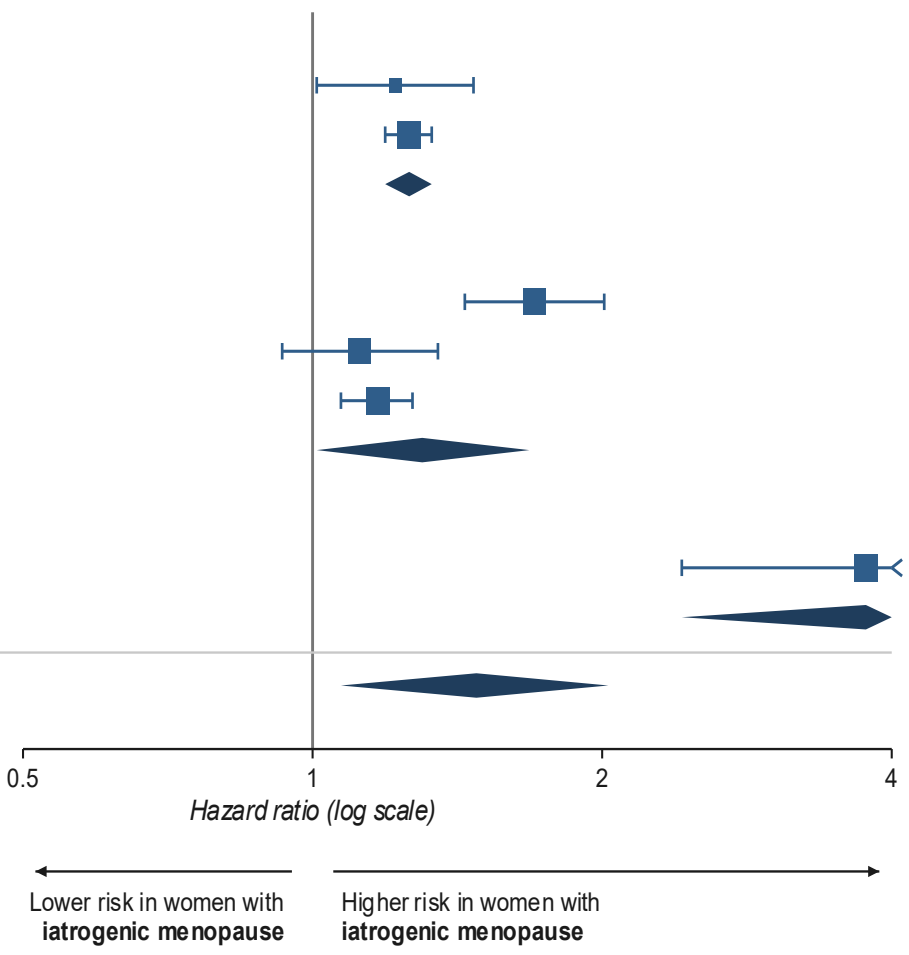

Each estimate compares women with iatrogenic menopause against the reference group named in its subgroup heading.

Test for subgroup differences: $\chi^2 = 23.28$; df = 2; P < .001

† The only cohort in which the exposure was itself restricted to menopause before age 40 years, so comparator type is confounded with age at menopause here.

Forest plot of the coronary heart disease outcome (6 cohorts), with studies grouped by referent population: natural menopause (2 cohorts: Zhu et al,[15] 2020 and Dam et al,[18] 2019); no oophorectomy (3 cohorts: Poorthuis et al,[9] 2022; Farland et al,[11] 2023; and Ingelsson et al,[1] 2010); and women without premature menopause, that is, menopause at age 40 years or older (a single cohort, Honigberg et al,[12] 2019, the only cohort in which the exposure was itself restricted to menopause before age 40 years). Test for subgroup differences: $\chi^2 = 23.28$; df = 2; $P < .001$; $I^2 = 91\%$. Unlike the composite cardiovascular events outcome, in which no such pattern was apparent, comparator subgroup here is confounded with age at menopause: the age-restricted cohort forms its own subgroup and drives the subgroup difference, which is absent when that cohort is omitted ($\chi^2 = 0.07$; df = 1; $P = .79$). It is not, however, the dominant source of heterogeneity, which persists on its omission (I^2 falling from 97% to 85%, with residual $Q = 16.83$; df = 4; $P = .002$; eTable 16).

© 2026 Armeni E. *JAMA Network Open*. This is an open access article distributed under the terms of the CC-BY-NC-ND License, which does not permit alteration or commercial use, including those for text and data mining, AI training, and similar technologies.

eFigure 3. Stroke, stratified by comparator type.

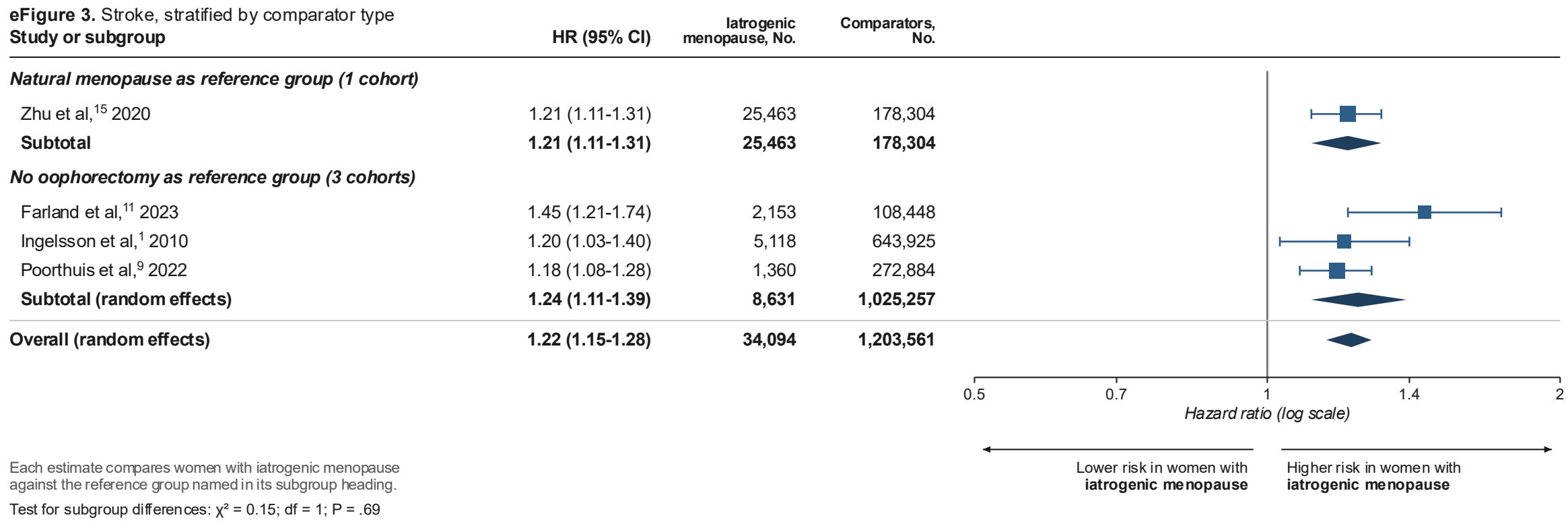

Forest plot of the stroke outcome (4 cohorts), with studies grouped by referent population: natural menopause (a single cohort, Zhu et al,[15] 2020) and no oophorectomy (3 cohorts: Poorthuis et al,[9] 2022; Farland et al,[11] 2023; and Ingelsson et al,[1] 2010). Test for subgroup differences: χ^2 = 0.15; df = 1; *P* = .69; I^2 = 0%. The test does not identify comparator type as a source of variation in this outcome, in which the restricted maximum-likelihood estimate of τ^2 lies at the boundary of 0, so that I^2 = 0% and the fixed-effect and random-effects estimates coincide; the classical (Q − df)/Q value calculated from the same Q of 4.02 (df = 3; *P* = .26) is 25% (eTable 9). With 4 cohorts divided 1 to 3, the comparison has little power to detect a difference between referent definitions.

© 2026 Armeni E. *JAMA Network Open*. This is an open access article distributed under the terms of the CC-BY-NC-ND License, which does not permit alteration or commercial use, including those for text and data mining, AI training, and similar technologies.

eFigure 4. Cardiovascular mortality, stratified by comparator type.

eFigure 4. Cardiovascular mortality, stratified by comparator type

Study or subgroup	HR (95% CI)	Iatrogenic menopause, No.	Comparators, No.
No surgery as reference group (6 cohorts)			
Appiah et al,[5] 2015, nondiabetes	1.11 (0.97-1.27)	1,340	5,009
Appiah et al,[4] 2020	1.02 (0.82-1.26)	610	2,158
Michelsen et al,[8] 2023	1.15 (0.95-1.40)	907	18,673
Rivera et al,[7] 2009 †	0.87 (0.69-1.10)	1,091	2,383
Rocca et al,[6] 2006 †	0.75 (0.52-1.09)	1,091	2,383
Tuesley et al,[25] 2020	0.96 (0.84-1.10)	18,558	553,958
**Subtotal (random effects)**	**1.01 (0.92-1.11)**	**23,597**	**584,564**
Natural menopause as reference group (1 cohort)			
Xu et al,[3] 2022	1.22 (1.00-1.50)	17,569	50,345
**Subtotal**	**1.22 (1.00-1.50)**	**17,569**	**50,345**
**Overall (random effects)**	**1.03 (0.94-1.13)**	**41,166**	**634,909**

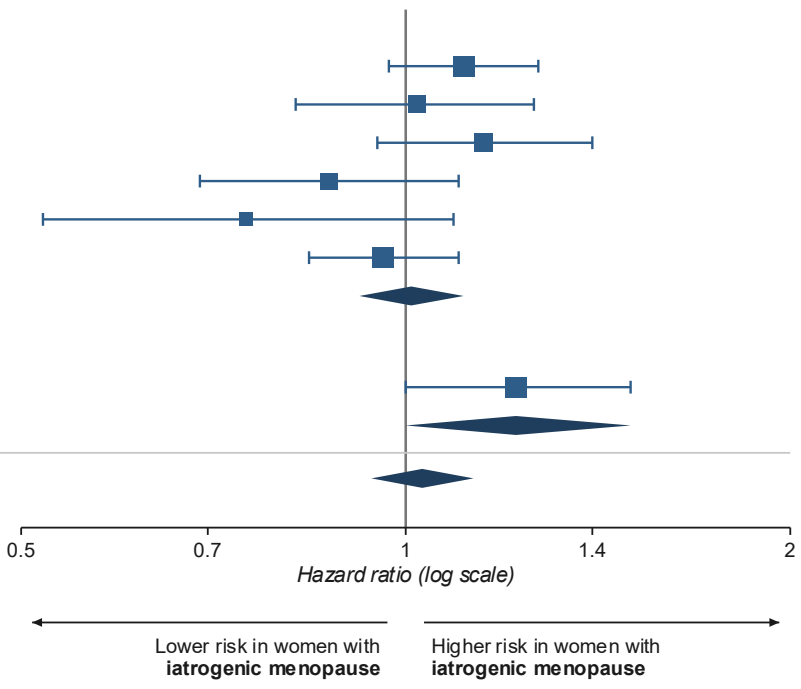

Each estimate compares women with iatrogenic menopause against the reference group named in its subgroup heading.

Test for subgroup differences: $\chi^2 = 2.88$; df = 1; P = .09

† Separate reports of the same Olmsted County population; that cohort is counted twice within this subgroup and carries double weight.

Forest plot of cardiovascular mortality (7 estimates from 6 cohorts), with studies grouped by referent population: natural menopause (a single cohort, Xu et al,[3] 2022) and no surgery (6 estimates from 5 cohorts: Rivera et al,[7] 2009; Michelsen et al,[8] 2023; Tuesley et al,[25] 2020; Appiah et al,[4] 2020; Rocca et al,[6] 2006; and Appiah et al,[5] 2015). Test for subgroup differences: χ^2 = 2.88; df = 1; *P* = .09; I^2 = 65%. Neither subgroup estimate excluded the null, although the single-cohort natural-menopause subgroup lies on the threshold (1.22; 95% CI, 1.00-1.50; lower limit 0.998 before rounding). The no-surgery subgroup contains both reports of the Olmsted County cohort (Rivera et al,[7] 2009 and Rocca et al,[6] 2006), so that population is counted twice within it and carries double weight in the subgroup estimate and in the test for subgroup differences; counting it once moves the overall estimate away from the null, from 1.03 (95% CI, 0.94-1.13) to 1.08 (95% CI, 0.98-1.18) (eTable 17).

© 2026 Armeni E. *JAMA Network Open*. This is an open access article distributed under the terms of the CC-BY-NC-ND License, which does not permit alteration or commercial use, including those for text and data mining, AI training, and similar technologies.

eFigure 5. All-cause mortality, stratified by comparator type.

eFigure 5. All-cause mortality, stratified by comparator type

Study or subgroup	HR (95% CI)	Iatrogenic menopause, No.	Comparators, No.
Natural menopause as reference group (2 cohorts)			
Xing et al,[2] 2024	1.11 (1.00-1.23)	2,410	7,937
Xu et al,[3] 2022	1.22 (1.11-1.33)	17,569	50,345
**Subtotal (random effects)**	**1.16 (1.06-1.27)**	**19,979**	**58,282**
No surgery as reference group (7 cohorts)			
Appiah et al,[5] 2015, nondiabetes	1.10 (1.02-1.19)	1,340	5,009
Appiah et al,[4] 2020	1.05 (0.95-1.16)	610	2,158
Gierach et al,[10] 2014	0.99 (0.95-1.04)	12,652	28,947
Michelsen et al,[8] 2023	1.24 (1.09-1.42)	907	18,673
Rocca et al,[6] 2006	1.05 (0.92-1.20)	1,091	2,383
Tuesley et al,[25] 2020	0.94 (0.88-1.00)	18,558	553,958
Wilson et al,[13] 2019	1.30 (1.02-1.66)	851	10,218
**Subtotal (random effects)**	**1.06 (0.99-1.14)**	**36,009**	**621,346**
**Overall (random effects)**	**1.08 (1.01-1.16)**	**55,988**	**679,628**

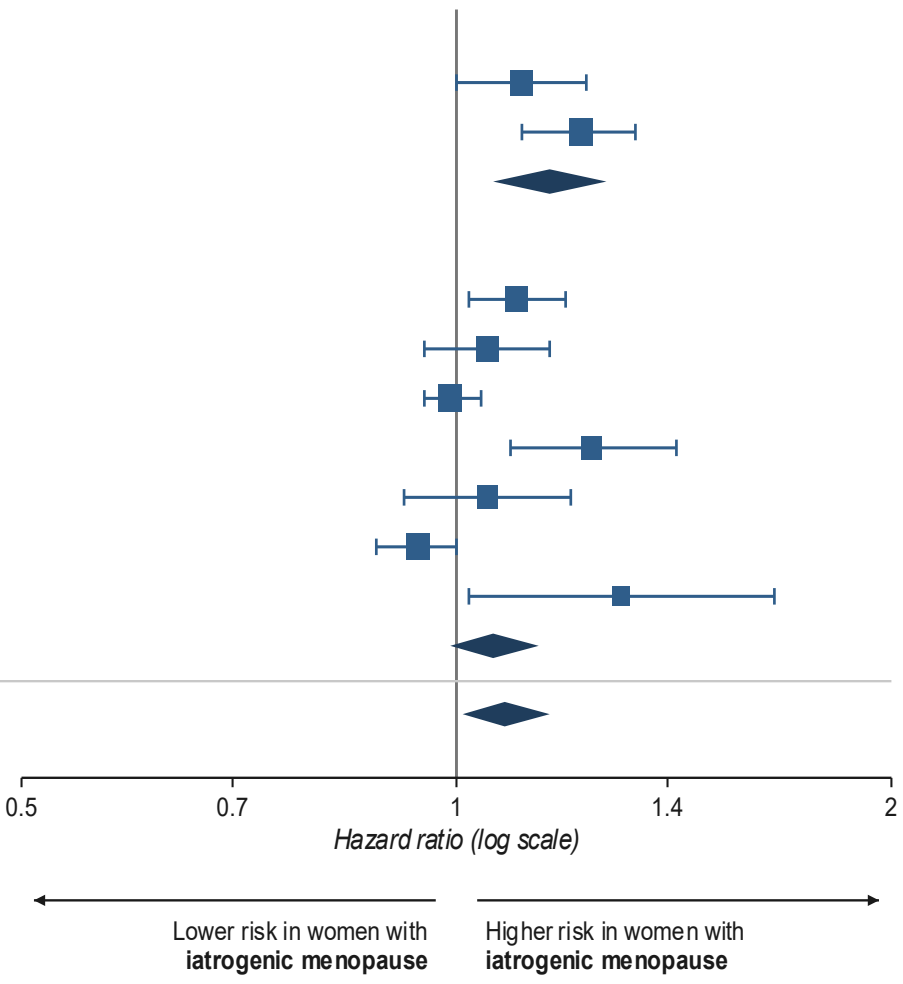

Each estimate compares women with iatrogenic menopause against the reference group named in its subgroup heading.

Test for subgroup differences: χ^2 = 2.30; df = 1; P = .13

Forest plot of all-cause mortality (9 cohorts), with studies grouped by referent population: natural menopause (2 cohorts: Xu et al,[3] 2022 and Xing et al,[2] 2024; 1.16; 95% CI, 1.06-1.27) and no surgery (7 cohorts: Michelsen et al,[8] 2023; Tuesley et al,[25] 2020; Wilson et al,[13] 2019; Gierach et al,[10] 2014; Appiah et al,[4] 2020; Rocca et al,[6] 2006; and Appiah et al,[5] 2015; 1.06; 95% CI, 0.99-1.14). Test for subgroup differences: χ^2 = 2.30; df = 1; *P* = .13; I^2 = 56%. This is the only outcome in which both referent groups contain more than 1 cohort. Comparator type is not established as a source of the overall heterogeneity (I^2 = 79%), and the pooled estimate is sensitive to omission of individual cohorts using either referent definition: it falls to the significance threshold on omission of Michelsen et al,[8] 2023 (1.07; 95% CI, 1.00-1.14; *P* = .05), which uses a no-oophorectomy referent, and loses conventional significance on omission of Xu et al,[3] 2022 (1.07; *P* = .06), which uses a natural-menopause referent, but not on omission of Xing et al,[2] 2024 (1.08; 95% CI, 1.01-1.17; *P* = .04) (eTable 16). Unlike cardiovascular mortality, this outcome contains no duplicated source population: Rivera et al,[7] 2009 contributed cardiovascular mortality only, so the Olmsted County cohort enters through Rocca et al,[6] 2006 alone.

© 2026 Armeni E. *JAMA Network Open*. This is an open access article distributed under the terms of the CC-BY-NC-ND License, which does not permit alteration or commercial use, including those for text and data mining, AI training, and similar technologies.

eReferences: References cited in this supplement are numbered independently of the main article

1. Ingelsson E, Lundholm C, Johansson ALV, Altman D. Hysterectomy and risk of cardiovascular disease: a population-based cohort study. European Heart Journal. 2010;32(6):745-750. doi:10.1093/eurheartj/ehq477
2. Xing Z, Kirby RS. Age at natural or surgical menopause, all-cause mortality, and lifespan among postmenopausal women in the United States. Menopause. 2024;31:176-185. doi:10.1097/GME.0000000000002314
3. Xu Z, Chung HF, Dobson AJ, Wilson LF, Hickey M, Mishra GD. Menopause, hysterectomy, menopausal hormone therapy and cause-specific mortality: cohort study of UK Biobank participants. Human Reproduction. 2022;37:2175-2185. doi:10.1093/humrep/deac137
4. Appiah D, Nwabuo CC, Owoade DR, Samad J, Ebong I, Winters SJ. Family history of premature myocardial infarction modifies the associations between bilateral oophorectomy and cardiovascular disease mortality in a US national cohort of postmenopausal women. Menopause. 2020;27(6):658-667. doi:10.1097/gme.0000000000001522
5. Appiah D, Winters SJ, Muldoon SB, Hornung CA, Cauley JA. Androgens, Bilateral Oophorectomy, and Cardiovascular Disease Mortality in Postmenopausal Women With and Without Diabetes: The Study of Osteoporotic Fractures. Diabetes Care. 2015;38(12):2301-2307. doi:10.2337/dc15-1434
6. Rocca WA, Grossardt BR, de Andrade M, Malkasian GD, Melton LJ, 3rd. Survival patterns after oophorectomy in premenopausal women: a population-based cohort study. Lancet Oncol. Oct 2006;7(10):821-8. doi:10.1016/s1470-2045(06)70869-5
7. Rivera CM, Grossardt BR, Rhodes DJ, Brown RD, Roger VL, Melton LJ. Increased cardiovascular mortality after early bilateral oophorectomy. Menopause. 2009;16:15-22. doi:10.1097/gme.0b013e31818888f7
8. Michelsen TM, Rosland TE, Åsvold BO, Pripp AH, Liavaag AH, Johansen N. All-cause and cardiovascular mortality after hysterectomy and oophorectomy in a large cohort (HUNT2). Acta Obstet Gynecol Scand. Apr 2023;102(4):465-472. doi:10.1111/aogs.14531
9. Poorthuis MHF, Yao P, Chen Y, et al. Risks of Stroke and Heart Disease Following Hysterectomy and Oophorectomy in Chinese Premenopausal Women. Stroke. Oct 2022;53(10):3064-3071. doi:10.1161/strokeaha.121.037305
10. Gierach GL, Pfeiffer RM, Patel DA, et al. Long-term overall and disease-specific mortality associated with benign gynecologic surgery performed at different ages. Menopause. Jun 2014;21(6):592-601. doi:10.1097/gme.0000000000000118
11. Farland LV, Rice MS, Degnan WJ, Rexrode KM, Manson JE, Rimm EB. Hysterectomy With and Without Oophorectomy, Tubal Ligation, and Risk of Cardiovascular Disease in the Nurses' Health Study II. Journal of Women's Health. 2023;doi:10.1089/jwh.2022.0207
12. Honigberg MC, Zekavat SM, Aragam K, et al. Association of Premature Natural and Surgical Menopause With Incident Cardiovascular Disease. Jama. Dec 24 2019;322(24):2411-2421. doi:10.1001/jama.2019.19191
13. Wilson LF, Pandeya N, Byles J, Mishra GD. Hysterectomy status and all-cause mortality in a 21-year Australian population-based cohort study. Am J Obstet Gynecol. Jan 2019;220(1):83.e1-83.e11. doi:10.1016/j.ajog.2018.10.002
14. Olesen CS, Koch T, Uldbjerg CS, Gregersen LS, Christensen J, Dehlendorff C. Cardiovascular mortality after bilateral oophorectomy: a prospective cohort study. Menopause. 2022;29:28-34. doi:10.1097/GME.0000000000001873
15. Zhu D, Chung HF, Dobson AJ, et al. Type of menopause, age of menopause and variations in the risk of incident cardiovascular disease: pooled analysis of individual data from 10 international studies. Hum Reprod. Aug 1 2020;35(8):1933-1943. doi:10.1093/humrep/deaa124
16. Abildgaard J, Ahlstrom MG, Daugaard G, Nielsen DL, Pedersen AT, Lindegaard B. Mortality and Risk of Cancer After Prophylactic Bilateral Oophorectomy in Women With a Family History of Cancer. JNCI Cancer Spectrum. 2018;2doi:10.1093/jncics/pky034
17. Allison MA, Manson JE, Langer RD, Carr JJ, Rossouw JE, Pettinger MB. Oophorectomy, hormone therapy, and subclinical coronary artery disease in women with hysterectomy. Menopause. 2008;15:639-647. doi:10.1097/gme.0b013e31816d5b1c

© 2026 Armeni E. *JAMA Network Open*. This is an open access article distributed under the terms of the CC-BY-NC-ND License, which does not permit alteration or commercial use, including those for text and data mining, AI training, and similar technologies.

18. Dam V, van der Schouw YT, Onland-Moret NC, et al. Association of menopausal characteristics and risk of coronary heart disease: a pan-European case-cohort analysis. Int J Epidemiol. Aug 1 2019;48(4):1275-1285. doi:10.1093/ije/dyz016

19. do Valle HA, Kaur P, Kwon JS, Cheifetz R, Dawson L, Hanley GE. Risk of cardiovascular disease among women carrying BRCA mutations after risk-reducing bilateral salpingo-oophorectomy: A population-based study. Gynecol Oncol. Sep 2021;162(3):707-714. doi:10.1016/j.ygyno.2021.06.022

20. Duan L, Xu X, Koebnick C, Lacey JV, Sullivan-Halley J, Templeman C. Bilateral oophorectomy is not associated with increased mortality: the California Teachers Study. Fertility and Sterility. 2012;97:111-117. doi:10.1016/j.fertnstert.2011.10.004

21. Farahmand M, Ramezani Tehrani F, Simbar M, Mehrabi Y, Khalili D, Azizi F. Does metabolic syndrome or its components differ in naturally and surgically menopausal women? Climacteric. 2014;17:348-355. doi:10.3109/13697137.2013.856400

22. Howard BV, Kuller L, Langer R, Manson JE, Allen C, Assaf A. Risk of Cardiovascular Disease by Hysterectomy Status, With and Without Oophorectomy. Circulation. 2005;111:1462-1470. doi:10.1161/01.CIR.0000159344.21672.FD

23. Krul IM, Winden AWJOV, Janus CPM, et al. Cardiovascular Disease Risk After Treatment-Induced Premature Ovarian Insufficiency in Female Survivors of Hodgkin Lymphoma. J Am Coll Cardiol. 2018;72(25):3374-3375. doi:doi:10.1016/j.jacc.2018.10.030

24. Thong EP, Hart RJ, Teede HJ, Vincent AJ, Enticott JC. Increased mortality and non-cancer morbidity risk may be associated with early menopause and varies with aetiology. Maturitas. 2022;164:60-66. doi:10.1016/j.maturitas.2022.06.011

25. Tuesley KM, Protani MM, Webb PM, et al. Hysterectomy with and without oophorectomy and all-cause and cause-specific mortality. Am J Obstet Gynecol. Nov 2020;223(5):723.e1-723.e16. doi:10.1016/j.ajog.2020.04.037

26. Abbas SZ, Sangawan V, Das A, Pandey A. Assessment of cardiovascular risk in natural and surgical menopause. Indian Journal of Endocrinology and Metabolism. 2018;22:223. doi:10.4103/ijem.IJEM_620_17

27. Dorum A, Tonstad S, Liavaag AH, Michelsen TM, Hildrum B, Dahl AA. Bilateral oophorectomy before 50 years of age is significantly associated with the metabolic syndrome and Framingham risk score. Gynecologic Oncology. 2008;109:377-383. doi:10.1016/j.ygyno.2008.02.025

28. Indhavivadhana S, Rattanachaiyanont M, Wongvananurak T, Kanboon M, Techatraisak K, Leerasiri P. Predictors for metabolic syndrome in perimenopausal and postmenopausal Thai women. Climacteric. 2011;14:58-65. doi:10.3109/13697137.2010.481735

29. Jamali Z, Khalili P, Ayoobi F, Vatankhah H, Esmaeili-Nadimi A, Ranjbar FE. Type of menopause, age of menopause and cardiovascular disease: a cross-sectional study based on data from Rafsanjan cohort study. BMC Women's Health. 2024;24:626. doi:10.1186/s12905-024-03452-x

30. Mack WJ, Slater CC, Xiang M, Shoupe D, Lobo RA, Hodis HN. Elevated subclinical atherosclerosis associated with oophorectomy is related to time since menopause rather than type of menopause. Fertility and Sterility. 2004;82:391-397. doi:10.1016/j.fertnstert.2004.01.034

31. Netterlid A, Mörse H, Giwercman A, et al. Premature ovarian failure after childhood cancer and risk of metabolic syndrome: a cross-sectional analysis. European Journal of Endocrinology. 2021;185(1):67-75. doi:10.1530/eje-20-1275

32. Ou YJ, Lee JI, Huang SP, Chen SC, Geng JH, Su CH. Association between Menopause, Postmenopausal Hormone Therapy and Metabolic Syndrome. Journal of Clinical Medicine. 2023;12:4435. doi:10.3390/jcm12134435

33. Ozdemir S, Celik C, Gorkemli H, Kiyici A, Kaya B. Compared effects of surgical and natural menopause on climacteric symptoms, osteoporosis, and metabolic syndrome. International Journal of Gynecology & Obstetrics. 2009;106:57-61. doi:10.1016/j.ijgo.2009.03.016

34. Özkaya E, Cakir E, Okuyan E, Cakir C, Ustün G, Küçüközkan T. Comparison of the effects of surgical and natural menopause on carotid intima media thickness, osteoporosis, and homocysteine levels. Menopause. Jan 2011;18(1):73-6. doi:10.1097/gme.0b013e3181e5046d

© 2026 Armeni E. *JAMA Network Open*. This is an open access article distributed under the terms of the CC-BY-NC-ND License, which does not permit alteration or commercial use, including those for text and data mining, AI training, and similar technologies.

35. Tuysuzoglu FN, Ilhan GA, Yildizhan B. The impact of surgical menopause on metabolic syndrome, bone mineral density, and vasomotor symptoms. Clinical and Experimental Obstetrics & Gynecology. 2020;47doi:10.31083/j.ceog.2020.02.5037

36. Chen IJ, Shoupe D, Karim R, Stanczyk FZ, Kono N, Sriprasert I. The association of hysterectomy with or without ovarian conservation with subclinical atherosclerosis progression in healthy postmenopausal women. Menopause. 2023;30:692-702. doi:10.1097/GME.0000000000002192

© 2026 Armeni E. *JAMA Network Open*. This is an open access article distributed under the terms of the CC-BY-NC-ND License, which does not permit alteration or commercial use, including those for text and data mining, AI training, and similar technologies.

eAppendix.

Part 1. Eligibility criteria and comparator definitions

Criteria as registered in PROSPERO (CRD420251033146), applied to the cardiovascular scope of this report.

Population — included

- Women who experienced surgical intervention leading to menopause, specifically hysterectomy with bilateral oophorectomy, regardless of indication (e.g., cancer, endometriosis, benign conditions).
- Women with chemotherapy-induced ovarian insufficiency or senescence, whether temporary or permanent, occurring during or after cancer treatment.

Population — excluded

- Women who experienced spontaneous menopause prior to undergoing surgical treatment (e.g., hysterectomy or oophorectomy).
- Women who had spontaneous menopause before exposure to chemotherapy.
- Spontaneous early menopause or primary/premature ovarian insufficiency (POI) not related to medical or surgical intervention.
- Women undergoing hysterectomy without bilateral oophorectomy, unless subsequent menopause was confirmed to be iatrogenic.
- Studies focusing exclusively on natural menopause or non-iatrogenic causes of ovarian failure.

Exposures — included

- Surgical: hysterectomy with bilateral oophorectomy, prophylactic (e.g., BRCA mutation carriers) or therapeutic (e.g., endometriosis, fibroids, ovarian or endometrial cancer); other pelvic surgery resulting in permanent ovarian failure due to complete ovarian removal.
- Chemotherapy: gonadotoxic regimens known to induce ovarian failure or menopause, including alkylating agents, platinum-based compounds, anthracyclines, taxanes, antimetabolites, vinca alkaloids and combination regimens (e.g., CMF, AC-T, CHOP, BEACOPP); targeted therapies (e.g., imatinib) if associated with documented permanent ovarian insufficiency.
- GnRH agonists (e.g., goserelin, leuprolide) only where they result in permanent ovarian suppression consistent with menopause.

Exposures — excluded

- Spontaneous (natural) menopause, at a typical age or prematurely, prior to any surgical or chemotherapeutic intervention.
- Hysterectomy without bilateral oophorectomy, unless ovarian failure was clearly documented as a result of the procedure.
- Chemotherapy or targeted therapies not associated with ovarian failure, or where post-treatment menopausal status is unclear or not reported.
- GnRH agonists or antagonists used for temporary ovarian suppression, unless permanent menopause following treatment is clearly evidenced.
- Radiotherapy-induced menopause, unless the study provides explicit data isolating pelvic or gonadal radiation leading to confirmed menopause.
- Experimental or investigational agents not in standard clinical practice or without known gonadotoxicity.

Comparators

Eligible comparator groups were women with natural menopause, or age-matched women without oophorectomy or pelvic surgery (including hysterectomy-only groups where reported as a separate arm). women’s age at surgery — was fulfilled in part but not in full by any estimate entering the analyses.

Study designs — included

© 2026 Armeni E. *JAMA Network Open*. This is an open access article distributed under the terms of the CC-BY-NC-ND License, which does not permit alteration or commercial use, including those for text and data mining, AI training, and similar technologies.

- Randomized controlled trials; prospective and retrospective cohort studies; other observational designs (cross-sectional, case–control).

Study designs — excluded

- Case reports or small case series (n<10), unless highlighting rare but critical complications; animal or in vitro studies; opinion pieces, editorials or narrative reviews without original data; studies with mixed populations in which iatrogenic menopause outcomes are not separately reported.

Other restrictions

- English-language publications; published 1 January 2004 to 15 March 2026.
- Outcomes eligible for this report: cardiovascular morbidity (composite cardiovascular events, coronary heart disease, stroke, heart failure, metabolic syndrome), cardiovascular mortality and all-cause mortality; quantitative pooling restricted to studies reporting hazard ratios, with all other eligible estimates synthesized narratively

© 2026 Armeni E. *JAMA Network Open*. This is an open access article distributed under the terms of the CC-BY-NC-ND License, which does not permit alteration or commercial use, including those for text and data mining, AI training, and similar technologies.

Part 2. Search strategies by database

All databases were last searched in duplicate on 15 March 2026. Limits applied in every database: English language; publication date 1 January 2004 to 15 March 2026. Reference lists of eligible studies and of relevant reviews were additionally screened to identify further records. Records identified per stage are shown in Figure 1.

PubMed (NLM)

Line	Query	Notes
#1	"iatrogenic menopause"[tiab] OR "surgical menopause"[tiab] OR "chemotherapy-induced menopause"[tiab] OR "radiotherapy-induced menopause"[tiab] OR "premature menopause"[tiab] OR "premature ovarian insufficiency"[tiab] OR "premature ovarian failure"[tiab] OR "early menopause"[tiab] OR "induced menopause"[tiab] OR "bilateral oophorectomy"[tiab] OR "Menopause, Premature"[Mesh] OR "Ovariectomy"[Mesh] OR "Primary Ovarian Insufficiency"[Mesh]	Exposure: iatrogenic menopause
#2	"cardiovascular disease"[tiab] OR "cardiovascular diseases"[tiab] OR "coronary heart disease"[tiab] OR "coronary artery disease"[tiab] OR "myocardial infarction"[tiab] OR "stroke"[tiab] OR "cerebrovascular disease"[tiab] OR "heart failure"[tiab] OR "metabolic syndrome"[tiab] OR "cardiovascular mortality"[tiab] OR "all-cause mortality"[tiab] OR "mortality"[tiab] OR "cardiovascular events"[tiab] OR "Cardiovascular Diseases"[Mesh] OR "Stroke"[Mesh] OR "Myocardial Infarction"[Mesh] OR "Heart Failure"[Mesh] OR "Metabolic Syndrome"[Mesh] OR "Mortality"[Mesh]	Outcomes: cardiovascular morbidity and mortality
#3	#1 AND #2	Combine exposure AND outcome
#4	#3 AND ("2004/01/01"[Date - Publication] : "2026/03/15"[Date - Publication]) AND english[Language]	Limits: date, English

MEDLINE (Ovid)

Line	Query	Notes
#1	"iatrogenic menopause".ti,ab,kw. or "surgical menopause".ti,ab,kw. or "chemotherapy-induced menopause".ti,ab,kw. or "radiotherapy-induced menopause".ti,ab,kw. or "premature menopause".ti,ab,kw. or "premature ovarian insufficiency".ti,ab,kw. or "premature ovarian failure".ti,ab,kw. or "early menopause".ti,ab,kw. or "induced menopause".ti,ab,kw. or "bilateral oophorectomy".ti,ab,kw.	Exposure, free text (title/abstract/keyword)
#2	exp Menopause, Premature/ or exp Ovariectomy/ or exp Primary Ovarian Insufficiency/	Exposure, MeSH (exploded)
#3	1 or 2	Exposure set
#4	"cardiovascular disease".ti,ab,kw. or "cardiovascular diseases".ti,ab,kw. or "coronary heart disease".ti,ab,kw. or "coronary artery disease".ti,ab,kw. or "myocardial infarction".ti,ab,kw. or "stroke".ti,ab,kw. or "cerebrovascular disease".ti,ab,kw. or "heart failure".ti,ab,kw. or "metabolic syndrome".ti,ab,kw. or "cardiovascular mortality".ti,ab,kw. or "all-cause mortality".ti,ab,kw. or "mortality".ti,ab,kw. or "cardiovascular events".ti,ab,kw.	Outcomes, free text
#5	exp Cardiovascular Diseases/ or exp Stroke/ or exp Myocardial Infarction/ or exp Heart Failure/ or exp Metabolic Syndrome/ or exp Mortality/	Outcomes, MeSH (exploded)

© 2026 Armeni E. *JAMA Network Open*. This is an open access article distributed under the terms of the CC-BY-NC-ND License, which does not permit alteration or commercial use, including those for text and data mining, AI training, and similar technologies.

#6	4 or 5	Outcome set
#7	3 and 6	Combine
#8	limit 7 to (english language and yr="2004 - 2026")	Limits; entries after 15 Mar 2026 removed at screening

Embase (Ovid)

Line	Query	Notes
#1	"iatrogenic menopause".ti,ab,kw. or "surgical menopause".ti,ab,kw. or "chemotherapy-induced menopause".ti,ab,kw. or "radiotherapy-induced menopause".ti,ab,kw. or "premature menopause".ti,ab,kw. or "premature ovarian insufficiency".ti,ab,kw. or "premature ovarian failure".ti,ab,kw. or "early menopause".ti,ab,kw. or "induced menopause".ti,ab,kw. or "bilateral oophorectomy".ti,ab,kw.	Exposure, free text
#2	exp premature menopause/ or exp ovariectomy/ or exp premature ovarian failure/	Exposure, Emtree (exploded)
#3	1 or 2	Exposure set
#4	"cardiovascular disease".ti,ab,kw. or "cardiovascular diseases".ti,ab,kw. or "coronary heart disease".ti,ab,kw. or "coronary artery disease".ti,ab,kw. or "myocardial infarction".ti,ab,kw. or "stroke".ti,ab,kw. or "cerebrovascular disease".ti,ab,kw. or "heart failure".ti,ab,kw. or "metabolic syndrome".ti,ab,kw. or "cardiovascular mortality".ti,ab,kw. or "all-cause mortality".ti,ab,kw. or "mortality".ti,ab,kw. or "cardiovascular events".ti,ab,kw.	Outcomes, free text
#5	exp cardiovascular disease/ or exp cerebrovascular accident/ or exp heart infarction/ or exp heart failure/ or exp metabolic syndrome X/ or exp mortality/	Outcomes, Emtree (exploded)
#6	4 or 5	Outcome set
#7	3 and 6	Combine
#8	limit 7 to (english language and yr="2004 - 2026" and embase)	Limits; Embase-native records

CENTRAL (Cochrane Library)

Line	Query	Notes
#1	("iatrogenic menopause" OR "surgical menopause" OR "chemotherapy-induced menopause" OR "radiotherapy-induced menopause" OR "premature menopause" OR "premature ovarian insufficiency" OR "premature ovarian failure" OR "early menopause" OR "induced menopause" OR "bilateral oophorectomy"):ti,ab,kw	Exposure, free text
#2	MeSH descriptor: [Menopause, Premature] explode all trees	Exposure, MeSH
#3	MeSH descriptor: [Ovariectomy] explode all trees	Exposure, MeSH
#4	#1 OR #2 OR #3	Exposure set
#5	("cardiovascular disease" OR "cardiovascular diseases" OR "coronary heart disease" OR "coronary artery disease" OR "myocardial infarction" OR "stroke" OR "cerebrovascular disease" OR "heart	Outcomes, free text

© 2026 Armeni E. *JAMA Network Open*. This is an open access article distributed under the terms of the CC-BY-NC-ND License, which does not permit alteration or commercial use, including those for text and data mining, AI training, and similar technologies.

	failure" OR "metabolic syndrome" OR "cardiovascular mortality" OR "all-cause mortality" OR "mortality" OR "cardiovascular events"):ti,ab,kw	
#6	MeSH descriptor: [Cardiovascular Diseases] explode all trees	Outcomes, MeSH
#7	#5 OR #6	Outcome set
#8	#4 AND #7, with publication date from Jan 2004 to Mar 2026, in Trials	Combine + limits (custom date range in Cochrane Library)

CINAHL (EBSCOhost)

Line	Query	Notes
#1	TI ("iatrogenic menopause" OR "surgical menopause" OR "chemotherapy-induced menopause" OR "radiotherapy-induced menopause" OR "premature menopause" OR "premature ovarian insufficiency" OR "premature ovarian failure" OR "early menopause" OR "induced menopause" OR "bilateral oophorectomy") OR AB ("iatrogenic menopause" OR "surgical menopause" OR "chemotherapy-induced menopause" OR "radiotherapy-induced menopause" OR "premature menopause" OR "premature ovarian insufficiency" OR "premature ovarian failure" OR "early menopause" OR "induced menopause" OR "bilateral oophorectomy")	Exposure, free text
#2	(MH "Menopause, Premature+") OR (MH "Ovariectomy+")	Exposure, CINAHL headings (exploded)
#3	S1 OR S2	Exposure set
#4	TI ("cardiovascular disease" OR "cardiovascular diseases" OR "coronary heart disease" OR "coronary artery disease" OR "myocardial infarction" OR "stroke" OR "cerebrovascular disease" OR "heart failure" OR "metabolic syndrome" OR "cardiovascular mortality" OR "all-cause mortality" OR "mortality" OR "cardiovascular events") OR AB ("cardiovascular disease" OR "cardiovascular diseases" OR "coronary heart disease" OR "coronary artery disease" OR "myocardial infarction" OR "stroke" OR "cerebrovascular disease" OR "heart failure" OR "metabolic syndrome" OR "cardiovascular mortality" OR "all-cause mortality" OR "mortality" OR "cardiovascular events")	Outcomes, free text
#5	(MH "Cardiovascular Diseases+") OR (MH "Stroke+") OR (MH "Myocardial Infarction+") OR (MH "Heart Failure+") OR (MH "Metabolic Syndrome X") OR (MH "Mortality+")	Outcomes, headings (exploded)
#6	S4 OR S5	Outcome set
#7	S3 AND S6	Combine
#8	S7; Limiters: English Language; Published Date 20040101-20260315	Limits

PsycINFO (Ovid)

Line	Query	Notes
#1	"iatrogenic menopause".ti,ab,kw. or "surgical menopause".ti,ab,kw. or "chemotherapy-induced menopause".ti,ab,kw. or "radiotherapy-induced menopause".ti,ab,kw. or "premature menopause".ti,ab,kw. or "premature ovarian insufficiency".ti,ab,kw. or "premature ovarian	Exposure, free text

© 2026 Armeni E. *JAMA Network Open*. This is an open access article distributed under the terms of the CC-BY-NC-ND License, which does not permit alteration or commercial use, including those for text and data mining, AI training, and similar technologies.

	failure".ti,ab,kw. or "early menopause".ti,ab,kw. or "induced menopause".ti,ab,kw. or "bilateral oophorectomy".ti,ab,kw.	
#2	exp Menopause/	Exposure, Thesaurus (menopause; premature forms captured by free text)
#3	1 or 2	Exposure set
#4	"cardiovascular disease".ti,ab,kw. or "cardiovascular diseases".ti,ab,kw. or "coronary heart disease".ti,ab,kw. or "coronary artery disease".ti,ab,kw. or "myocardial infarction".ti,ab,kw. or "stroke".ti,ab,kw. or "cerebrovascular disease".ti,ab,kw. or "heart failure".ti,ab,kw. or "metabolic syndrome".ti,ab,kw. or "cardiovascular mortality".ti,ab,kw. or "all-cause mortality".ti,ab,kw. or "mortality".ti,ab,kw. or "cardiovascular events".ti,ab,kw.	Outcomes, free text
#5	exp Cardiovascular Disorders/ or exp Cerebrovascular Accidents/ or exp Metabolic Syndrome/ or exp "Death and Dying"/	Outcomes, Thesaurus
#6	4 or 5	Outcome set
#7	3 and 6	Combine
#8	limit 7 to (english language and yr="2004 - 2026")	Limits

Scopus (Elsevier)

Line	Query	Notes
#1	TITLE-ABS-KEY ("iatrogenic menopause" OR "surgical menopause" OR "chemotherapy-induced menopause" OR "radiotherapy-induced menopause" OR "premature menopause" OR "premature ovarian insufficiency" OR "premature ovarian failure" OR "early menopause" OR "induced menopause" OR "bilateral oophorectomy")	Exposure
#2	TITLE-ABS-KEY ("cardiovascular disease" OR "cardiovascular diseases" OR "coronary heart disease" OR "coronary artery disease" OR "myocardial infarction" OR "stroke" OR "cerebrovascular disease" OR "heart failure" OR "metabolic syndrome" OR "cardiovascular mortality" OR "all-cause mortality" OR "mortality" OR "cardiovascular events")	Outcomes
#3	#1 AND #2	Combine
#4	#3 AND PUBYEAR > 2003 AND PUBYEAR < 2027 AND LIMIT-TO (LANGUAGE, "English")	Limits; entries after 15 Mar 2026 removed at screening

© 2026 Armeni E. *JAMA Network Open*. This is an open access article distributed under the terms of the CC-BY-NC-ND License, which does not permit alteration or commercial use, including those for text and data mining, AI training, and similar technologies.

Part 3. Reports not retrieved and reports excluded at full-text assessment, with reasons

Of 242 reports sought for retrieval, 10 could not be retrieved and 196 were excluded after full-text assessment: wrong outcomes (n=108); wrong study design (n=46); wrong comparator (n=16); wrong patient population (n=12); wrong intervention (n=5); non-English language (n=3); not peer-reviewed (n=2); wrong definition (n=2); no comparator (n=1); wrong comparator and wrong study design (n=1). All 206 reports are listed below by exclusion reason and year of publication; the 10 unretrievable reports appear under "No full text availability."

Study	Title	Journal, year	Exclusion reason
Cohen 2012	Non-cancer endpoints in BRCA1/2 carriers after risk-reducing salpingo-oophorectomy.	Fam Cancer, 2012	No comparator
Albright 1990	Temporal patterns of hot flashes in natural and surgically-induced menopause.	Progress in clinical and biological research, 1990	No full text availability
Gnudi 1990	Evaluation of the relative rates of bone mineral content loss in postmenopause due to both estrogen deficiency and ageing.	Bollettino della Società italiana di biologia sperimentale, 1990	No full text availability
Taechakraichana 1997	Bone loss in oophorectomized Thai women	1997	No full text availability
Aydemir 1999	Anxiety and depression in the menopausal period.	Turk Psikiyatri Dergisi, 1999	No full text availability
Begum 2001	Effect of oestrogen replacement therapy on bone mass in post-menopausal Bangladeshi women	Bangladesh Medical Research Council Bulletin, 2001	No full text availability
Sheridan 2011	Chemotherapy-induced versus natural menopause: A comparison of menopausal symptoms and sexual functioning.	Dissertation Abstracts International: Section B: The Sciences and Engineering, 2011	No full text availability
Appiah 2015	Cardiovascular disease among diabetic women with bilateral oophorectomy.	Dissertation Abstracts International: Section B: The Sciences and Engineering, 2015	No full text availability
Mehedintu 2016	Quality of life and psychosomatic symptoms of menopause-national multicenter study	2016	No full text availability
Chen 2022	The long-term effects of bilateral oophorectomy on progression of subclinical atherosclerosis in healthy postmenopausal women	2022	No full text availability
Gulbahar 2022	Comparison of sexual function between women with iatrogenic and natural menopause.	Revista internacional de andrologia, 2022	No full text availability
Varma 2005	The relationship between depression and anxiety levels and sexual satisfaction for natural and surgical menopause.	Klinik Psikiyatri Dergisi: The Journal of Clinical Psychiatry, 2005	Non-English language
Varma 2006	Menopause: Relationship between climacteric symptoms and sexual satisfaction.	Yeni Symposium: psikiyatri, noroloji ve davranis bilimleri dergisi, 2006	Non-English language
Farahmand 2021	Comparison of the prevalence rates of metabolic disorders between two groups of women undergone surgically induced	Scientific Journal of Kurdistan University of Medical Sciences, 2021	Non-English language

© 2026 Armeni E. *JAMA Network Open*. This is an open access article distributed under the terms of the CC-BY-NC-ND License, which does not permit alteration or commercial use, including those for text and data mining, AI training, and similar technologies.

	menopause by hysterectomy and bilateral oophorectomy: A population-based study		
Moadel 1996	Psychological distress of prematurely menopausal cancer survivors.	Dissertation Abstracts International: Section B: The Sciences and Engineering, 1996	Not peer-reviewed
Schwab 1998	Risk of coronary heart disease, dietary fat modification, stages of change, and self-efficacy in surgical and natural postmenopausal women.	Dissertation Abstracts International: Section B: The Sciences and Engineering, 1998	Not peer-reviewed
Hreshchyshyn 1988	Effects of natural menopause, hysterectomy, and oophorectomy on lumbar spine and femoral neck bone densities	Obstetrics and Gynecology, 1988	Wrong comparator
Parazzini 1997	Hysterectomy, oophorectomy in premenopause, and risk of breast cancer.	Obstet Gynecol, 1997	Wrong comparator
Nappi 1999	Memory functioning at menopause: Impact of age in ovariectomized women	Gynecologic and Obstetric Investigation, 1999	Wrong comparator
Falkeborn 2000	Risk of myocardial infarction after oophorectomy and hysterectomy	Journal of Clinical Epidemiology, 2000	Wrong comparator
McPhail 2000	Acute menopause symptoms during adjuvant systemic treatment for breast cancer: a case-control study.	Cancer Nursing, 2000	Wrong comparator
Abbott 2002	Patterns of use of hormone replacement therapy in one million women in Britain, 1996-2000	BJOG: An International Journal of Obstetrics and Gynaecology, 2002	Wrong comparator
Alessandri 2007	Morphological and functional changes of cardiovascular system in postmenopausal women.	Eur Rev Med Pharmacol Sci, 2007	Wrong comparator
Blümel 2011	A large multinational study of vasomotor symptom prevalence, duration, and impact on quality of life in middle-aged women.	Menopause, 2011	Wrong comparator
McCarthy 2012	Bilateral oophorectomy, bodymass index, and mortality in U.S. women aged 40 years and older	Cancer Prevention Research, 2012	Wrong comparator
Mendoza 2016	Factors determining the use of hormonal therapy and phytotherapy in Spanish postmenopausal women.	Climacteric, 2016	Wrong comparator
Fernández-Alonso 2017	Application of the 21-item Vulvovaginal Symptoms Questionnaire in postmenopausal Spanish women	Menopause, 2017	Wrong comparator
Steenbeek 2021	Association of Salpingectomy With Delayed Oophorectomy Versus Salpingo-oophorectomy With Quality of Life in BRCA1/2 Pathogenic Variant Carriers: A Nonrandomized Controlled Trial.	JAMA Oncol, 2021	Wrong comparator
Wilson 2022	Associations of protective factors with physical function in women with a hysterectomy: A prospective Australian cohort study.	Maturitas, 2022	Wrong comparator

© 2026 Armeni E. *JAMA Network Open*. This is an open access article distributed under the terms of the CC-BY-NC-ND License, which does not permit alteration or commercial use, including those for text and data mining, AI training, and similar technologies.

Shen 2023	Association between hysterectomy status and stroke risk and cause-specific and all-cause mortality	Frontiers in Neurology, 2023	Wrong comparator
Beekman 2024	Coronary Artery Calcium Scores After Prophylactic Premenopausal Bilateral Salpingo-Oophorectomy	JACC: CardioOncology, 2024	Wrong comparator
Beekman 2026	Long-term outcomes of surgical menopause after risk-reducing salpingo-oophorectomy: results of the HARMOny study.	Maturitas, 2026	Wrong comparator
Park 2021	Incidence and Risk Factors of Cardio-Cerebrovascular Disease in Korean Menopausal Women: A Retrospective Observational Study using the Korean Genome and Epidemiology Study data.	Asian nursing research, 2021	Wrong comparator; Wrong study design
Nichols 2013	Pre-diagnosis oophorectomy, estrogen therapy and mortality in a cohort of women diagnosed with breast cancer.	Breast cancer research : BCR, 2013	Wrong definition
Peters 2018	Women's reproductive factors and incident cardiovascular disease in the UK Biobank.	Heart, 2018	Wrong definition
Ng 1993	Estradiol administered in a percutaneous gel for the prevention of postmenopausal bone loss	1993	Wrong intervention
Takahashi 2000	Safety and efficacy of oestriol for symptoms of natural or surgically induced menopause.	Hum Reprod, 2000	Wrong intervention
Zacharieva 2002	Effect of different hormone replacement therapy regimens on circadian blood pressure profile and active renin in postmenopausal women	Gynecological Endocrinology, 2002	Wrong intervention
DeLeo 2003	Effects of estradiol alone or in combination with cyproterone acetate on carotid artery pulsatility index in postmenopausal women	Maturitas, 2003	Wrong intervention
Chiang 2021	Diabetes mellitus risk after hysterectomy: A population-based retrospective cohort study.	Medicine (Baltimore), 2021	Wrong intervention
Albright 1989	Circadian rhythms in hot flashes in natural and surgically-induced menopause	Chronobiology International, 1989	Wrong outcomes
Cauley 1990	Prevalence and determinants of estrogen replacement therapy in elderly women.	Am J Obstet Gynecol, 1990	Wrong outcomes
Ratcliffe 1992	Bone mineral density (BMD) in patients with lymphoma: the effects of chemotherapy, intermittent corticosteroids and premature menopause.	Hematol Oncol, 1992	Wrong outcomes
Schouten 1992	Natural menopause, oophorectomy, hysterectomy and the risk of osteoarthritis of the dip joints	Scandinavian Journal of Rheumatology, 1992	Wrong outcomes
Kritz-Silverstein 1993	Early menopause, number of reproductive years, and bone mineral density in postmenopausal women	American Journal of Public Health, 1993	Wrong outcomes

© 2026 Armeni E. *JAMA Network Open*. This is an open access article distributed under the terms of the CC-BY-NC-ND License, which does not permit alteration or commercial use, including those for text and data mining, AI training, and similar technologies.

Busch 1994	Menopausal transition and psychological distress in a nationally representative sample: Is menopause associated with psychological distress?	Journal of Aging and Health, 1994	Wrong outcomes
Kritz-Silverstein 1994	Hysterectomy, oophorectomy and depression in older women	Journal of Women's Health, 1994	Wrong outcomes
Everson 1995	Effects of Surgical Menopause on Psychological Characteristics and Lipid Levels: The Healthy Women Study	Health Psychology, 1995	Wrong outcomes
Akahoshi 1996	Effects of Menopause on Trends of Serum Cholesterol, Blood Pressure, and Body Mass Index	Circulation, 1996	Wrong outcomes
Yildiz 1996	Bone loss rate in the lumbar spine: A comparison between natural and surgically induced menopause	International Journal of Gynecology and Obstetrics, 1996	Wrong outcomes
Zeigler-Johnson 1998	Subclinical atherosclerosis in relation to hysterectomy status in black women	Stroke, 1998	Wrong outcomes
Castelo-Branco 1999	Long-term postmenopausal hormone replacement therapy effects on bone mass: differences between surgical and spontaneous patients.	Eur J Obstet Gynecol Reprod Biol, 1999	Wrong outcomes
Kritz-Silverstein 2000	Prevalence and clustering of menopausal symptoms in older women by hysterectomy and oophorectomy status	Journal of Women's Health and Gender-Based Medicine, 2000	Wrong outcomes
Mazer 2000	The brief index of sexual functioning for women (BISF-W): a new scoring algorithm and comparison of normative and surgically menopausal populations.	Menopause, 2000	Wrong outcomes
Adamopoulos 2002	Age at menopause and prevalence of its different types in contemporary Greek women	Menopause, 2002	Wrong outcomes
Farrag 2002	Effect of surgical menopause on cognitive functions	Dementia and Geriatric Cognitive Disorders, 2002	Wrong outcomes
Kritz-Silverstein 2002	Hysterectomy, oophorectomy, and cognitive function in older women	Journal of the American Geriatrics Society, 2002	Wrong outcomes
Kritz-Silverstein 2002	Hysterectomy status and life satisfaction in older women	Journal of Women's Health and Gender-Based Medicine, 2002	Wrong outcomes
Hadjidakis 2003	Bone density patterns after normal and premature menopause	Maturitas, 2003	Wrong outcomes
García-Pérez 2004	Bone Turnover Markers and PTH Levels in Surgical Versus Natural Menopause	Calcified Tissue International, 2004	Wrong outcomes
Kritz-Silverstein 2004	Hysterectomy status, estrogen use and quality of life in older women: The Rancho Bernardo study	Quality of Life Research, 2004	Wrong outcomes
Kritz-Silverstein 2004	Hysterectomy and oophorectomy are unrelated to bone loss in older women	Maturitas, 2004	Wrong outcomes
Takamatsu 2004	Evaluation of depressive conditions among Japanese patients at a menopause clinic	Journal of Obstetrics and Gynaecology Research, 2004	Wrong outcomes

© 2026 Armeni E. *JAMA Network Open*. This is an open access article distributed under the terms of the CC-BY-NC-ND License, which does not permit alteration or commercial use, including those for text and data mining, AI training, and similar technologies.

Hassa 2005	Associated factors with urogenital score in natural and surgical menopause	Maturitas, 2005	Wrong outcomes
Bednarek-Tupikowska 2006	Serum leptin concentrations in pre- and postmenopausal women on sex hormone therapy.	Gynecol Endocrinol, 2006	Wrong outcomes
Dennerstein 2006	Hypoactive sexual desire disorder in menopausal women: A survey of Western European women	Journal of Sexual Medicine, 2006	Wrong outcomes
Drozdzowska 2006	Quantitative ultrasound measurements at the calcaneus in natural and surgically induced menopause	Maturitas, 2006	Wrong outcomes
Finch 2006	Salpingo-oophorectomy and the risk of ovarian, fallopian tube, and peritoneal cancers in women with a BRCA1 or BRCA2 Mutation	2006	Wrong outcomes
Gallicchio 2006	Type of menopause, patterns of hormone therapy use, and hot flashes	Fertility and Sterility, 2006	Wrong outcomes
Rocca 2007	Increased risk of cognitive impairment or dementia in women who underwent oophorectomy before menopause	Neurology, 2007	Wrong outcomes
Rocca 2008	Long-term risk of depressive and anxiety symptoms after early bilateral oophorectomy	Menopause, 2008	Wrong outcomes
Rohl 2008	Bilateral oophorectomy and depressive symptoms 12 months after hysterectomy	American Journal of Obstetrics and Gynecology, 2008	Wrong outcomes
Benshushan 2009	Climacteric symptoms in women undergoing risk-reducing bilateral salpingo-oophorectomy	Climacteric, 2009	Wrong outcomes
MarFan 2009	Menopausal symptoms in women undergoing chemotherapy-induced and natural menopause: A prospective controlled study	Annals of Oncology, 2009	Wrong outcomes
Rivera 2009	Increased mortality for neurological and mental diseases following early bilateral oophorectomy	Neuroepidemiology, 2009	Wrong outcomes
Bhattacharya 2010	A comparison of health-related quality of life (HRQOL) after natural and surgical menopause	Maturitas, 2010	Wrong outcomes
Faria 2010	Ankle stiffness in postmenopausal women: Influence of hormone therapy and menopause nature	Climacteric, 2010	Wrong outcomes
Phung 2010	Hysterectomy, oophorectomy and risk of dementia: A nationwide historical cohort study	Dementia and Geriatric Cognitive Disorders, 2010	Wrong outcomes
Alarslan 2011	Androgens and sexual dysfunction in naturally and surgically menopausal women	Journal of Obstetrics and Gynaecology Research, 2011	Wrong outcomes
Kim 2011	Menopause and risk of diabetes in the Diabetes Prevention Program.	Menopause, 2011	Wrong outcomes
Pokoradi 2011	Factors associated with age of onset and type of menopause in a cohort of UK women	American Journal of Obstetrics and Gynecology, 2011	Wrong outcomes

© 2026 Armeni E. *JAMA Network Open*. This is an open access article distributed under the terms of the CC-BY-NC-ND License, which does not permit alteration or commercial use, including those for text and data mining, AI training, and similar technologies.

Gavin 2012	Hysterectomy is associated with large artery stiffening in estrogen-deficient postmenopausal women	Menopause, 2012	Wrong outcomes
Gibson 2012	Mood symptoms after natural menopause and hysterectomy with and without bilateral oophorectomy among women in midlife.	Obstet Gynecol, 2012	Wrong outcomes
Tom 2012	Menopausal characteristics and physical functioning in older adulthood in the National Health and Nutrition Examination Survey III	Menopause, 2012	Wrong outcomes
Topatan 2012	Symptoms Experienced by Women Who Enter Into Natural and Surgical Menopause and Their Relation to Sexual Functions.	Health Care for Women International, 2012	Wrong outcomes
Vesco 2012	Surgical menopause and nonvertebral fracture risk among older US women	Menopause, 2012	Wrong outcomes
Gibson 2013	Body mass index following natural menopause and hysterectomy with and without bilateral oophorectomy	International Journal of Obesity, 2013	Wrong outcomes
Appiah 2014	Bilateral oophorectomy and the risk of incident diabetes in postmenopausal women	Diabetes Care, 2014	Wrong outcomes
Boggs 2014	Bilateral oophorectomy and risk of cancer in African American women	Cancer Causes and Control, 2014	Wrong outcomes
Bove 2014	Age at surgical menopause influences cognitive decline and Alzheimer pathology in older women	Neurology, 2014	Wrong outcomes
Imtiaz 2014	Oophorectomy, hysterectomy, and risk of Alzheimer's disease: A nationwide case-control study	Journal of Alzheimer's Disease, 2014	Wrong outcomes
Vajaranant 2014	Risk of glaucoma after early bilateral oophorectomy	Menopause, 2014	Wrong outcomes
Appiah 2015	Cardiovascular disease among women with and without diabetes mellitus and bilateral oophorectomy.	Diabetes Res Clin Pract, 2015	Wrong outcomes
Kokcu 2015	Does Surgical Menopause Affect Sexual Performance Differently from Natural Menopause?	Journal of Sexual Medicine, 2015	Wrong outcomes
Kurita 2016	Association of bilateral oophorectomy with cognitive function in healthy, postmenopausal women	Fertility and Sterility, 2016	Wrong outcomes
Matsuo 2016	Surgical menopause and increased risk of nonalcoholic fatty liver disease in endometrial cancer	Menopause, 2016	Wrong outcomes
Rocca 2016	Accelerated Accumulation of Multimorbidity After Bilateral Oophorectomy: A Population-Based Cohort Study	Mayo Clinic Proceedings, 2016	Wrong outcomes
Chemaitilly 2017	Premature Ovarian Insufficiency in Childhood Cancer Survivors: A Report From the St. Jude Lifetime Cohort.	The Journal of clinical endocrinology and metabolism, 2017	Wrong outcomes
Rocca 2017	Bilateral Oophorectomy and Accelerated Aging: Cause or Effect?	Journals of Gerontology - Series A Biological Sciences and Medical Sciences, 2017	Wrong outcomes

© 2026 Armeni E. *JAMA Network Open*. This is an open access article distributed under the terms of the CC-BY-NC-ND License, which does not permit alteration or commercial use, including those for text and data mining, AI training, and similar technologies.

Rocca 2017	Adverse childhood or adult experiences and risk of bilateral oophorectomy: A population-based case-control study	BMJ Open, 2017	Wrong outcomes
Giordano 2018	How long is the ovary relevant for synthesis of steroids after menopause?	Gynecological Endocrinology, 2018	Wrong outcomes
Huang 2018	Type of Menopause, Age at Menopause, and Risk of Developing Obstructive Sleep Apnea in Postmenopausal Women	American Journal of Epidemiology, 2018	Wrong outcomes
Huang 2018	Surgical Menopause and Frailty Risk in Community-Dwelling Older Women: Study of Osteoporotic Fractures	Journal of the American Geriatrics Society, 2018	Wrong outcomes
Kattah 2018	CKD in patients with bilateral oophorectomy	Clinical Journal of the American Society of Nephrology, 2018	Wrong outcomes
Rocca 2018	Long-term risk of depressive and anxiety symptoms after early bilateral oophorectomy.	Menopause, 2018	Wrong outcomes
Waetjen 2018	Factors associated with developing vaginal dryness symptoms in women transitioning through menopause: a longitudinal study.	Menopause, 2018	Wrong outcomes
Wilson 2018	Hysterectomy and incidence of depressive symptoms in midlife women: The Australian Longitudinal Study on Women's Health	Epidemiology and Psychiatric Sciences, 2018	Wrong outcomes
Costas 2019	Reproductive Factors, Exogenous Hormone Use, and Risk of B-Cell Non-Hodgkin Lymphoma in a Cohort of Women From the European Prospective Investigation Into Cancer and Nutrition.	Am J Epidemiol, 2019	Wrong outcomes
ZilioRech 2019	Sexual Function Under Adequate Estrogen Therapy in Women After Oophorectomy Versus Natural Menopause.	J Womens Health (Larchmt), 2019	Wrong outcomes
Bıldırcın 2020	Does type of menopause affect the sex lives of women?	Medical Science Monitor, 2020	Wrong outcomes
Demir 2020	The relationship of menopausal symptoms with the type of menopause and lipid levels	Przeglad Menopauzalny, 2020	Wrong outcomes
Roberson 2020	Premenopausal gynecologic surgery and survival among black and white women with breast cancer	Cancer Causes and Control, 2020	Wrong outcomes
SertelMeyvaci 2020	Effect of surgical and natural menopause on proximal femur morphometry in obese women	Annals of Anatomy, 2020	Wrong outcomes
Gernier 2021	Menopausal symptoms in epithelial ovarian cancer survivors: a GINECO VIVROVAIRE2 study.	Gynecologic oncology, 2021	Wrong outcomes
Hickey 2021	What happens after menopause? (WHAM): A prospective controlled study of depression and anxiety up to 12 months after premenopausal risk-reducing bilateral salpingo-oophorectomy.	Gynecol Oncol, 2021	Wrong outcomes

© 2026 Armeni E. *JAMA Network Open*. This is an open access article distributed under the terms of the CC-BY-NC-ND License, which does not permit alteration or commercial use, including those for text and data mining, AI training, and similar technologies.

Honigberg 2021	Premature Menopause, Clonal Hematopoiesis, and Coronary Artery Disease in Postmenopausal Women.	Circulation, 2021	Wrong outcomes
Islam 2021	A prospective controlled study of sexual function and sexually related personal distress up to 12 months after premenopausal risk-reducing bilateral salpingo-oophorectomy	Menopause, 2021	Wrong outcomes
Mozumdar 2021	Prevalence and Associates of Natural Menopause and Surgical Menopause Among Indian Women Aged 30 to 49 Years: An Analysis of the National Family Health Survey	Women's Reproductive Health, 2021	Wrong outcomes
Rocca 2021	Association of Premenopausal Bilateral Oophorectomy With Cognitive Performance and Risk of Mild Cognitive Impairment.	JAMA network open, 2021	Wrong outcomes
Su 2021	Assessment of menopausal symptoms and quality of life in women with premature ovarian failure after hematopoietic stem-cell transplantation for hematologic diseases	Menopause, 2021	Wrong outcomes
UtkanKarasu 2021	Comparison of osteoporotic fracture risk in surgical and natural menopausal patients	Turkish Journal of Physical Medicine and Rehabilitation, 2021	Wrong outcomes
Welten 2021	Age at Menopause and Risk of Ischemic and Hemorrhagic Stroke.	Stroke, 2021	Wrong outcomes
Brauner 2022	The long-term association between bilateral oophorectomy and depression: a prospective cohort study.	Menopause (New York, N.Y.), 2022	Wrong outcomes
Buyuk 2022	Anogenital index and bone mineral density associations after natural and surgical menopause: a preliminary study.	Revista da Associacao Medica Brasileira (1992), 2022	Wrong outcomes
Huo 2022	Risk of de novo cancer after premenopausal bilateral oophorectomy.	American journal of obstetrics and gynecology, 2022	Wrong outcomes
Moss 2022	What happens after menopause? (WHAM): A prospective controlled study of symptom profiles up to 12 months after pre-menopausal risk-reducing salpingo-oophorectomy.	Gynecologic oncology, 2022	Wrong outcomes
Ozgedik 2022	Is there any difference in hearing function between surgical and natural menopause?.	Women & health, 2022	Wrong outcomes
Shin 2022	Age at menopause and risk of heart failure and atrial fibrillation: a nationwide cohort study.	European heart journal, 2022	Wrong outcomes
Uldbjerg 2022	Oophorectomy and rate of dementia: a prospective cohort study.	Menopause (New York, N.Y.), 2022	Wrong outcomes
Zhai 2022	Early natural menopause is associated with poor lung health and increased mortality among female smokers.	American journal of obstetrics and gynecology, 2022	Wrong outcomes
Gumussoy 2023	Effects of Surgical and Natural Menopause on Body Image, Self-Esteem, and Dyadic Adjustment: A Descriptive and Comparative Study.	Clinical nursing research, 2023	Wrong outcomes

© 2026 Armeni E. *JAMA Network Open*. This is an open access article distributed under the terms of the CC-BY-NC-ND License, which does not permit alteration or commercial use, including those for text and data mining, AI training, and similar technologies.

Hao 2023	Age at menopause and all-cause and cause-specific dementia: a prospective analysis of the UK Biobank cohort.	Human reproduction (Oxford, England), 2023	Wrong outcomes
Huebner 2023	Arthralgia in female Masters weightlifters.	BMC musculoskeletal disorders, 2023	Wrong outcomes
Korkmaz 2023	Comparison of plasma oxytocin level in women with natural and surgical menopause.	Endocrine, 2023	Wrong outcomes
Mielke 2023	Long-term effects of premenopausal bilateral oophorectomy with or without hysterectomy on physical aging and chronic medical conditions.	Menopause (New York, N.Y.), 2023	Wrong outcomes
Page 2023	Natural vs. surgical postmenopause and psychological symptoms confound the effect of menopause on executive functioning domains of cognitive experience	Maturitas, 2023	Wrong outcomes
Rocca 2023	Frequency and type of premature or early menopause in a geographically defined American population.	Maturitas, 2023	Wrong outcomes
Song 2023	Bilateral Oophorectomy and Colorectal Cancer Incidence and Mortality in the Women's Health Initiative.	Cancer Epidemiol Biomarkers Prev, 2023	Wrong outcomes
Blumel 2024	Genitourinary symptoms and sexual function in women with primary ovarian insufficiency.	Climacteric : the journal of the International Menopause Society, 2024	Wrong outcomes
Calvo 2024	Associated risk and resilience factors of Alzheimer's disease in women with early bilateral oophorectomy: Data from the UK Biobank.	Journal of Alzheimer's disease : JAD, 2024	Wrong outcomes
Dobson 2024	Menopause age and type and dementia risk: a pooled analysis of 233 802 women.	Age and ageing, 2024	Wrong outcomes
Espinoza 2024	Association between type of menopause and mild cognitive impairment: The REDLINC XII study.	Maturitas, 2024	Wrong outcomes
Fernandez-Pena 2024	Previous pregnancies might mitigate cortical brain differences associated with surgical menopause.	Human brain mapping, 2024	Wrong outcomes
Mielke 2024	Premenopausal bilateral oophorectomy and brain white matter brain integrity in later-life.	Alzheimer's & dementia : the journal of the Alzheimer's Association, 2024	Wrong outcomes
Vallejo 2024	Association of muscle disorders in late postmenopausal women according to the type of experienced menopause.	Menopause (New York, N.Y.), 2024	Wrong outcomes
Wu 2024	The role of sex hormone binding globulin levels in the association of surgical and natural premature menopause with incident type 2 diabetes.	Maturitas, 2024	Wrong outcomes
Haddadi 2025	The sleep quality in women with surgical menopause compared to natural menopause based on Ardakan Cohort Study on Aging (ACSA).	BMC women's health, 2025	Wrong outcomes

© 2026 Armeni E. *JAMA Network Open*. This is an open access article distributed under the terms of the CC-BY-NC-ND License, which does not permit alteration or commercial use, including those for text and data mining, AI training, and similar technologies.

PriceSAL 2025	What Happens After Menopause (WHAM)? A Progress Report of a Prospective Controlled Study of Women After Pre-Menopausal Risk-Reducing Bilateral Salpingo-Oophorectomy.	BJOG, 2025	Wrong outcomes
Blum 1979	The Vaginal Flora after Natural or Surgical Menopause	Journal of the American Geriatrics Society, 1979	Wrong patient population
Noto 2000	Blood pressure variations assessed by continuous 24-hour monitoring in menopausal and climacteric women.	Eur Rev Med Pharmacol Sci, 2000	Wrong patient population
Sowers 2001	The association of menopause and physical functioning in women at midlife.	J Am Geriatr Soc, 2001	Wrong patient population
Hayırlıoglu 2006	The effect of bilateral oophorectomy on bone mineral density	Rheumatology International, 2006	Wrong patient population
Verhoeven 2009	The influence of physiological and surgical menopause on coronary heart disease risk markers	Menopause, 2009	Wrong patient population
Fletcher 2010	Cardiovascular disease risk factors in menopausal Jamaican black women after hysterectomy and bilateral oophorectomy: An observational study	West Indian Medical Journal, 2010	Wrong patient population
Nichols 2011	Bilateral oophorectomy in relation to risk of postmenopausal breast cancer: Confounding by nonmalignant indications for surgery?	American Journal of Epidemiology, 2011	Wrong patient population
Neufeld 2015	Autonomic control of cardiovascular system in pre- and postmenopausal women: A cross-sectional study	Journal of the Turkish German Gynecology Association, 2015	Wrong patient population
Choi 2019	Increased risk of osteoporosis with hysterectomy: A longitudinal follow-up study using a national sample cohort	American Journal of Obstetrics and Gynecology, 2019	Wrong patient population
Lin 2020	Association between oophorectomy and depression in patients with comorbidities: A nationwide cohort study in Taiwan	Taiwanese Journal of Obstetrics and Gynecology, 2020	Wrong patient population
Rani 2022	Evaluation of early Menopause symptoms in Post-Hysterectomy and Premature Ovarian insufficiency in women of reproductive age group	Research Journal of Pharmacy and Technology, 2022	Wrong patient population
Beekman 2025	No increased arterial stiffness after premenopausal risk-reducing salpingo-oophorectomy (RRSO).	Maturitas, 2025	Wrong patient population
Pansini 1993	Influence of spontaneous and surgical menopause on atherogenic metabolic risk	Maturitas, 1993	Wrong study design
Kritz-Silverstein 1997	Hysterectomy, oophorectomy, and heart disease risk factors in older women	American Journal of Public Health, 1997	Wrong study design
Ikenoue 1999	Small low-density lipoprotein particles in women with natural or surgically induced menopause	Obstetrics and Gynecology, 1999	Wrong study design
Casiglia 2000	Blood pressure and metabolic profile after surgical menopause: Comparison with fertile and naturally-menopausal women	Journal of Human Hypertension, 2000	Wrong study design

© 2026 Armeni E. *JAMA Network Open*. This is an open access article distributed under the terms of the CC-BY-NC-ND License, which does not permit alteration or commercial use, including those for text and data mining, AI training, and similar technologies.

Pansini 2000	Sociodemographic and clinical factors associated with HRT use in women attending menopause clinics in Italy	Climacteric, 2000	Wrong study design
Schwab 2000	Risk of coronary heart disease, dietary fat modification, stages of change, and self-efficacy in surgically and naturally postmenopausal women	Journal of Women's Health and Gender-Based Medicine, 2000	Wrong study design
DeAloysio 2001	General and medical factors associated with hormone replacement therapy among women attending menopause clinics in Italy	Menopause, 2001	Wrong study design
Dwyer 2002	Carotid wall thickness and years since bilateral oophorectomy the Los Angeles Atherosclerosis Study	American Journal of Epidemiology, 2002	Wrong study design
Hsia 2003	Usefulness of prior hysterectomy as an independent predictor of Framingham risk score (The Women's Health Initiative)	American Journal of Cardiology, 2003	Wrong study design
Parazzini 2003	Determinants of body mass index in women around menopause attending menopause clinics in Italy	Climacteric, 2003	Wrong study design
Aldrighi 2004	Predictors of hot flushes in postmenopausal women who receive raloxifene therapy.	Am J Obstet Gynecol, 2004	Wrong study design
Manzoli 2004	Use of hormone replacement therapy in Italian women aged 50-70 years.	Maturitas, 2004	Wrong study design
Parazzini 2004	Factors associated with total cholesterol levels in women around menopause attending menopause clinics in Italy	Climacteric, 2004	Wrong study design
Bernis 2007	Environmental contexts of menopause in Spain: comparative results from recent research.	Menopause, 2007	Wrong study design
Yang 2007	A clinical trial of 3 doses of transdermal 17beta-estradiol for preventing postmenopausal bone loss: a preliminary study	2007	Wrong study design
Parazzini 2008	Trends of determinants of hormone therapy use in Italian women attending menopause clinics, 1997-2003	Menopause, 2008	Wrong study design
Michelsen 2009	Metabolic syndrome after risk-reducing salpingo-oophorectomy in women at high risk for hereditary breast ovarian cancer: A controlled observational study	European Journal of Cancer, 2009	Wrong study design
Tuna 2010	Variations in blood lipid profile, thrombotic system, arterial elasticity and psychosexual parameters in the cases of surgical and natural menopause	Australian and New Zealand Journal of Obstetrics and Gynaecology, 2010	Wrong study design
Cakir 2011	Comparison of the effects of surgical and natural menopause on epicardial fat thickness and γ-glutamyltransferase level	Menopause, 2011	Wrong study design
Jacoby 2011	Oophorectomy vs Ovarian Conservation With Hysterectomy: Cardiovascular Disease, Hip Fracture, and Cancer in the Women's Health Initiative Observational Study	JAMA Internal Medicine, 2011	Wrong study design

© 2026 Armeni E. *JAMA Network Open*. This is an open access article distributed under the terms of the CC-BY-NC-ND License, which does not permit alteration or commercial use, including those for text and data mining, AI training, and similar technologies.

Shufelt 2011	Timing of hormone therapy, type of menopause, and coronary disease in women: data from the National Heart, Lung, and Blood Institute-sponsored Women's Ischemia Syndrome Evaluation.	Menopause (New York, N.Y.), 2011	Wrong study design
Maserejian 2012	Sexual arousal and lubrication problems in women with clinically diagnosed hypoactive sexual desire disorder: Preliminary findings from the Hypoactive Sexual Desire Disorder Registry for Women.	Journal of Sex & Marital Therapy, 2012	Wrong study design
Matthews 2013	Changes in cardiovascular risk factors by hysterectomy status with and without oophorectomy: Study of Women's Health Across the Nation.	J Am Coll Cardiol, 2013	Wrong study design
McCarthy 2013	Association of bilateral oophorectomy and body fatness in a representative sample of US women	Gynecologic Oncology, 2013	Wrong study design
Parker 2013	Long-term mortality associated with oophorectomy compared with ovarian conservation in the nurses' health study.	Obstet Gynecol, 2013	Wrong study design
Sarrel 2013	The mortality toll of estrogen avoidance: an analysis of excess deaths among hysterectomized women aged 50 to 59 years	American Journal of Public Health, 2013	Wrong study design
Finch 2014	Impact of oophorectomy on cancer incidence and mortality in women with a BRCA1 or BRCA2 mutation.	Journal of clinical oncology : official journal of the American Society of Clinical Oncology, 2014	Wrong study design
Appiah 2015	Is Surgical Menopause Associated With Future Levels of Cardiovascular Risk Factor Independent of Antecedent Levels? The CARDIA Study.	Am J Epidemiol, 2015	Wrong study design
Farahmand 2015	Surgical menopause versus natural menopause and cardio-metabolic disturbances: A 12-year population-based cohort study	Journal of Endocrinological Investigation, 2015	Wrong study design
Sari 2016	Evaluation of cardiovascular disease risk in women with surgically induced menopause	Gynecological Endocrinology, 2016	Wrong study design
Worsley 2016	Low use of effective and safe therapies for moderate to severe menopausal symptoms: A cross-sectional community study of Australian women	Menopause, 2016	Wrong study design
Appiah 2017	The association of surgical versus natural menopause with future left ventricular structure and function: The Coronary Artery Risk Development in Young Adults (CARDIA) Study	Menopause, 2017	Wrong study design
Farahmand 2017	Adverse cardiometabolic effect in bilateral/unilateral oophorectomy versus natural menopause: Results of over a decade follow-up among Iranian women	Archives of Iranian Medicine, 2017	Wrong study design

© 2026 Armeni E. *JAMA Network Open*. This is an open access article distributed under the terms of the CC-BY-NC-ND License, which does not permit alteration or commercial use, including those for text and data mining, AI training, and similar technologies.

Mytton 2017	Removal of all ovarian tissue versus conserving ovarian tissue at time of hysterectomy in premenopausal patients with benign disease: study using routine data and data linkage.	BMJ, 2017	Wrong study design
deKat 2017	Unraveling the associations of age and menopause with cardiovascular risk factors in a large population-based study	BMC Medicine, 2017	Wrong study design
Khoudary 2018	HDL (High-Density Lipoprotein) metrics and atherosclerotic risk in women do menopause characteristics matter? MESA	Arteriosclerosis, Thrombosis, and Vascular Biology, 2018	Wrong study design
Dam 2019	Association of menopausal characteristics and risk of coronary heart disease: a pan-European case-cohort analysis.	Int J Epidemiol, 2019	Wrong study design
Teixeira 2019	Cholesterol profile in women with premature menopause after risk reducing salpingo-oophorectomy	Familial Cancer, 2019	Wrong study design
Cusimano 2021	Association of bilateral salpingo-oophorectomy with all cause and cause specific mortality: Population based cohort study	The BMJ, 2021	Wrong study design
Karia 2021	Association of Oophorectomy and Fat and Lean Body Mass: Evidence from a Population-Based Sample of U.S. Women.	Cancer epidemiology, biomarkers & prevention : a publication of the American Association for Cancer Research, cosponsored by the American Society of Preventive Oncology, 2021	Wrong study design
Price 2021	Early and surgical menopause associated with higher Framingham Risk Scores for cardiovascular disease in the Canadian Longitudinal Study on Aging.	Menopause (New York, N.Y.), 2021	Wrong study design
Kapoor 2022	Trajectories of metabolic parameters after bilateral oophorectomy in premenopausal women.	Maturitas, 2022	Wrong study design
Qian 2022	Early Menopause May Associate With a Higher Risk of CKD and All-Cause Mortality in Postmenopausal Women: An Analysis of NHANES, 1999–2014	Frontiers in Medicine, 2022	Wrong study design
Chirinos 2024	Trajectories of depressive symptoms in a population-based cohort of Black and White women from late reproductive age through the menopause transition: a 30-year analysis.	Menopause (New York, N.Y.), 2024	Wrong study design
Doust 2024	Assessing the Accuracy of Cardiovascular Disease Prediction Using Female-Specific Risk Factors in Women Aged 45 to 69 Years in the UK Biobank Study.	Circulation. Cardiovascular quality and outcomes, 2024	Wrong study design
Asinaro 2025	Menopausal symptoms in breast cancer survivors on adjuvant endocrine therapy compared with those of menopausal women.	Maturitas, 2025	Wrong study design

© 2026 Armeni E. *JAMA Network Open*. This is an open access article distributed under the terms of the CC-BY-NC-ND License, which does not permit alteration or commercial use, including those for text and data mining, AI training, and similar technologies.
