# Supplementary material for: Age at Iatrogenic Menopause and Cardiovascular Morbidity and Mortality: A Systematic Review and Meta-Analysis
Source: JAMA Netw Open. 2026 Oct 2;9(10):e2637545. doi: 10.1001/jamanetworkopen.2026.37545 (PMC13633536; doi:10.1001/jamanetworkopen.2026.37545)
Supplement: Supplementary file 2 [file jamanetwopen-e2637545-s002.pdf]

Data Sharing Statement

Armeni. Age at Iatrogenic Menopause and Cardiovascular Morbidity and Mortality. *JAMA Netw Open*. Published October 02, 2026. doi:10.1001/jamanetworkopen.2026.37545

Data
Data available: No

Additional Information
Explanation for why data not available: This work is based exclusively on data extracted from published literature. All underlying data are available in the cited articles, and no new individual participant data or datasets were generated. Therefore, no additional data are available for sharing.
